# Longitudinal characterization of mosaic chromosomal alterations identifies factors influencing clonal dynamics of leukocytes

**DOI:** 10.1038/s41467-026-75996-5

**Published:** 2026-07-29

**Authors:** Rebecca L. Kelly, Derek W. Brown, Weiyin Zhou, Aubrey K. Hubbard, Corey D. Young, Kara M. Barnao, Alyssa Klein, Diptavo Dutta, Aurélie Vogt, Jia Liu, Jiahui Wang, Wen-Yi Huang, Neal D. Freedman, Stephen J. Chanock, Paul S. Albert, Mitchell J. Machiela

**Affiliations:** 1 Cancer Prevention Fellowship Program, National Cancer Institute, NIH, Rockville, MD USA; 2 Division of Cancer Epidemiology and Genetics, National Cancer Institute, NIH, Rockville, MD USA; 3 Cancer Genomics Research Laboratory, Frederick National Laboratory for Cancer Research, Frederick, MD USA; 4 Division of Cancer Control and Population Sciences, National Cancer Institute, NIH, Rockville, MD USA

**Keywords:** Biomarkers, Cancer epidemiology, Genetics research

## Abstract

Characterizing the variation in clonal trajectory of age-related mosaic chromosomal alterations (mCAs) in circulating leukocytes can identify etiologic factors influencing clonal expansion and hematologic malignancy risk. Here we scan whole blood-derived DNA of 56,324 participants from the Prostate, Lung, Colorectal, Ovarian Cancer Screening Study for mCAs and characterize 1746 longitudinal mCAs. Cross-sectional mCA clonal fraction only moderately correlates with clonal expansion rate, highlighting the utility of serial sampling for evaluating clonal trajectory. The strongest contributor to clonal expansion is genomic location of an mCA with participant age, smoking status, and co-occurring mCA types also modifying expansion rates. Germline susceptibility to mosaic Y or X loss is not associated with expansion rate, supporting independent mechanisms governing generation and expansion. Autosomal mCAs previously associated with hematologic malignancies have the highest rates of expansion, especially myeloid malignancy-associated mCAs, underscoring the importance of tracing mCA clonal dynamics for evaluating hematologic cancer risk.

## Abstract (web summary)

Mosaic chromosomal alterations in blood cells increase with age, but their expansion over time varies widely between individuals and genomic regions. Here, the authors analyze longitudinal blood samples to show that chromosomal location, age, smoking status, and links to blood cancer lineages influence clonal expansion, while the influence of inherited susceptibility was limited.

## Introduction

The hematopoietic compartment is a dynamic tissue originating from a limited number of stem cells which differentiate to produce specialized cells needed for immune response and homeostasis. Over time, stem cells acquire somatic alterations which can lead to populations of leukocytes that are genetically distinct, a phenomenon termed clonal hematopoiesis (CH). Though many risk factors associated with the prevalence of CH have been identified, those influencing the rate or trajectory of CH clonal expansion are not well characterized, in part due to limited longitudinal investigations. Clonal expansion of CH is governed by the intrinsic clonal fitness imparted by the alteration and an environment that favors the new alteration^1^. Given that larger clone sizes have been associated with increased risk of hematologic malignancy^2^, greater disease severity and higher mortality risk^3,4^, a more complete understanding of the clonal dynamics and heterogeneity of expansion for specific CH subtypes is needed to identify etiologic factors governing clonal expansion and inform the utility of specific mCAs as biomarkers of disease risk and progression.

CH can be driven by single-nucleotide variants (SNVs), structural variants, or epigenetic mechanisms. Somatic SNVs occurring in known leukemia-related driver genes (e.g., *DNMT3A*, *TET2*, *ASXL1*) with variant allele frequencies greater than 0.01 are referred to as clonal hematopoiesis of indeterminant potential (CHIP) and are strongly associated with hematopoietic malignancy risk^5–7^. A characterization of CHIP in 4187 middle aged participants identified an association between increasing age and expansion rate^8^ and a longitudinal cohort of 385 individuals refined mutation-specific effects of age on CHIP clonal expansion^7^, suggesting heterogeneity of expansion across specific types of CH and underscoring the need for well-powered studies to separately characterize specific types of CH.

Despite these longitudinal studies of CHIP-based CH, there is a dearth of longitudinal data regarding larger structural somatic alterations (e.g., generally > 50 KB), collectively termed mosaic chromosomal alterations (mCAs), which consist of chromosomal gains, losses and imbalances of maternal and paternal alleles called copy-neutral loss of heterozygosity (CN-LOH). mCAs are age-related, with population frequencies of autosomal mCAs estimated at 6–10%, loss of X chromosome (mLOX) at 5–12% in females, and loss of Y chromosome (mLOY) surpassing 20% in older males^3,9^. Prevalence of specific mCAs has been associated with environmental exposures^10,11^, clinical and behavioral factors^12^, and germline genetic variation^5,13–15^. Various methods have been applied to cross-sectional mCA clonal fraction (CF) data to infer clonal fitness of specific mCA events^16,17^. Watson and Blundell adapted an evolutionary framework to distinguish mutation rate and fitness effects for frequent mCAs and investigated mCA fitness effects that deviated from expectation^18^. Higher fitness effects of mCAs correlated with risk of hematologic malignancy, mirroring a similar relationship previously observed between higher expansion rates and hematologic cancer risk observed in studies of CHIP^16^. Pershad and colleagues also investigated cross-sectional mCA data using the *PACER* method, which uses passenger variant allele frequencies from somatic SNVs to infer clonal expansion rates^17^. They identified a variant at the *TCL1A* locus associated with mCA expansion rate, which is in high linkage disequilibrium with variant rs2887399, previously identified as a susceptibility locus for mLOY predisposition^15^, suggesting germline influences on clonal expansion of mCAs^17^.

In this work, we show that longitudinal observations of mCAs from serial samples enable identification of sources responsible for heterogeneity in expansion across individuals. Here, we describe mCAs detected in blood-derived samples collected from a large, prospective cohort of 56,324 participants and a detailed characterization of mCAs occurring in 1490 individuals with longitudinal mCAs from serial samples. We quantify mCA expansion rates across chronological age and evaluate the influence of individual factors on expansion rate. We observe differences in mCA clonal expansion by chromosomal location and relationship with hematologic malignancy lineage, uncover associations of age and smoking behaviors with clonal expansion and evaluate if the rate of clonal expansion of mCAs is different in individuals who were subsequently diagnosed with a hematologic malignancy.

## Results

### PLCO Cross-sectional mCAs

Of the 56,324 PLCO participants with genotyping data from blood-derived DNA who were screened for mCAs, 10,747 (19.1%) participants had detectable mCAs (Table 1) in line with expectations from prior population-based investigations of adult populations^9,19^. Individuals with detectable mCAs were on average older (67 years, *P-value*(*P)* < 0.001), more likely to be male (*N* = 6235, 58.0%, *P* < 0.001), and more likely to be current smokers (*N* = 1287, 12.0%, *P* < 0.001) (Table 1). The most frequently detected mCA was mLOY in males (50.0% of individuals with an mCA), followed by mLOX in females (33.1% of individuals with an mCA) (Table 1). The remaining mCAs included autosomal gains, losses, and CN-LOH (20.3% of individuals), gains, losses, and CN-LOH of the X chromosome < 100 Mb in females (1.2% of individuals), and Y chromosome gains in males (0.3% of individuals). Six percent of individuals with an mCA (*N* = 676) had more than one mCA. CF of prevalent autosomal mCAs and mLOY ranged from 0.005–0.812 and 0.010–0.720, respectively, and had positive associations with age (autosomal_beta _= 2.85, *P* = 0.0067; mLOY_beta_ = 8.62, *P* = 3.99 × 10^−59^) (Supplementary Fig. 1). mLOX CF ranged from 0.003-0.648 but was not related to age in the cross-sectional cohort (mLOX_beta _= 2.98, *P* = 0.1405).

**Table 1:** PLCO Cohort Participant Characteristics

Characteristic	PLCO Individuals Scanned for mCAs	PLCO Individuals with Cross Sectional mCAs	PLCO Individuals with Longitudinal Assessment
	(*N* = 56,324)	(*N* = 10,747)	(*N* = 1490)
Age at Genotyping (mean(SD))	65.30 (5.35)	67.17 (5.43)	64.3 (5.60)
Sex (column %)			
Female	28,042 (49.8)	4512 (42.0)	469 (31.5)
Male	28,282 (50.2)	6235 (58.0)	1021 (68.5)
Smoking Status (column %)			
Never	26,476 (47.0)	4722 (43.9)	605 (40.4)
Current	5480 (9.7)	1287 (12.0)	181 (12.1)
Former	24,122 (42.8)	4702 (43.8)	712 (47.5)
Unknown	246 (0.4)	36 (0.3)	0 (0.0)
BMI (column %)			
< 25	18,376 (32.6)	3590 (33.4)	481 (32.1)
25-30	23,613 (41.9)	4720 (43.9)	720 (48.1)
30-35	9570 (17.0)	1683 (15.7)	213 (14.2)
> 35	4752 (8.4)	753 (7.0)	73 (4.9)
Unknown	13 (0.0)	1 (0.0)	11 (0.7)
Individuals with mCA Type* (column %)			
Autosomal	–	2179 (20.3)	671 (45.0)
mLOX	–	3559 (33.1)	213 (14.3)
mLOY	–	5355 (50.0)	834 (56.0)
X Chromosome mCA	–	131 (1.2)	42 (2.8)
Y Gain	–	32 (0.3)	10 (0.7)
Individuals with Multiple mCAs (column %)	–	676 (6.3)	467 (31.3)

Characteristics of the individuals in the PLCO study with blood-based genotyping (*N* = 56,324), those individuals from the blood-based genotyping with detectable mCAs (*N* = 10,747), and the individuals included in the cohort for longitudinal characterization (*N* = 1490). SD = Standard Deviation, * = Column percentage mCAs by type is greater than 100 due to individuals with multiple mCAs.

We observed enrichment of specific autosomal mCAs in the cross-sectional PLCO cohort. CN-LOH on 14q was the most frequently observed event (N = 124), followed by loss of 13q (*N* = 91), and gain of chromosome 12 (*N* = 87) (Supplementary Fig. 2). The autosomal mCAs with the highest median CF were CN-LOH of 9p (*N* = 17; median CF = 0.130), loss of 20q (*N* = 66; median CF = 0.130), and loss of 13q (median CF = 0.121) (Supplementary Fig. 2).

### PLCO Longitudinal mCAs

From the cross-sectional characterization of PLCO, we selected individuals with detectable mCAs having DNA available from 2 to 4 serial samples for longitudinal characterization (Supplementary Fig. 3A). The average time between the first blood sample and last blood sample for an individual was 4.2 years, with a maximum follow-up of six years. The average age of an individual at their first sample was 64 years of age. The individuals with mCAs selected for longitudinal characterization were slightly enriched with males (68.5% vs. 58.0%, *P* < 0.001) and overweight individuals (BMI between 25 and 30, 48.1% vs. 43.9%, *P* < 0.001), but had fewer class 2 and 3 obese individuals (BMI above 35, 4.9% vs. 7.0%, *P* < 0.001) compared to individuals in the cross-sectional investigation with mCAs (Table 1). Individuals with autosomal mCAs (45.0% vs. 20.3%, *P* < 0.001) were more enriched in the longitudinal analysis, and individuals with mLOX (14.3% vs. 33.1%, *P* < 0.001) were less common. The longitudinal sample was enriched for co-occurring mCAs, with 31.3% of individuals in the longitudinal analysis having two or more mCAs, with up to 19 mCAs observed in a single individual (Supplementary Fig. 3B).

There were a total of 2294 mCAs observed across multiple samples for the 1490 individuals with longitudinal assessment (Table 2 and Supplementary Fig. 3C). The majority of mCAs were detected at the individual’s first sample (71.7% prevalent mCAs, *P* < 0.001), with less than a third of mCAs first detected in a subsequent sample (28.3% incident mCAs). Eighty-five individuals acquired their first mCAs during the sampling window, with these individuals more likely to be current smokers (*P* = 0.0127) than mCA-free individuals.

**Table 2:** Longitudinal Cohort mCA Characteristics

mCA Type	Autosomal	mLOX	mLOY	X Events	Y Gains	Total
**N**	**1179**	**213**	**834**	**58**	**10**	**2294**
Status (column %)						
Prevalent	655 (55.6)	178 (83.6)	778 (93.3)	23 (39.7)	10 (100)	**1644 (71.7)**
Incident	524 (44.4)	35 (16.4)	56 (6.7)	35 (60.3)	0 (0.0)	**650 (28.3)**
Observations (column %)						
Single timepoint	453 (38.4)	24 (11.3)	41 (4.9)	34 (58.6)	0 (0.0)	**548 (23.9)**
Multiple timepoints	726 (61.6)	189 (88.7)	793 (95.1)	24 (41.4)	10 (100)	**1746 (76.1)**

Table describing the types of mCAs observed, as well as the status of mCAs as incident or prevalent and whether the mCA was observed at only one or multiple timepoints.

### Effect of mCA-intrinisc factors on clonal expansion rate

Over two-thirds of mCAs were observed at multiple timepoints (*N* = 1746, 76.1%) and could be evaluated for their longitudinal dynamics (Table 2). Based on the abundance of autosomal mCAs (*N* = 726), mLOX (*N* = 189), and mLOY (*N* = 793) with multiple observations across samples, we considered these three mCA types for characterization. We evaluated initial trajectories by calculating the difference in clonal fraction between the last and the first observations of an mCA. Of these 1712 mCAs, 1391 (81.3%) had positive trajectories, with mLOY having the highest percentage of positive trajectories (93.3%) and mLOX having the lowest percentage of positive trajectories (65.6%) (Supplementary Table 1).

**Fig. 1:**
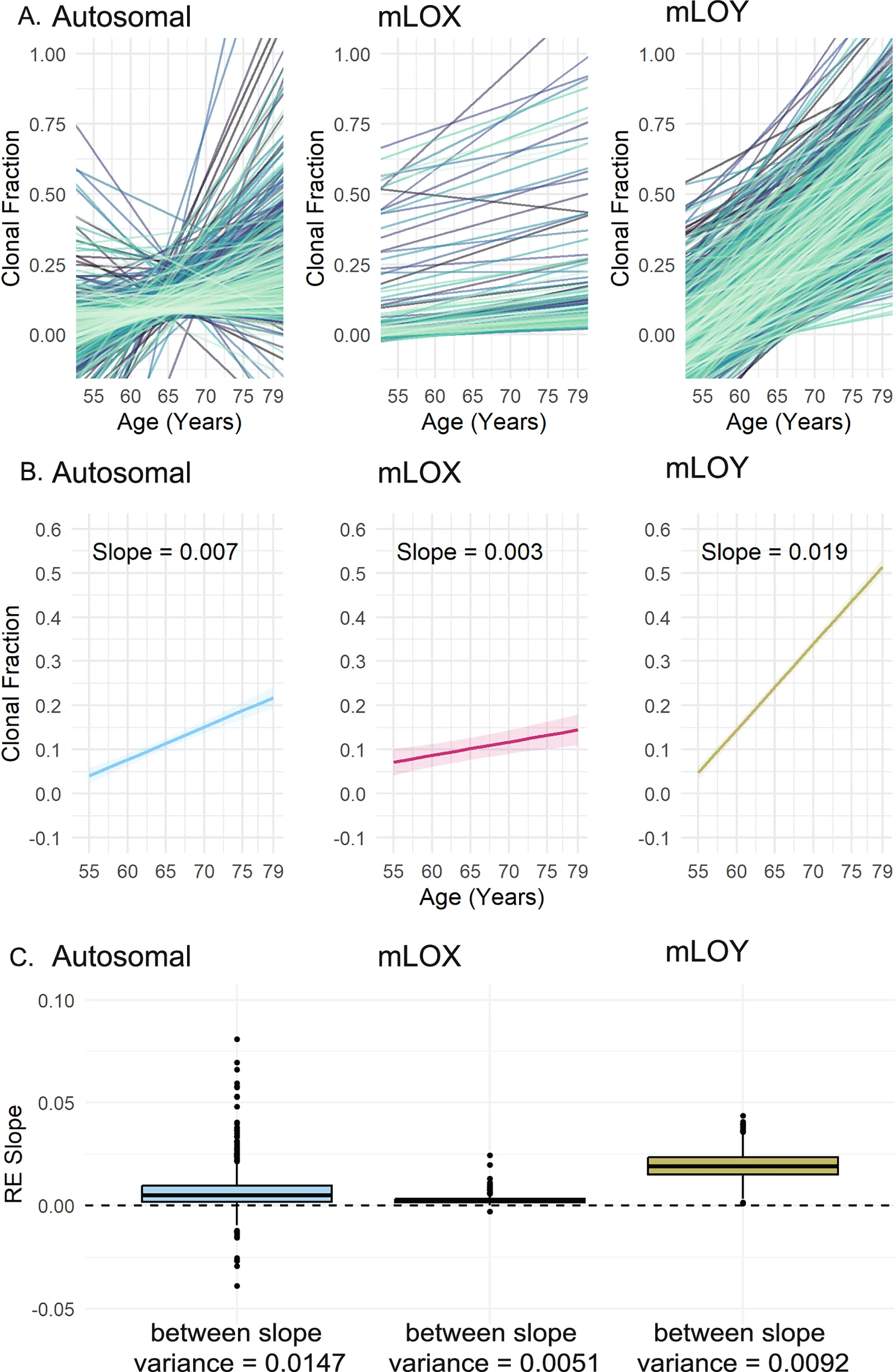
Population-level expansion rates of the three main mCA types. **A** Line plots of individual random effects (individual random effect plus fixed effect) from LMMs for mCA CF across age. Autosomal mCAs: *N* = 593 individuals; mLOX: *N* = 189 individuals; mLOY: *N* = 793 individuals. **B** Population average fixed effect profile plots of the models in (**A**), including the slope of clonal expansion per one year increase in age. The shaded band around the average slopes indicates the 95% CI of the mean trajectory, accounting for the longitudinal correlation structure. **C** Boxplots of individual random effects (RE) for the models shown in (**A**) as the median and interquartile range from the 25^th^ to 75^th^ percentiles, with individual points representing outliers. The inter-slope variations from the LMMs are shown below the boxplots. Source data are provided as a Source Data file. CF = clonal fraction, RE = random effect, LMM =  linear mixed model.

Since the longitudinal data are highly unbalanced (differing numbers and timing of measurements across participants), linear mixed models (LMMs) provide an efficient estimation of the population-average dynamics and individual-specific profiles as compared with individual least-square estimates^20^. Modeling the clonal expansion rate with linear mixed models, we observed substantial differences in the rate of clonal expansion by individual mCAs and mCA type (Fig. 1A). The per year increase in mLOY clonal expansion was the highest (1.95% per year age, 95% confidence interval (CI) = [1.90–2.00]), followed by autosomal mCAs (0.74% per year age, 95% CI = [0.59, 0.89] and mLOX (0.30% per year age, 95% CI = [0.13, 0.47]) (Fig. 1B). The high rate of clonal expansion for mLOY estimated in the longitudinal data corresponds with the high population frequencies and cell fractions of mLOY observed in the cross-sectional characterization. (Table 1 and Supplementary Fig. 1). This relationship did not hold for mLOX, frequently detected in females, as mLOX had a lower clonal expansion rate than autosomal mCAs and was observed at lower cell fractions in the cross-sectional characterization despite having a high frequency in the cross-sectional PLCO sample (12.7% of females).

**Fig. 2:**
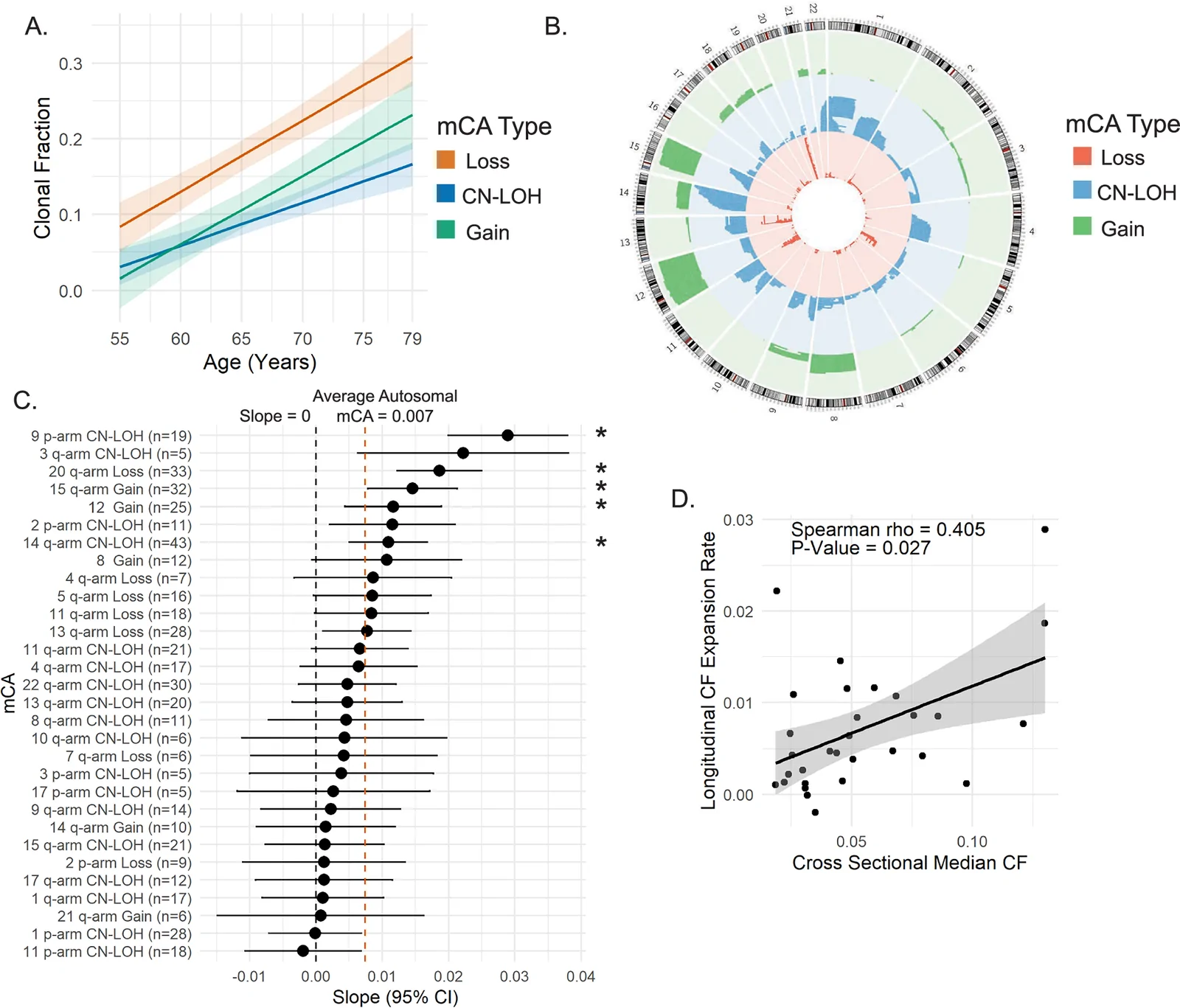
Autosomal mCA clonal expansion by type and chromosomal location. **A** Profile plot of population average autosomal mCAs clonal expansion by type. Losses *N* = 198 (orange), CN-LOH *N* = 390 (blue), Gains *N *= 138 (green). The shaded bands around the average slopes indicate the 95% CI of the mean trajectories. **B** Circos plot of the 726 longitudinal autosomal mCAs. Chromosomal number and position are indicated on the outside edge of the circle with autosomal mCA events as stacked colored bars: Gains (green), CN-LOH (blue), and Losses (orange). **C** Forest plot of beta coefficients and 95% CI for enriched autosomal mCAs by arm and chromosomal location. *P-Values* (*P*) come from a two-sided T-Test with Satterthwaite’s degrees of freedom for the beta coefficient of age at each level of mCA chromosome-arm-type. An asterisk (*) denotes a *P-Value* that reaches the FDR significance threshold of *P* ≤ 0.005. The black dotted line corresponds to slope = 0.00, and the orange dotted line corresponds to the population-average expansion rate of autosomal mCAs (0.007). **D** Spearman correlation of the clonal expansion rates from longitudinal cohort mCAs with the median CF from the cross-sectional PLCO population. *P-Value* from a two-sided exact test for Spearman’s rho. Source data are provided as a Source Data file.

There was highest between-subject variance in the random effects of autosomal mCA expansion (${\hat{\sigma }}_{0}$_autosomal_ = 0.0147; ${\hat{\sigma }}_{0}$_mLOX_ = 0.0051, ${\hat{\sigma }}_{0}$_mLOY_ = 0.0092, Fig. 1C), so we further characterized autosomal mCAs by possible sources of the heterogeneity including event type and chromosomal location. We observed an overall lower rate of clonal expansion for CN-LOH (0.57% per year age, 95% CI = [0.39, 0.75]) relative to autosomal losses (0.94% per year age, 95% CI = [0.69, 1.19], *P*_*interaction*_ = 0.0112) and gains (0.90% per year age, 95% CI = [0.61, 1.19], *P*_*interaction*_ = 0.0461) (Fig. 2A). As specific autosomal mCAs are recurrently detected in populations of aging individuals^21,22^, we extended our characterization to the chromosome level by examining the clonal trajectories of any recurrent mCA detected in ≥ 5 individuals in the serial sample group (Fig. 2B and Supplementary Fig. 4). The highest rates of clonal expansion across age were observed for CN-LOH on 9p (2.89% per year age, 95% CI = [2.00, 3.80], *P* = 5.24 × 10^−^^10^) and 3q (2.22% per year age, 95% CI = [0.63, 3.81], *P* = 0.006), copy number losses on 20q (1.86% per year age, 95% CI = [1.23, 2.50], *P* = 1.75 × 10^−^^8^), gains of chromosome 15q (1.46% per year age, 95% CI = [0.78, 2.13], *P* = 2.85 × 10^−^^5^), and gains of chromosome 12 (1.16% per year age, 95% CI = [0.43, 1.89], *P* = 0.0019) (Fig. 2C). The lowest rate of clonal expansion across age was observed for CN-LOH on 11p (− 0.20% per year age, 95% CI = [− 1.07, 0.70], *P* = 0.661).

We further investigated if the average population expansion rate of these mCAs corresponded to the CF observed in the population. Analyzing enriched autosomal mCA group-level estimates of median CF from the cross-sectional PLCO population data with the clonal expansion rates revealed a positive correlation (Spearman *R* = 0.405, *P* = 0.027) (Fig. 2D), indicating that the cross-sectional evaluation of mCAs cell fraction agrees with clonal expansion rates, but may not fully capture the variability in clonal trajectories.

**Fig. 3:**
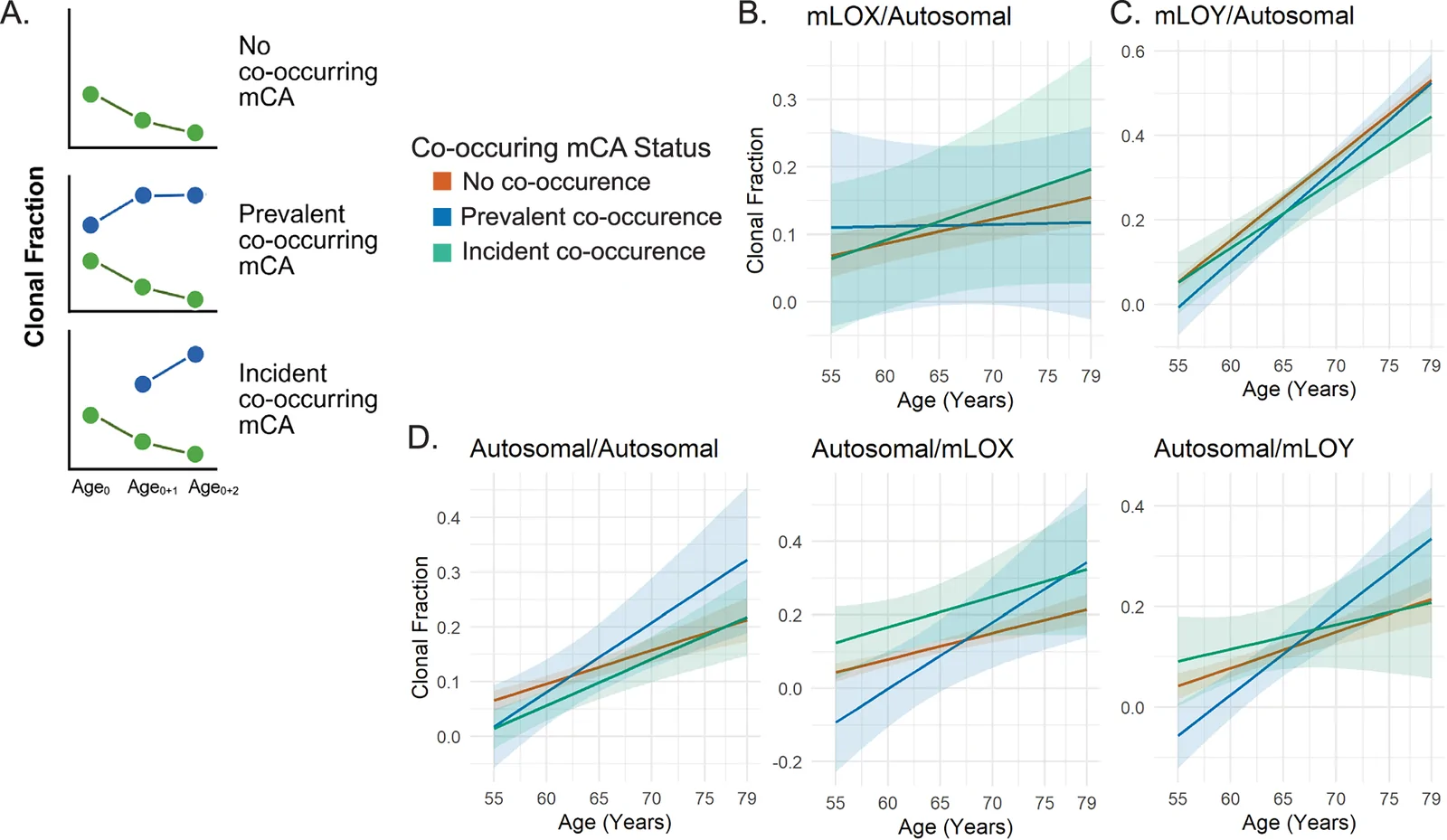
Expansion of mCAs by co-occurrence and temporal relationship. **A** Schematic depicting the relative temporal relationship between co-occurring mCAs. Top – no co-occurring mCA; middle – co-occurring mCAs are both observed in the same first sample; bottom – the co-occurring mCA is first observed after the initial mCA. **B** Profile plot of the population average rate of expansion of mLOX by co-occurring autosomal mCA timing: *N* = 151 (orange) mLOX only, *N* = 10 (blue) mLOX with prevalent co-occurring autosomal mCA; *N* = 10 (green) mLOX with incident co-occurring autosomal mCA. The shaded bands around the average slopes indicate the 95% CI of the mean trajectories. **C** Profile plot of the population average rate of expansion of mLOY by co-occurring autosomal mCA timing: *N* = 653 (orange) mLOY only, *N* = 47 (blue) mLOY with prevalent co-occurring autosomal mCA; *N* = 32 (green) mLOY with incident co-occurring autosomal mCA. The shaded bands around the average slopes indicate the 95% CI of the mean trajectory. **D** Profile plot of the population average rate of expansion of autosomal mCAs by: Left graph: co-occurring autosomal mCA timing: *N* = 219 (orange) autosomal only, *N *= 16 (blue) autosomal mCA with prevalent co-occurring autosomal mCA; *N* = 68 (green) autosomal mCA with incident co-occurring autosomal mCA; Middle graph: co-occurring mLOX mCA timing: *N *= 219 (orange) autosomal mCA only, *N* = 8 (blue) autosomal with prevalent co-occurring mLOX; *N* = 12 (green) autosomal mCA with incident co-occurring mLOX; Right graph: co-occurring mLOY timing: *N* = 219 (orange) autosomal mCA only, *N* = 39 (blue) autosomal mCA with prevalent co-occurring mLOY; *N* = 18 (green) autosomal mCA with incident co-occurring mLOY. The shaded bands around the average slopes indicate the 95% CI of the mean trajectories. Source data are provided as a Source Data file.

As 31.3% of individuals in the longitudinal cohort harbor multiple mCAs, we tested the relationship between co-occurring mCAs with rate of clonal expansion. We restricted the analysis of co-occurring mCAs to individuals with one or two mCAs observed during the study period and compared the rate of the mCA expansion in individuals with co-occurrence to those with only one mCA of that type (no co-occurrence). The temporal relationship between co-occurring mCAs was considered by identifying if co-occurring mCAs were observed together at an individual’s first mCA-positive sample (prevalent co-occurring) or if one of the mCAs was observed after the initial mCA (incident co-occurring) (Fig. 3A).

Among females with mLOX, expansion of mLOX with prevalent co-occurring autosomal mCAs was lower than individuals with no co-occurrence (rate_mLOX_prev_auto_ = 0.03% per year age, 95% CI = [− 0.68,0.74]; versus rate_mLOX_no_co-occur_ = 0.36% per year age, 95% CI = [0.17,0.54], *P*_*interaction*_ = 0.3825) and higher in individuals with an incident autosomal mCA (rate_mLOX_inc_auto_ = 0.55% per year age, 95% CI = [−0.16,0.1.26]; *P*_*interaction*_ = 0.6066), but neither relationship was statistically significant (Fig. 3B). The trend was the opposite for males with mLOY. Males with mLOY and prevalent autosomal mCAs had higher rates of expansion (rate_mLOY_prev_auto_ = 2.22% per year age, 95% CI = [1.81,2.62]; versus rate_mLOY_no_co-occur_ = 1.99% per year age, 95% CI = [1.88,2.10], *P*_*interaction*_ = 0.2820), and lower rates of expansion with incident autosomal mCAs (rate_mLOY_inc_auto_ = 1.64% per year age, 95% CI = [1.18,2.09]; *P*_*interaction*_ = 0.1420), but neither relationship was statistically significant (Fig. 3C).

For autosomal mCAs, autosomal mCAs with other prevalent co-occurring autosomal mCAs (rate_auto_prev_auto_ = 1.24% per year age, 95% CI = [0.54,1.95]; *P*_*interaction*_ = 0.0883) and incident co-occurring autosomal mCAs (rate_auto_inc_auto_ = 0.83% per year age, 95% CI = [0.47,1.19]; *P*_*interaction*_ = 0.2958) had higher rates of expansion compared to those with only one autosomal (rate_auto_no_co-occur_ = 0.61% per year age, 95% CI = [0.41,0.81] (Fig. 3D). For autosomal mCAs and prevalent and incident mLOX, the rate of autosomal mCA clonal expansion was higher in individuals with co-occurrence (rate_auto_prev_mLOX_ = 1.82% per year age, 95% CI = [0.67,2.96]; versus rate_auto_no_co-occur_ = 0.61% per year age, 95% CI = [0.41,0.81], *P*_*interaction*_ = 0.0658) and (rate_auto_inc_mLOX_ = 0.83% per year age, 95% CI = [−0.13,1.79]; *P*_*interaction*_ = 0.8175, but not significantly. For autosomal mCAs and prevalent mLOY, autosomal mCAs with prevalent mLOY co-occurrence had a higher expansion rate which was statistically significant at the FDR threshold (rate_auto_prev_mLOY_ = 1.64% per year age, 95% CI = [1.04, 2.23]; versus rate_auto_no_co-occur_ = 0.72% per year age, 95% CI = [0.47, 0.97], *P*_*interaction*_ = 0.0055) while the effect of incident mLOY did not significantly alter the rate of expansion (rate_auto_inc_mLOY_ = 0.49 % per year age, 95% CI = [−0.36, 1.34]; *P*_*interaction*_ = 0.6069). This difference in the effect of prevalent and incident mLOY on autosomal mCA expansion suggests that mLOY and autosomal mCAs could have cooperative expansion, but that newly acquired mLOY could have a disruptive impact on autosomal mCA clonal expansion.

### Effect of individual factors on clonal expansion

**Fig. 4:**
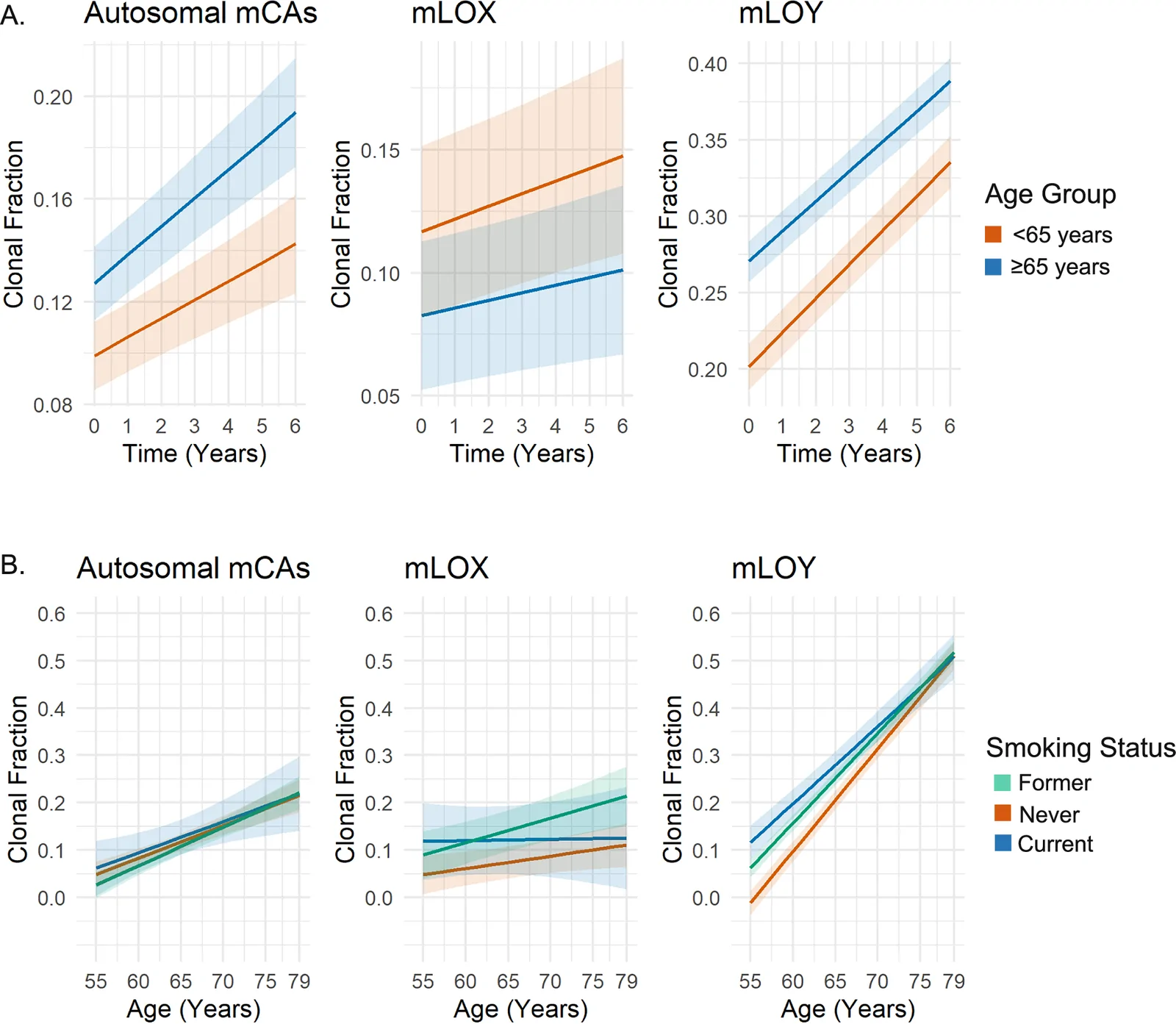
Effect of individual factors on mCA clonal expansion. **A** Profile plot of population average effects of age on mCA clonal expansion across years on study. Autosomal mCAs: < 65 *N* = 380 (orange), ≥ 65 *N* = 346 (blue), mLOX: < 65 N = 81 (orange), ≥ 65 *N* = 108 (blue); mLOY: < 65 *N* = 347 (orange), ≥ 65 *N* = 446 (blue). The shaded bands around the average slopes indicate the 95% CI of the mean trajectories. **B** Profile plot of population average effect of smoking status on clonal expansion across age. Autosomal: Never smoker *N* = 337 (orange), Current smoker *N* = 71 (blue), Former smoker *N* = 318 (green); mLOX: Never smoker *N* = 105 (orange), Current smoker *N* = 22 (blue), Former smoker *N* = 62 (green); mLOY: Never smoker *N* = 260 (orange), Current smoker *N* = 112 (blue), Former smoker *N* = 421 (green). The shaded bands around the average slopes indicate the 95% CI of the mean trajectories. Source data are provided as a Source Data file.

We also used the LMM approach to estimate the influence of individual factors such as age, sex, smoking status and BMI on clonal expansion of the three most abundant mCA types (i.e., mLOY, mLOX and autosomal mCAs). Age greater than 65 at the first observed mCA timepoint had a significant, positive interaction with the yearly clonal expansion of autosomal mCAs (rate_≥65_ = 1.11% per year, 95% CI = [0.85, 1.37] vs. rate_<65_ = 0.73 % per year, 95% CI = [0.50, 0.95], *P*_*interaction*_ = 0.030) over the six-year course of longitudinal characterization (Fig. 4A). Interestingly, the mLOY yearly clonal expansion rate was lower in individuals older than 65 (rate_≥65_ = 1.97% per year age, 95% CI = [1.83, 2.11] vs. rate_<65_ = 2.24% per year age, 95% CI = [2.09, 2.39], *P*_*interaction*_ = 0.0098). We did not observe a relationship of sex with clonal expansion of autosomal mCAs (Supplementary Fig. 5A) or for BMI with clonal expansion of all three main types of mCAs (Supplementary Fig. 5B).

Smoking did not significantly impact the expansion of autosomal mCAs or mLOX; however, a significant interaction with mLOY expansion was detected. Among males with mLOY, never smokers experienced the highest rate of clonal expansion (rate_never_smoke_ = 2.17% per year age, 95% CI = [2.01, 2.33]), while current smokers and former smokers had lower rates of expansion (rate_current_smoke_ = 1.63% per year age, 95% CI = [1.39, 1.88], *P*_*interaction*_ = 3.41 × 10^−^^4^) and (rate_former_smoke_ = 1.90% per year age, 95% CI = [1.77, 2.03], *P*_*interaction*_ = 0.009), respectively across age (Fig. 4B). It is notable that in models of >mLOY and smoking, current and former smokers had higher average initial cell fractions (intercept_current_smoke_ = 0.280, intercept_former_smoke_ = 0.252, intercept_never_smoke_ = 0.206), potentially limiting expansion rate. To investigate the impact of starting clonal fraction on mLOY expansion, we categorized mLOY trajectories by starting clonal fraction and compared the effects of mLOY starting clonal fraction (Supplementary Fig. 6A–C). We observed no difference in slope across starting clonal fraction. Though current smokers were enriched in higher starting clonal fraction bins, their rate of expansion was consistently lower than never smokers at every starting clonal fraction, further confirming that the impact of smoking is not due to a difference in starting mLOY clonal fraction (Supplementary Fig. 6D–F). The rate of expansion in former smokers showed some nominal differences across starting bins, suggesting there could be some changes in rates of expansion in former smokers depending on their starting clonal fraction bin. These relationships between individual-level factors and clonal expansion were similar in multivariable models containing all of the individual factors and their interactions with time, suggesting independent effects of these specific factors (Supplementary Table 2).

Given the strong relationship between germline genetic variation and the prevalence of mCAs^14,23,24^ (Supplementary Fig. 7), we tested the effect of polygenic risk of mLOX and mLOY on clonal expansion of mLOX and mLOY, respectively, as well as a telomere length polygenic score on the clonal expansion of mLOX, mLOY and autosomal mCAs. We observed no significant differences in the effect of polygenic risk quartile on the rate of clonal expansion for the tested scores and outcomes (Supplementary Fig. 8A, B), suggesting a limited impact of known scores for germline susceptibility for prevalent mCAs on the clonal expansion rate of mCAs. We also considered genotype at rs1122138 near *TCL1A* for its influence on clonal expansion based on evidence from cross-sectional methods for assessing mCA fitness^17^, but we had few individuals heterozygous (*N* = 338) or homozygous (*N* = 20) for the risk allele in the longitudinal cohort and did not observe any significant interactions of genotype with clonal expansion (Supplementary Fig. 8C).

### Malignancy-related mCAs and subsequent cancer diagnoses

**Fig. 5:**
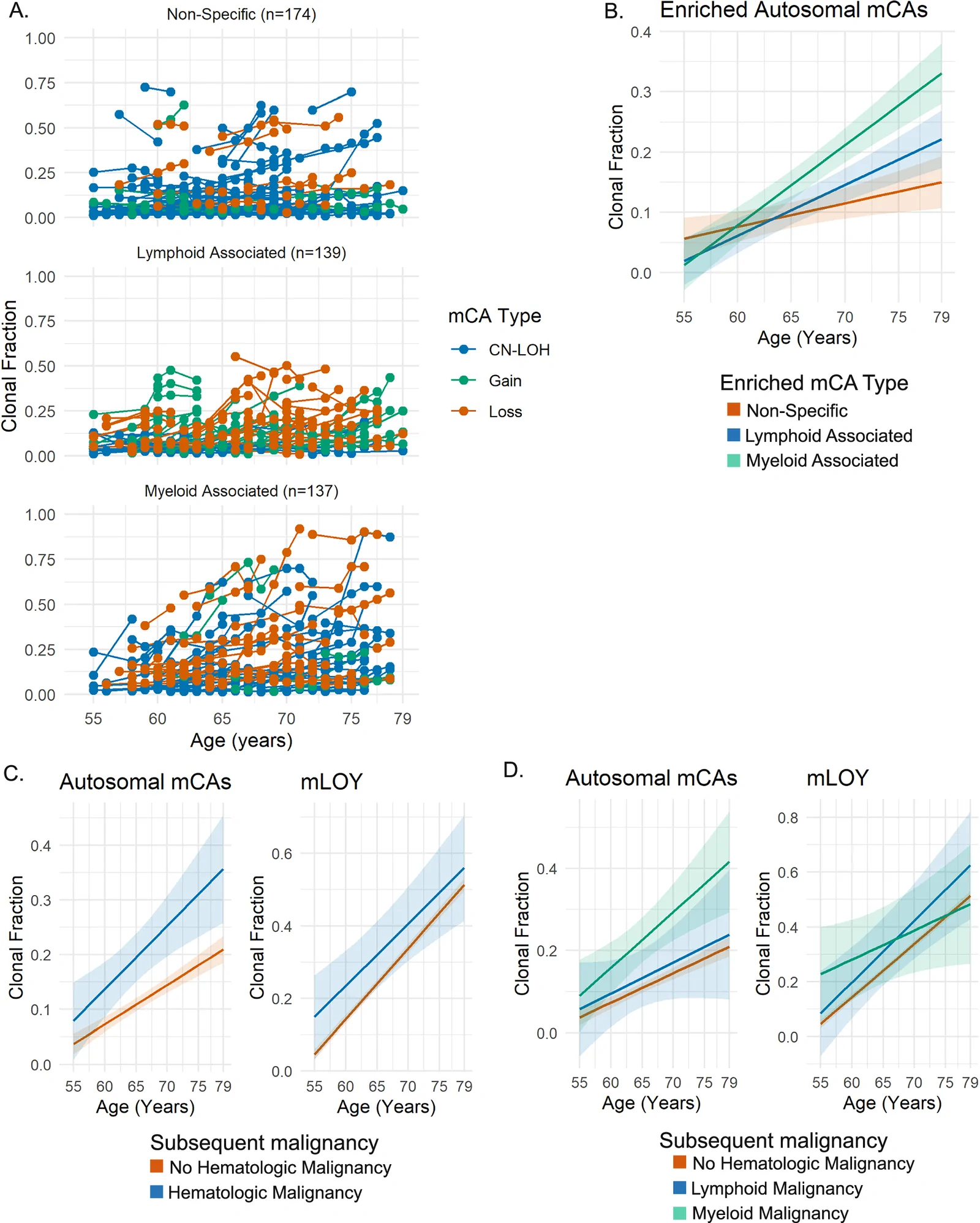
Malignancy-associated autosomal mCAs and subsequent cancer diagnoses. **A** Line plots of mCAs stratified by the associated lineage of hematologic malignancy based on Niroula and colleagues 2021. **B** Profile plots for enriched autosomal mCAs and their interaction with associated malignancy type. Non-specific *N* = 174 (orange), Lymphoid *N* = 139 (blue), Myeloid *N* = 137 (green). **C** Profile plots for autosomal mCAs and mLOY in individuals with diagnosed hematologic malignancies subsequent to the PLCO longitudinal sampling. Autosomal mCAs: No hematologic malignancy *N* = 556 (orange), hematologic malignancy *N* = 36 (blue); mLOY: No hematologic malignancy *N* = 782 (orange), hematologic malignancy *N* = 11 (blue). The shaded bands around the average slopes indicate the 95% CI of the mean trajectories. **D** Profile plots for population average effect of subsequent diagnosis of hematologic malignancy by cell lineage of the subsequent malignancy on autosomal mCAs and mLOY clonal expansion. Autosomal mCAs: No hematologic malignancy *N* = 556 (orange), lymphoid malignancy *N* = 13 (blue), myeloid malignancy *N* = 23 (green); mLOY: No hematologic malignancy *N* = 782 (orange), lymphoid malignancy *N *= 6 (blue), myeloid malignancy *N* = 5 (green). The shaded bands around the average slopes indicate the 95% CI of the mean trajectory. Source data are provided as a Source Data file.

To investigate the relationship between hematologic malignancy risk and clonal expansion rate, we measured both the clonal expansion rate of enriched autosomal mCA types associated with malignancy risk and the clonal expansion rate of mCAs in individuals who were later diagnosed with hematologic malignancies. A study by Niroula and colleagues found relationships between specific mCA types and risk of hematologic malignancies occurring in the lymphoid and myeloid lineages^25^. We classified enriched mCAs in the longitudinal cohort based on the Niroula malignancy-specific lineage groups and measured the rate of clonal expansion for these groups (Fig. 5A and Supplementary Table 3). Compared to mCAs with no associated malignancy lineage type, malignancy-associated mCAs had higher expansion rates, especially for myeloid malignancy associated mCAs (rate_non-specific_ = 0.39% per year age, 95% CI = [0.12, 0.66], rate_lymphoid_ = 0.84% per year age, 95% CI = [0.54, 1.15] *P*_*interaction*_ = 0.0276; and rate_myeloid_ = 1.32% per year age, 95% CI = [1.00, 1.65], *P*_*interaction*_ = 1.48 × 10−^5^) (Fig. 5B). Of individuals with enriched autosomal mCAs, 5.0% of individuals with lymphoid associated mCAs went on to develop a subsequent lymphoid hematologic malignancy, while 7.3% of individuals with myeloid-associated mCAs went on to develop a myeloid malignancy.

There were 36 (6.1%) individuals with any autosomal mCA diagnosed with a subsequent hematologic malignancy and 11 (1.4%) individuals with mLOY diagnosed with a subsequent hematologic malignancy (Supplementary Table 4). Mean time to diagnosis between first measured mCA and hematologic malignancy was 11.07 (SD = 3.94) years. Only one individual with mLOX had a subsequent hematologic malignancy, so no analysis for mLOX was performed. We observed an increased, though not significant, expansion of autosomal mCAs among individuals who subsequently developed a hematologic malignancy (rate_malignancy_ = 1.16% per year age, 95% CI = [0.55, 1.76] vs. rate_no_malignancy_ = 0.72% per year age, 95% CI = [0.57, 0.87], *P*_*interaction*_ = 0.167) (Fig. 5C). When stratifying individuals with autosomal mCAs into incident lymphoid (*N* = 13) and myeloid (*N* = 23) cases, we observed a higher rate for myeloid cases (0.82% per year age, 95% CI = [0.36, 1.28], *P*_*interaction*_ = 0.939) relative to lymphoid cases (0.52% per year age, 95% CI = [−0.08, 1.13], *P*_*interaction*_ = 0.101); although the differences from those without malignancy were insignificant potentially due to small case numbers (Fig. 5D). For males with mLOY, we observed a reduced clonal expansion rate among those who subsequently developed an incident hematologic malignancy, but this reduction was not statistically significant (rate_malignancy _= 1.71% per year age, 95% CI = [0.95, 2.47] vs. rate_no_malignancy_ = 1.95% per year age, 95% CI = [1.86, 2.04], *P*_*interaction*_ = 0.126) (Fig. 5C). When stratifying by lymphoid (*N* = 6) or myeloid malignancy (*N* = 5), males with myeloid leukemia diagnoses had lower mLOY expansion rates compared to males without a subsequent hematologic malignancy (rate_malignancy_=0.98% per year age, 95% CI = [0.78, 1.89], *P*_*interaction*_ = 0.563) vs. rate_no_malignancy_ = 1.91% per year age, 95% CI = [1.83, 1.98], *P*_*interaction*_ = 0.126) (Fig. 5D); although case numbers were small potentially limiting generalizability.

## Discussion

Population-based cross-sectional studies of mCAs in circulating leukocytes suggest mCAs on average, increase in clonal fraction (CF) with increasing age^16^, but little is known about the dynamics of mCAs at the individual event level in disease-free individuals. While we observed a correlation between the rate of expansion of autosomal mCAs and cross-sectional CF, the correlation was modest, suggesting factors not captured in the cross-sectional data contribute to the expansion of mCAs.

Our large longitudinal characterization of mCAs enabled precise estimates of clonal expansion rate utilizing linear mixed models that account for individual mCA variation in both intercept and slope. Rates of clonal expansion differed by type of mCA, with mLOY having the overall highest rates of clonal expansion and mLOX having the lowest rates. The rate of mLOY mirrored that in a smaller investigation^26^. The greatest heterogeneity of expansion was observed for autosomal mCAs and was explained by different rates of clonal expansion by event copy number status and chromosomal location (e.g., range of expansion rates from − 0.20 % to 2.89 % per year of age). CN-LOH events overall had the lowest average clonal expansion rates (0.57% per year age), but a wide range of variability was observed in CN-LOH event expansion rate, accounting for both the highest (e.g., 9p and 3q LOH) and lowest (e.g., 11p and 1p LOH) mCA expansion rates. It is noteworthy that *JAK2* is located on 9p, with CN-LOH commonly occurring in myeloid malignancy cases with V617F mutations^27^. Other common chromosomal alterations observed in hematologic malignancies (e.g., 20q deletions, 12 gains) also had among the highest rates of clonal expansion. Likewise, mCAs classified as associated with myeloid malignancies had the highest rate of expansion in our models with low initial CF and high slopes, suggesting rapid expansion of these mCAs in the myeloid compartment. These observations highlight the relevance of a high clonal expansion rate for disease-relevant mCAs.

We also examined subject-level factors that could promote clonal expansion. We did not observe evidence to suggest differences in clonal expansion rate by sex or BMI category. Age was observed to interact with clonal expansion rate, with older individuals harboring autosomal mCAs having elevated expansion rates. A multitude of age-related factors could promote this higher expansion rate; although, the reduced stem cell diversity in aging individuals is a feature that merits additional investigation in relation to the clonal trajectory of CH^28^. Interestingly, males in older age groups or who were current smokers had lower observed rates of mLOY clonal expansion. This is unexpected as cross-sectional investigations indicate a higher frequency of mLOY in older males and current smokers in the general population^12^. The lower observed rate of mLOY expansion in these males could be attributable to the higher initial cell fraction of mLOY events at the start of our observational window, limiting the potential clonal expansion before a theoretical maximum threshold of mLOY cell fraction is reached. The lower expansion of mLOY in smokers could also be due to clonal competition in smokers from co-occurring types of clonal hematopoiesis, which may be competing with mLOY clones^29^. Our longitudinal investigation likewise observed individuals with autosomal mCAs and prevalent co-occurring mLOY to have higher rates of clonal expansion, suggesting potential cooperation between autosomal mCAs and mLOY; however, this increased rate was not observed for autosomal mCAs with incident co-occurring mLOY, which could indicate different dynamics with newly acquired mLOY. This potential competition of clones in individuals harboring sex chromosome loss and an autosomal event could support a potential mechanism by which additional mCAs attenuate mLOY and mLOX clone growth^24^. As additional longitudinal studies of individuals with multiple events are conducted, the expanded sample size will help disentangle interrelationships when multiple mCA clones coexist.

While the mLOX and mLOY polygenic risk scores had expected relationships with incident mCAs in the cross-sectional PLCO cohort, we did not observe a relationship between the polygenic risk scores and rate of clonal expansion, suggesting mechanisms related to genetic susceptibility to mCA development are likely distinct from those promoting clonal expansion. It is possible that specific components of the polygenic risk scores (e.g., variation in the *TCL1A* locus) are associated with the rate of clonal expansion, as the effect of the minor allele of a clonal expansion-related SNP^17^, rs1122138, had a lower expansion rate, but the difference was not statistically significant, suggesting the longitudinal sample had limited power to test individual variant associations.

The rate of autosomal mCA expansion was increased for individuals diagnosed with a subsequent hematologic malignancy, though the increase was not statistically significant. In contrast, the rate of mLOY was decreased for hematologic malignancies overall and decreased in males diagnosed with myeloid malignancies. This potential difference in rate of clonal expansion could reflect prior findings that autosomal mCAs have strong effects on incident hematologic malignancy risk, potentially driving a higher clonal expansion rate; while evidence for an association of mLOY alone with risk of a hematologic malignancy is limited^24,30,31^. Future studies which include more longitudinal samples of individuals who progress to develop a hematologic malignancy will be helpful for better deciphering which mCAs confer high clonal expansion rates, potentially leading to increased risk of hematologic malignancies and other hematologic disorders.

This population-based longitudinal characterization of mCA incidence and clonal expansion in an aging population not selected for specific disease characteristics or conditions allows for the identification of factors associated with clonal trajectory that are not readily estimated from cross-sectional or clinical investigations. We present evidence for mCA intrinsic and individual factors that alter the clonal trajectory of leukocytes harboring mCAs. Unexpected relationships in individuals with multiple detected mCAs underscores the complexities of clonal expansion in mutated hematologic progenitor cells; however, associations of elevated clonal expansion rate with hematologic malignancy risk motivates further investigation aimed at identifying CH clones with high risk of progressing to a hematologic malignancy.

This analysis was limited in the representation of mLOX, which, despite being 33% of the cross-sectional mCAs observed in this PLCO cohort, was only 14% of the mCAs investigated in the longitudinal sample. The PLCO cross-sectional cohort was mostly European ancestry (89%) as inferred by GRAF-pop, potentially limiting the generalizability of these findings across genetic ancestries. We also note that associations reported with individual risk factors provided nominal evidence for associations, and that additional larger studies are needed to amass statistically significant evidence for multiple comparison-adjusted associations of potentially weaker effect sizes. Investigating the relationship between germline genetic features, such as the mCA prevalence-related PRS and the rs1122138 SNP, did not show any statistically significant relationships with clonal expansion in these models, which could be due to the limited sample size to evaluate low-impact genetic effects. Prospective studies of serially collected blood samples in biobanks or clinical cohorts will help to increase sample size for further studies of germline genetic effects.

## Methods

### Sample Population – PLCO

The Prostate, Lung, Colorectal, and Ovarian Cancer Screening Study (PLCO) was a prospective, controlled, multisite cancer screening trial which enrolled 154,807 participants ages 55 to 74 between 1993 and 2001 from 10 screening centers in the United States to test the efficacies of preventative cancer screening approaches for the primary outcome of cancer-related deaths. The trial transitioned to a longitudinal cohort study for cancer incidence and mortality follow-up (ClinicalTrials.gov NCT00339495 -Etiologic and Early Marker Studies). For this analysis, “PLCO study” refers to the Etiologic and Early Marker Studies observational study^32^. All study participants provided written, informed consent, and the study was approved by the Institutional Review Boards at the 10 screening centers and the National Cancer Institute. The study collected biospecimens and survey data from participants. Surveys collected information on participant demographics, family history, medical history, and individual factors such as lifestyle and dietary risk factors. Blood was collected from screening arm participants at baseline and at annual screening visits for up to 5 years after the initial visit. Cancer incidence has been tracked with a median length of follow-up > 18 years and diagnoses were confirmed by medical record review or linkage to cancer registries^33,34^.

### Cross-sectional sample genotyping and PRS Calculation

Of the 117,551 PLCO study participants who gave consent for genotyping array analysis and had an available DNA source, 110,562 were scanned with one of four genotyping arrays (Illumina Global Screening Array, OncoArray, Omni2.5 M, and OmniExpress) and had genotyping of sufficient quality to be harmonized and imputed as previously described^35^. In brief, genotyping was performed at the NCI Division of Cancer Epidemiology and Genetics Cancer Genomics Research (CGR) Laboratory according to manufacturer protocols and by internal standard operating procedures. Quality control filtering removed subjects with failed output files, completion rate < 0.95, contamination > 0.10, and replicates with genotype concordance > 0.95 for a set of LD-pruned SNPs. Samples that failed a sex concordance check based on X chromosome method-of-moments F coefficient from PLINK were further screened for sex chromosome aneuploidies by STR profiling using AmpFLSTR Identifier assay and sex discordant individuals were excluded. Relatedness was screened using PLINK IBS/IBD test with pi_hat threshold of 0.1 for removal of related individuals.

Genotype imputation was performed using the TOPMed reference panel on the Michigan Imputation Server. Variants were filtered based on minor allele frequency ≤ 0.01, variant-level missingness ≥ 0.05, and with the Hardy-Weinberg equilibrium exact test *P* ≤ 1.0 × 10−^6^ prior to imputation. The data from each genotyping platform were aligned to reference datasets (HRC-1000G-check-bim.pl) using a community-recommended script modified to support TOPMed 5b as a reference panel using a pre-existing test imputation with 1000 Genomes Project subjects compared to the TOPMed 5b reference panel. Data were lifted over from GRCh37/hg19 to GRCh38/h38 using MIS. Pre-phasing used phase reference data from TOPMed release 5b on EAGLE 2.4 with imputation against the same reference panel using minimac4. Raw imputation data were partitioned into subsets based on genetic ancestry estimation using GRAF-pop^36^. Prior to imputation, “African” and “African American”, and “East Asian” and “Other Asian” were combined into “African American (combined)” and “East Asian (combined)”. Each platform and ancestry pair were cleaned according to the method from Kowalski et. al.^37^ to remove variants with an Rsq < 0.3.”

Polygenic risk scores (PRS) were calculated based on independent signals associated with the outcome of interest, and allelic dosage of the effect alleles were weighted by the association beta values reported for mLOY (Thompson et al.^13^), mLOX (Liu et al.^14^), and telomere length (Codd et al.^38^) for all PLCO study participants whose genotyping array data was imputed based on European Ancestry (*n* = 100,448)^35^. Scores were mean standardized across those participants, and four quartiles of risk for each PRS were defined based on ranking individuals by score and separating into four even groups. Increasing quartiles of mLOX and mLOY PRS scores correspond to a greater risk of prevalent mLOX or mLOY, respectively. Increasing quartiles of telomere length PRS score correspond to longer genetically estimated telomere lengths.

### Longitudinal sample selection and genotyping

For the longitudinal mCA analyses, genotyping of additional samples was performed for a subset of individuals from the 10,747 individuals with mCAs from the cross-sectional analysis (Table 1). The analysis workflow for selection is described in detail in Supplementary Fig. 2C.

The PLCO longitudinal samples were genotyped using two Illumina arrays: the GSAMD-24v1 array (GSAMD-24v1-0_20011747_A1.bpm; GRCh37) for the initial time points and the GSAv3Confluence array (GSAv3Confluence_20032937 × 371431_A1.bpm; GRCh38) for additional time points. Samples genotyped on the GSAMD-24v1 array were converted to GRCh38 by realigning SNP flank sequences from the existing GRCh37 manifest files to the GRCh38 reference genome using the BWA aligner and recomputing SNP positions, following the procedure described in the gtc2vcf documentation (https://github.com/freeseek/gtc2vcf).

### MoChA analysis for mCA detection

MoChA, which uses a Viterbi hidden Markov model with multiple alternate states to detect chromosomal aberrations as phase B-allele frequency deviations (https://github.com/freeseek/mocha), was applied to detect mCAs from the genotyping array data. Prior to running MoChA, the genotyping intensity data were phased using SHAPEIT4 with the 1000 Genomes Phase 3 reference panel (1kGP_high_coverage_Illumina). The phased BAF autocorrelation (baf_auto) was used to detect and estimate sample contamination. A sample call rate threshold of ≥ 0.97 and a phased BAF auto correlation (baf_auto) ≤ 0.03 were applied, based on the MoChA developers’ recommendations. All analyses were based on the GRCh38 genome build. To calculate the proportion of cells with a specific type of mosaic event (clonal fraction), the B deviation (Bdev, deviation from the expected BAF value of 0.5 for heterozygous SNPs) was used: Loss: CF = 2*Bdev / (0.5 + Bdev); Gain: CF = 2*Bdev / (0.5-Bdev); Neutral: CF = 2*Bdev. Detection of mLOY was called using changes in phased-BAF within the pseudo-autosomal region (PAR1) on the X chromosome for the GSA, Omni25M, and OmniExpress arrays. Detection of mLOY on Oncoarray was based on the median of log R ratio signal ≤ − 0.15 across the male-specific region of the Y chromosome, due to the low number of probes within the PAR1 region^15,39^. Because of this limitation, mLOY events detected from Oncoarray were removed from analyses of clonal fraction (CF) in the cross-sectional cohort. Of the 110,562 PLCO participants whose genotyping passed quality control for MoChA analysis, 56,324 had DNA collected from a blood sample and were considered for further analysis.

### Statistical analysis

Comparisons of cohort characteristics and mCA characteristics utilized independent two-sample Student’s T-Tests and Chi-squared tests as appropriate, with *P-values* reported in-text.

To comprehensively characterize the longitudinal trajectories of CF as a participant ages, we fit LMMs. Using this method, we can examine average changes in CF with age and how the longitudinal trajectories vary by demographic features. In addition, we can assess the variability of individual trajectories in the population as well as optimally estimate individual-level trajectories. Let *Y*_*ijc*_ be the CF for the *c*^*th*^ mCA, *j*^*th*^ timepoint and the *i*^*th*^ participant. We fit the model:

$${Y}_{{ijc}}={\beta }_{0}+\,{\beta }_{1}{t}_{{ij}}+\,{\beta }_{2}({{\mathrm{var}}})+{\beta }_{3}({t}_{{ij}}*{{\mathrm{var}}})+{b}_{i0}+{b}_{i1}{t}_{{ij}}+{b}_{{ci}}\,+\,{\varepsilon }_{{ijc}}$$

where *β*_*0*_, *β*_*1*_, *β*_*2*_, and *β*_*3*_ characterize population averaged changes and the effect of the tested covariate (*var*) (e.g., sex, BMI, smoking status) on these changes over time. The random effects *b*_*i*_ and *b*_*i1*_ describe individual variation in the intercept and slope for the trajectory. The random effects *b*_*ci*_ denote variation between mCA nested within an individual. The random effect *b*_*ci*_ is only included for autosomal mCAs, as individuals cannot have multiple mLOX or mLOY events. Age at collection was scaled to 65 years of age for LMMs by subtracting 65 from the age at sample collection. T-Tests with Satterthwaite’s degrees of freedom were used to test the significance of the interaction term (*P*_*interaction*_, null hypothesis (H_0_): *β*_*3*_ = 0, alternative hypothesis (H_A_): *β*_*3*_ ≠ 0) using the lmerTest R package. Graphs of average fixed effect profiles are based on the ggeffects R package with confidence intervals based on the standard errors assuming a normal distribution (+/− 1.96 * SE). Statistical analyses were performed with R version 4.2.1. For linear mixed models, only the 1746 mCAs with multiple observations across timepoints (Table 2) were considered. Body mass index (BMI) and smoking status were defined based on survey responses closest to the collection of the earliest blood sample. Individuals with missing covariate data were excluded from analyses for that covariate.

All reported *P-values* are not adjusted for multiple comparisons with *P* ≤ 0.0055 meeting the Benjamini-Hochberg False Discovery Rate (FDR) multiple comparison threshold (*q* = 0.05) for 115 analysis-wide tests of significance. Tests from multivariable models were not included in the analysis-wide number of tests.

### Classification of hematologic malignancies

Enriched autosomal mCAs were classified based on Niroula et al.^25^ A_mCAs (ambiguous) and U_mCAs (unclassified) were grouped as “non-specific” for cell lineage (Supplementary Table 3). For hematologic malignancies diagnosed in the PLCO longitudinal sample, ICD-O-3 codes were used to classify lymphoid or myeloid disease (Supplementary Table 4).

### Reporting summary

Further information on research design is available in the Nature Portfolio Reporting Summary linked to this article.

## Supplementary information


Supplementary Information File
Reporting Summary
Transparent Peer Review file


## Source data


Source Data


## Data Availability

Genotyping array data are available on the public repository dbGaP under the study accession: phs001286.v5.p2. PLCO study allows investigators to use study data for discovery and hypothesis generation in the investigation of genetic contributions to cancer, adult diseases and novel GWAS methods. To protect research participants, controlled access to PLCO companion phenotype data is provided only to researchers who, along with their institutions, agree to the data access terms described through the PLCO Cancer Data Access System (CDAS). CDAS maintains the most current information regarding the expected timeframe for access requests and data delivery. Projects are reviewed and approved by CDAS staff and the NCI Data Access Committee within 1 to 4 weeks of data-only request submissions. Access to data is granted for 12 months, at which time extensions for access can be requested. The analytic pipeline used for MoChA analysis is publicly available on GitHub at mocha.wdl. Source data are provided in this paper.
