# Supplementary material for: Longitudinal characterization of mosaic chromosomal alterations identifies factors influencing clonal dynamics of leukocytes
Source: Nat Commun. 2026 Jul 29;17:9192. doi: 10.1038/s41467-026-75996-5 (PMC13522525; doi:10.1038/s41467-026-75996-5)
Supplement: Supplementary file 1 [file 41467_2026_75996_MOESM1_ESM.pdf]

Longitudinal characterization of mosaic chromosomal alterations identifies factors influencing clonal dynamics of leukocytes

Supplementary Information

Supplementary Figure 1. Population clonal fraction of events observed in the cross-sectional PLCO cohort

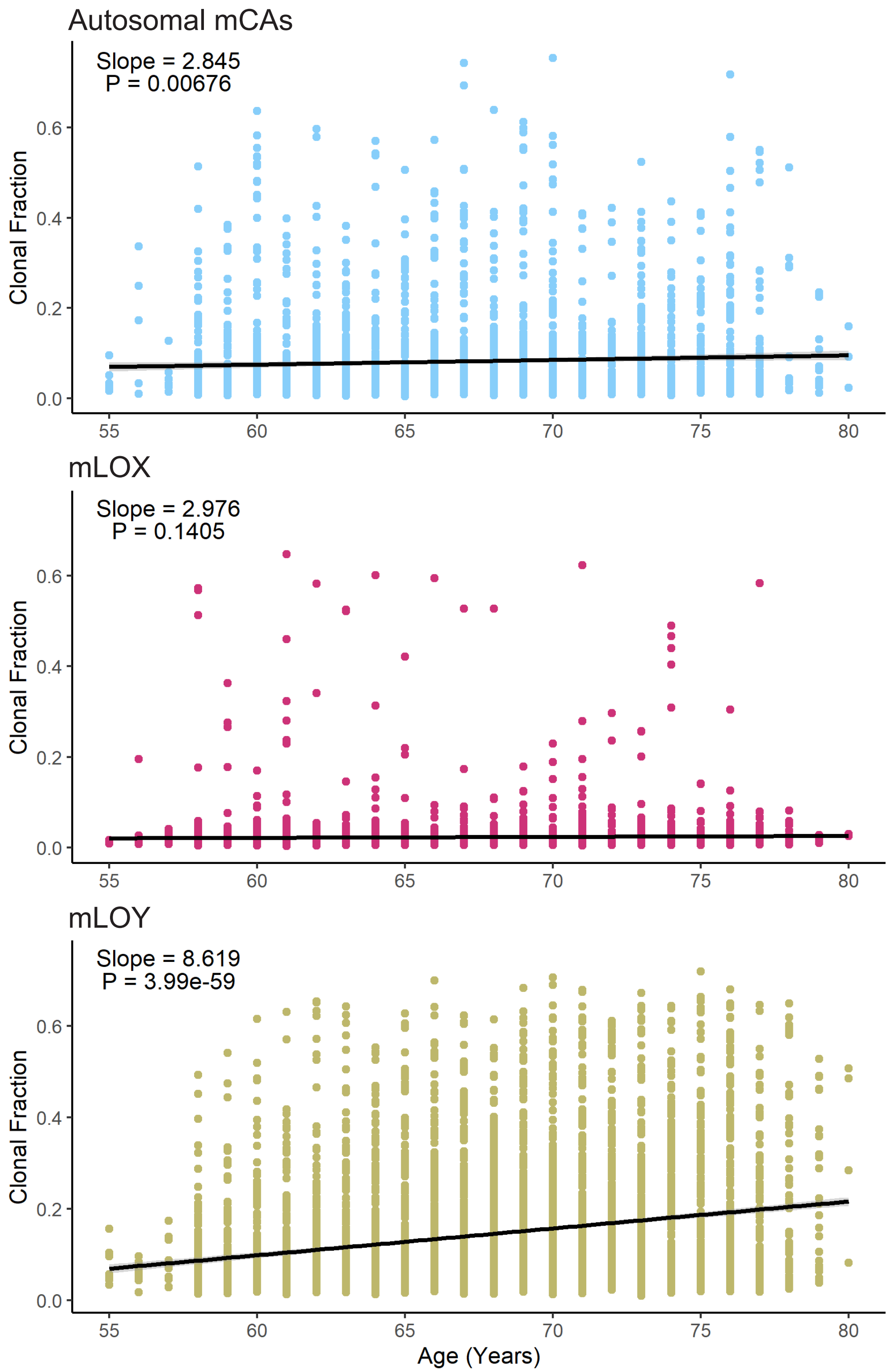

Scatter plots and slopes from simple linear regression of CF and age at sample collection are shown for the three most abundant mCA types observed in the cross-sectional cohort (autosomal mCAs – top; mLOX – middle; mLOY – bottom). Slope is the beta-coefficient for the simple linear regression model with clonal fraction as the outcome and age as the predictor. P-Value (P) is from a two-sided, independent T-Test. Source data are provided as a source data file.

Supplementary Figure 2. Distributions of autosomal mCAs by clonal fraction in cross-sectional PLCO cohort

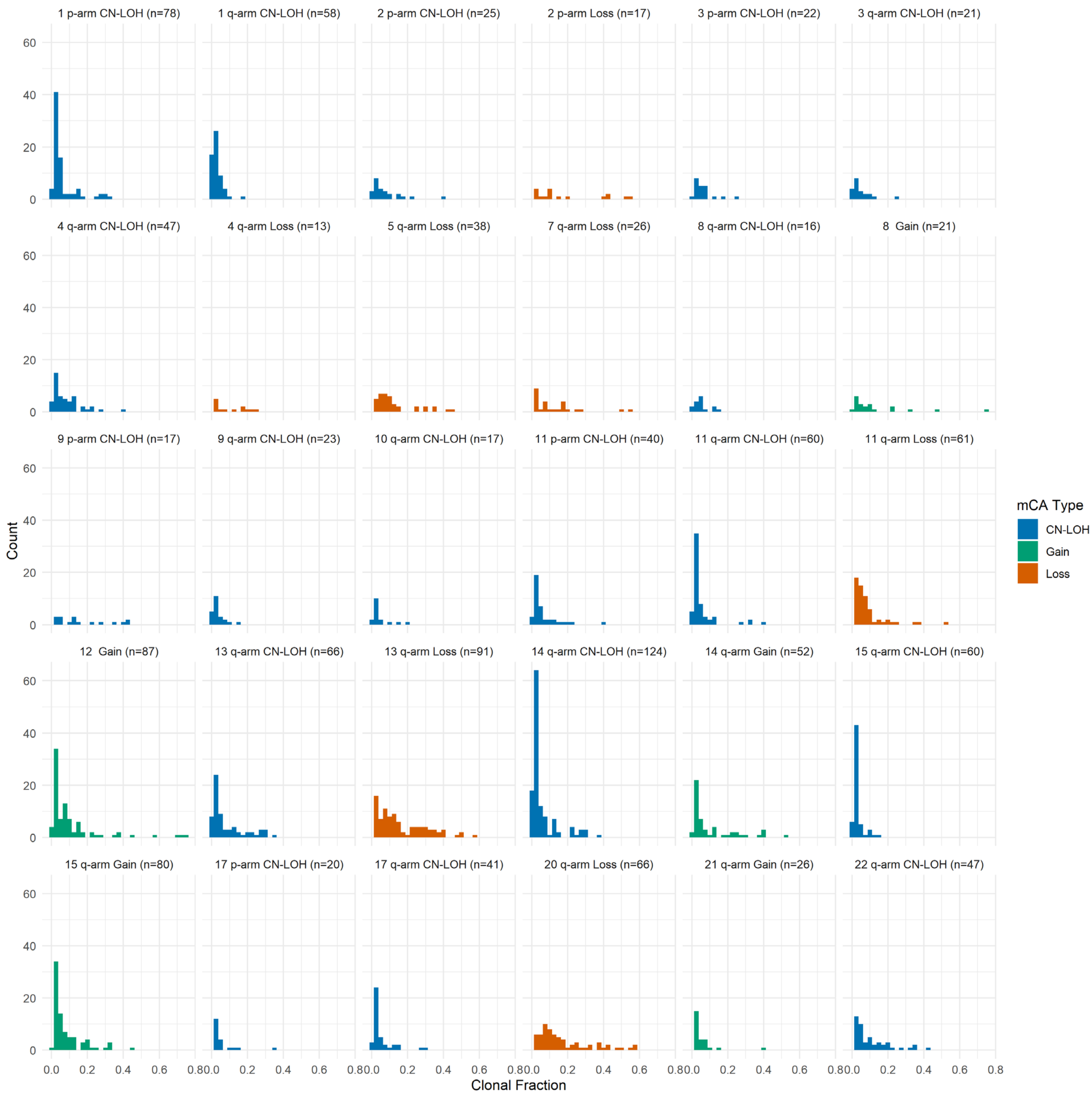

Histograms of clonal fraction of 30 enriched autosomal mCAs (chromosome-arm-type) detected in 1,246 individuals in the cross-sectional PLCO cohort.
Source data are provided as a source data file.

Supplementary Figure 3. Study workflow and samples and mCAs per individual

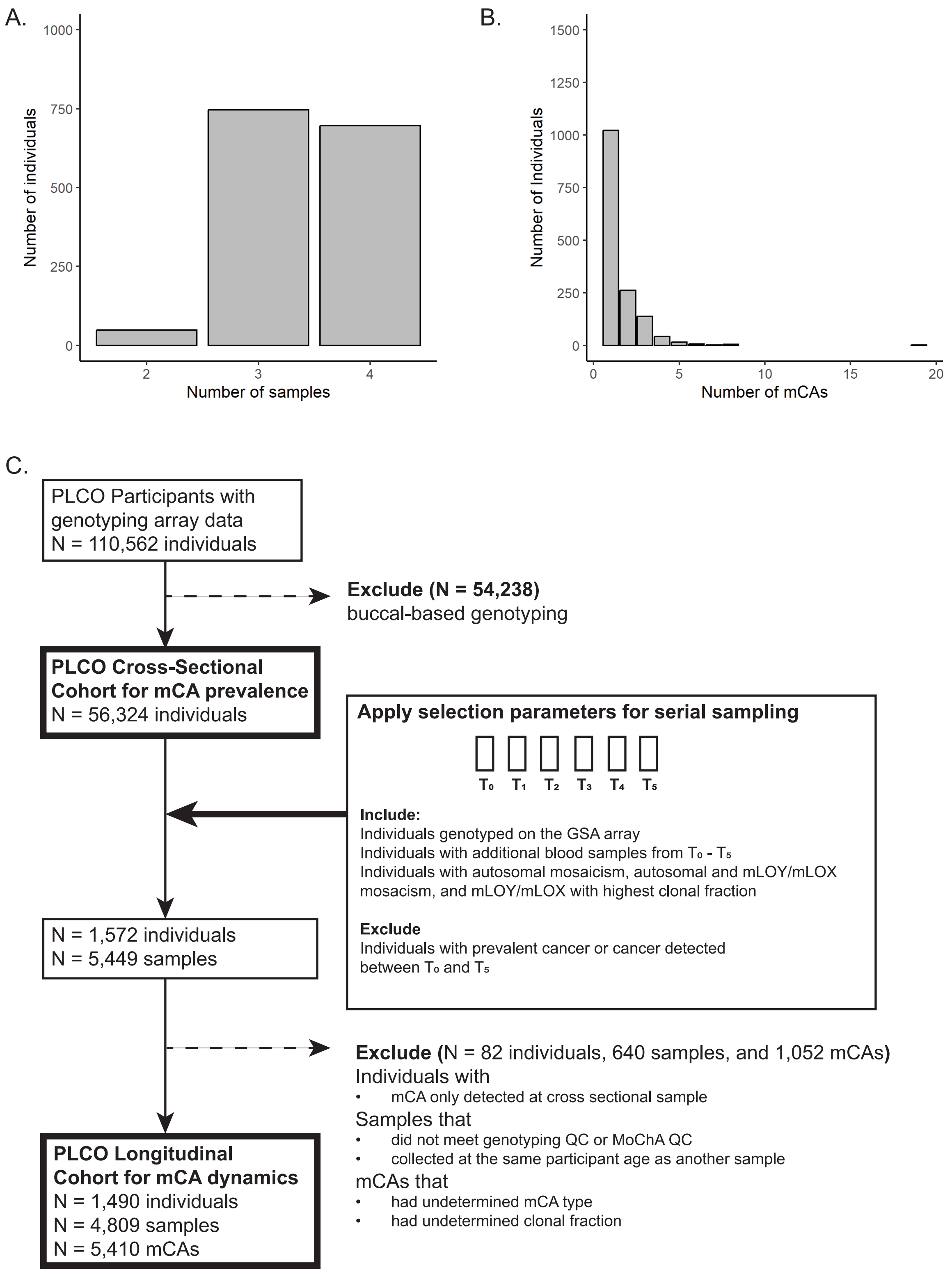

A. Bar graph of the number of samples per individual in the longitudinal serial-sample cohort.
B. Bar graph of number of mCAs per individual participant in the longitudinal serial sample cohort.
C. A graphical depiction of the PLCO blood-based genotyping cross-sectional study and the selection criteria and exclusions for the longitudinal cohort.
Source data are provided as a source data file.

Supplementary Figure 4. Enriched longitudinal autosomal mCAs

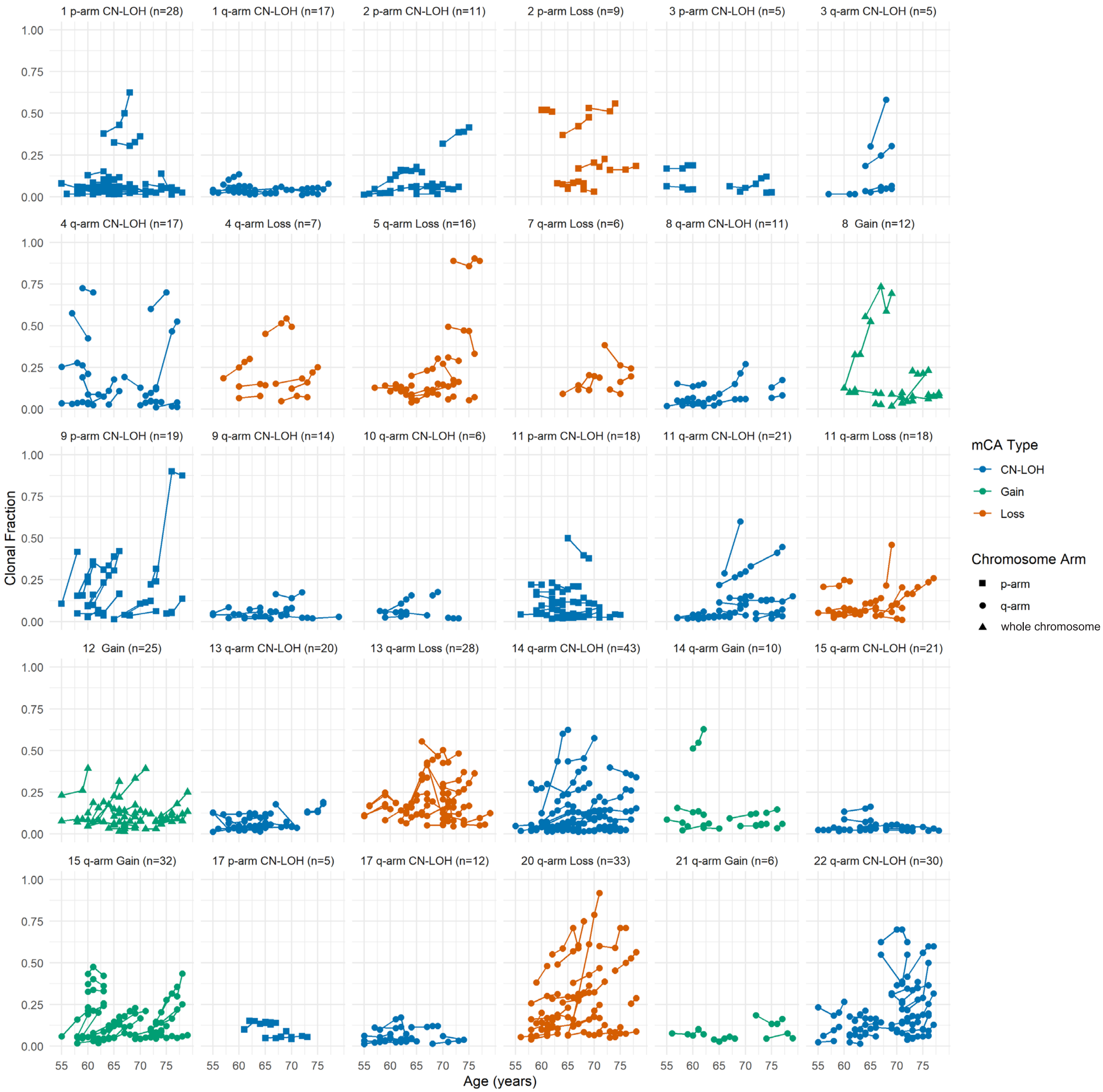

Line plots of CF across age for 30 enriched longitudinal autosomal mCAs (chromosome-arm-type) detected in 450 individuals in the longitudinal serial-sample cohort.
Source data are provided as a source data file.

Supplementary Figure 5. Expansion of mCAs by sex and BMI

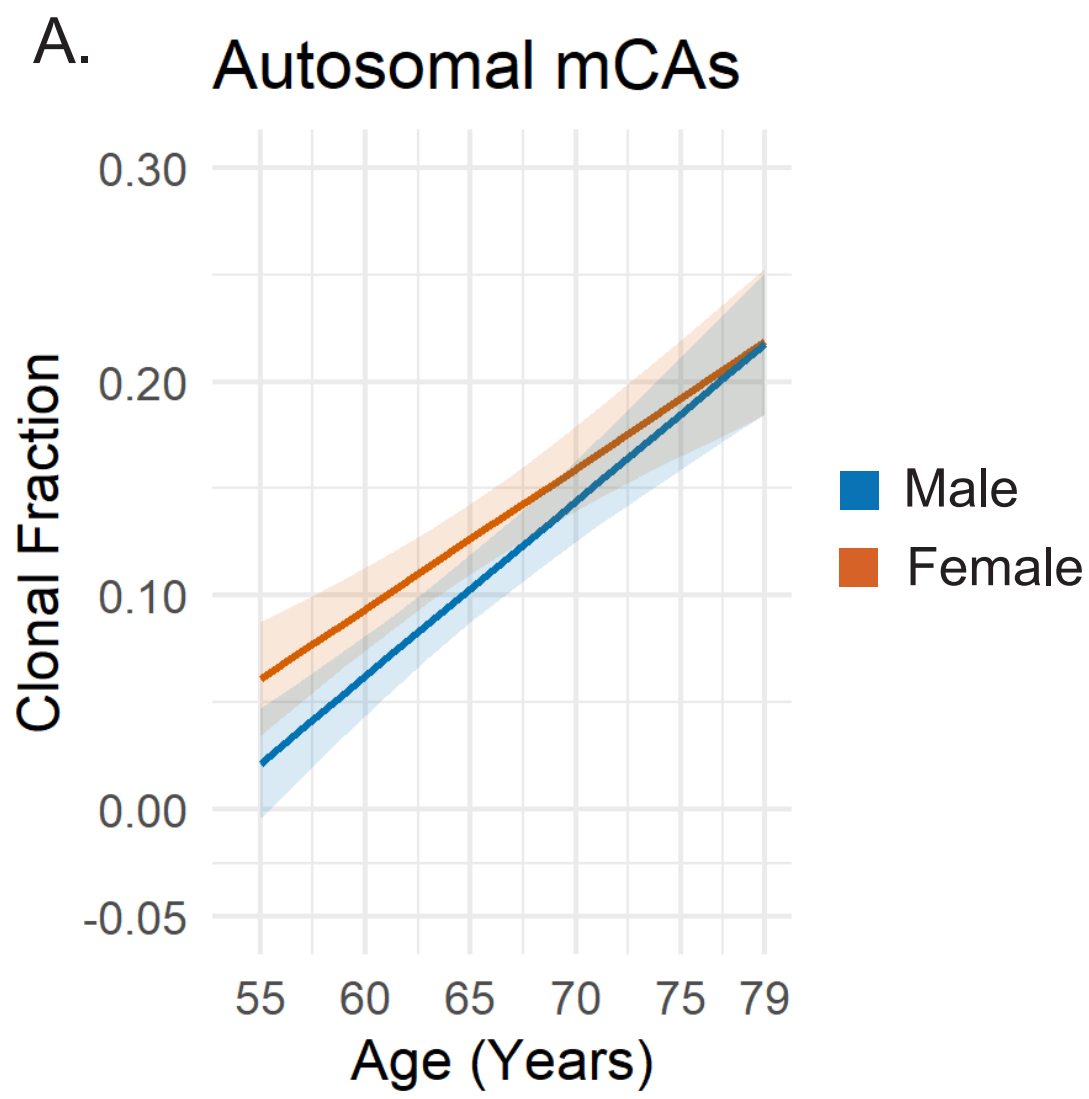

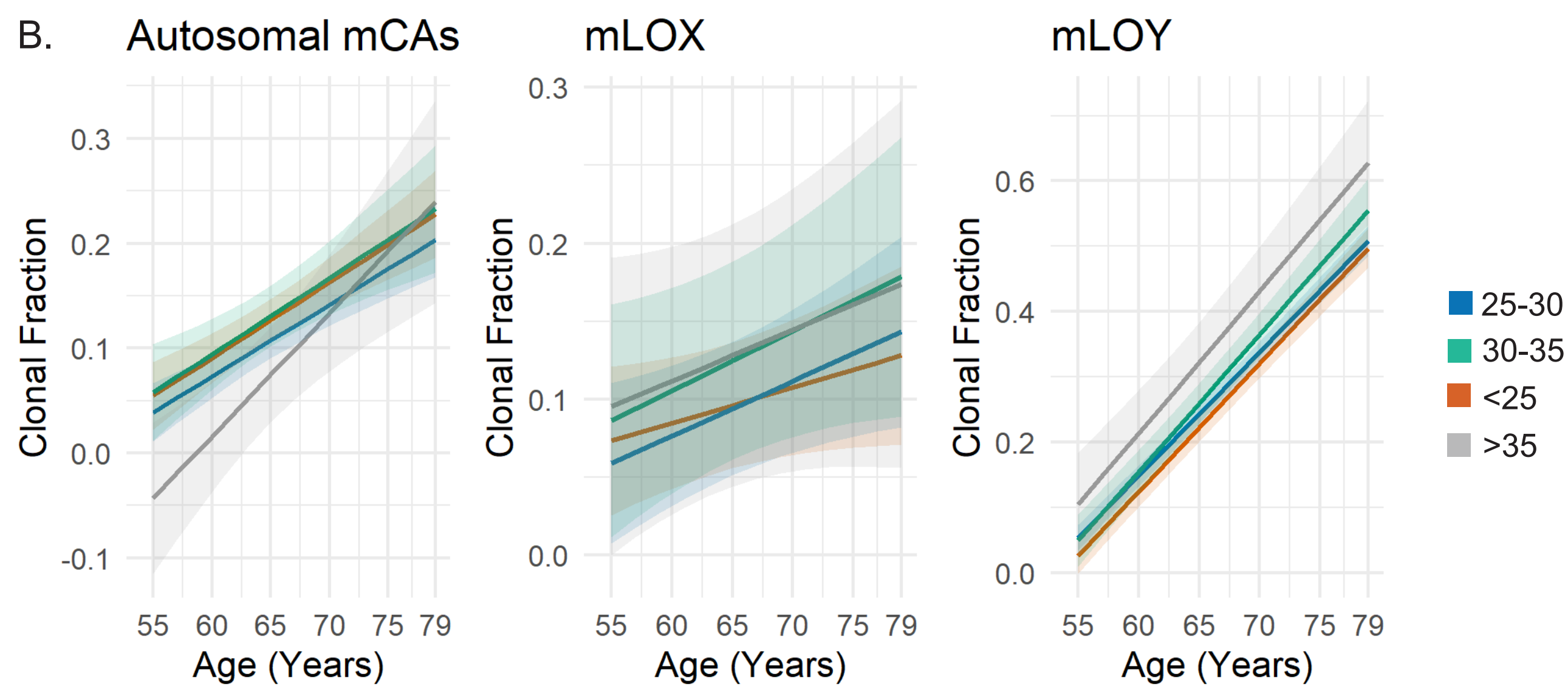

A. Profile plots showing the effect of sex on autosomal mCA expansion across age (female N=287 (orange), male N=306 (blue). The shaded bands around the average slopes indicate the 95% CI of the mean trajectories. **B.** Profile plots showing the effect of BMI category on clonal expansion across age. Autosomal mCAs: BMI <25 N=234 (orange), BMI 25-30 N=330 (blue), BMI 30-35 N=110 (green), BMI >35 N=44 (gray); mLOX BMI<25 N=74 (orange), BMI 25-30 N=66 (blue), 30-35 N=30 (green); BMI >35 N=17(gray); mLOY: BMI <25 N=234 (orange), BMI 25-30 N = 428 (blue), BMI 30-35 N=101 (green), BMI >35, N=25(gray). The shaded bands around the average slopes indicate the 95% CI of the mean trajectories. Source data are provided as a source data file.

Supplementary Figure 6. Clonal expansion of mLOY stratified by starting clonal fraction

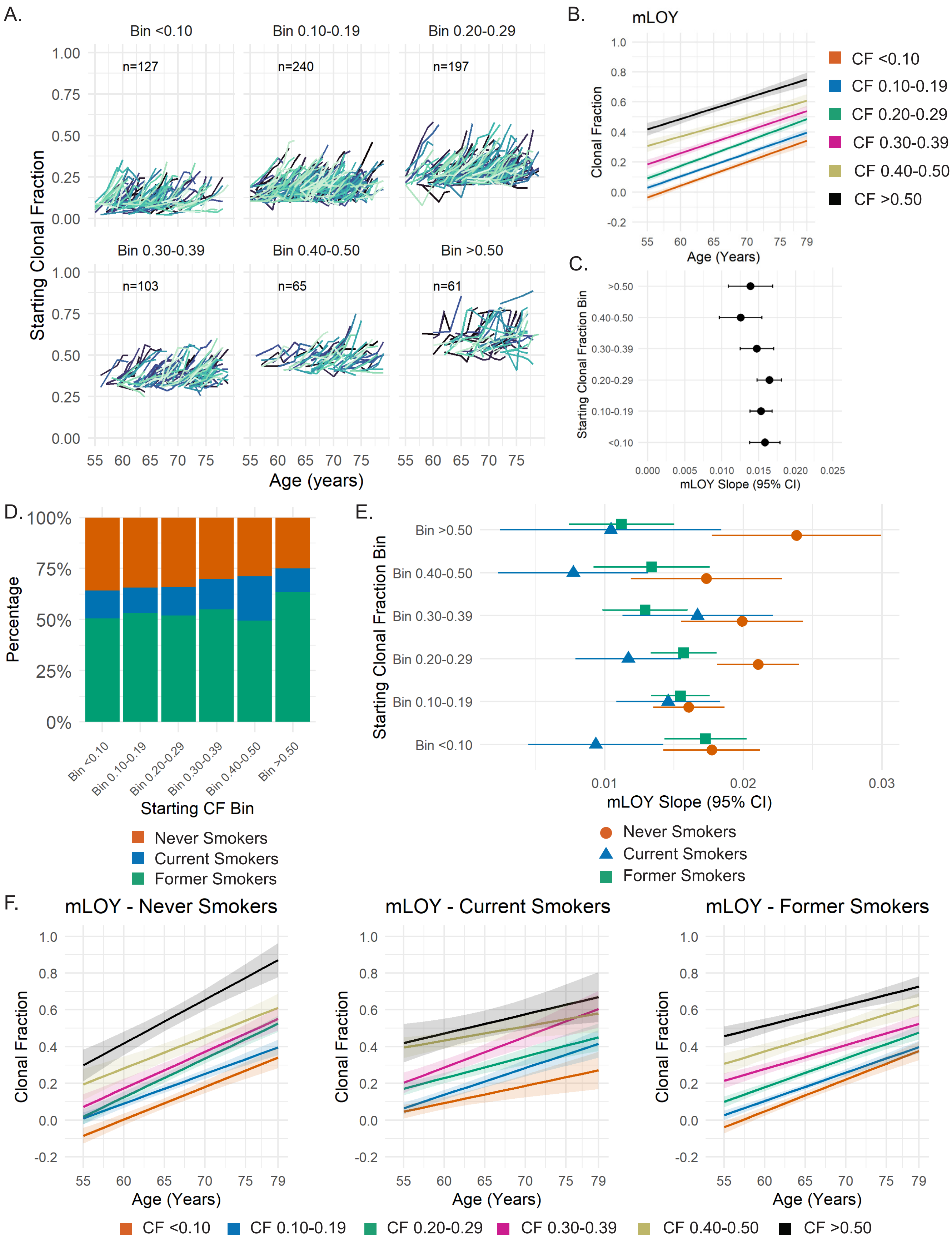

A. Line plots of individual mLOY trajectories separated by six bins based on the clonal fraction of an individual's first measurement of mLOY. **B.** Profile plot of population average mLOY clonal expansion by starting clonal fraction bin (CF <0.10 N=127 (orange), CF 0.10-0.19 N=240 (blue), CF 0.20-0.29 N=197 (green), CF 0.30-0.39 N=103 (magenta), CF 0.4-0.50 N=65 (yellow), CF >0.50 N=61 (black). The shaded bands around the average slopes indicate the 95% CI of the mean trajectories.
C. Forest plot of slopes (expansion rate) from LMMs of mLOY clonal expansion across age for each starting clonal fraction bin. **D.** Percentage of individuals with each smoking status within the starting clonal fraction bins. **E.** Forest plot of slopes and 95% confidence intervals from LMMs of mLOY clonal expansion stratified by smoking status. **F.** Profile plots of population average mLOY clonal expansion by starting clonal fraction bin stratified by smoking status. Never smokers: (CF <0.10 N=47 (orange), CF 0.10-0.19 N=81 (blue), CF 0.20-0.29 N=67 (green), CF 0.30-0.39 N=30 (magenta), CF 0.4-0.50 N=20 (yellow), CF >0.50 N=15 (black); Current smokers: (CF <0.10 N=17 (orange), CF 0.10-0.19 N=30 (blue), CF 0.20-0.29 N=28 (green), CF 0.30-0.39 N=16 (magenta), CF 0.4-0.50 N=14 (yellow), CF >0.50 N=7 (black); Former smokers: (CF <0.10 N=63 (orange), CF 0.10-0.19 N=129 (blue), CF 0.20-0.29 N=102 (green), CF 0.30-0.39 N=57 (magenta), CF 0.4-0.50 N=31 (yellow), CF >0.50 N=39 (black). The shaded bands around the average slopes indicate the 95% CI of the mean trajectories. Abbreviations: CF = clonal fraction
Source data are provided as a source data file.

Supplementary Figure 7. Polygenic risk quartile association with cross-sectional mCA prevalence

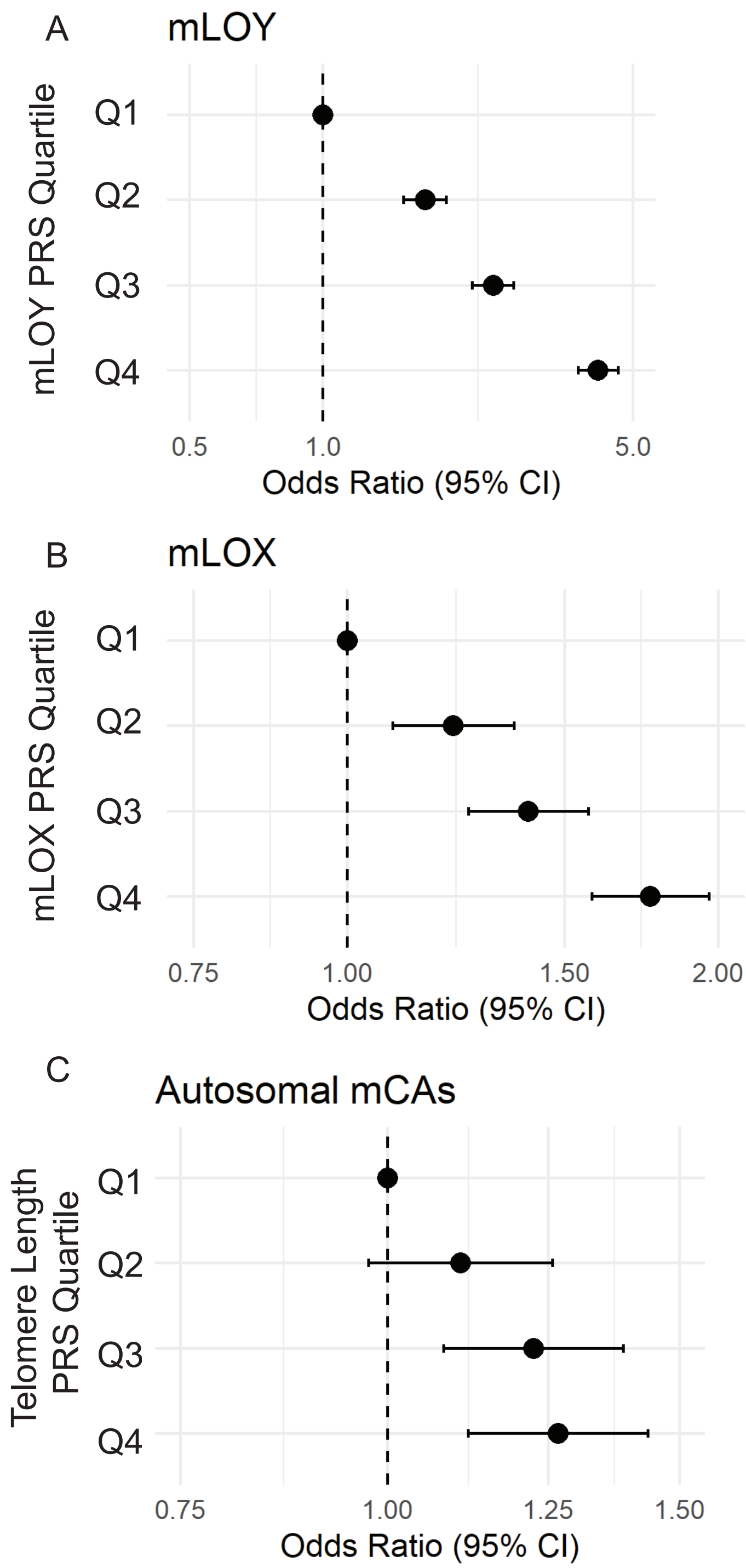

The cross-sectional associations of mCAs and PLCO population normalized PRS scores were determined from logistic regression models with mCA status as a dichotomous outcome. **A.** Measured mLOY and polygenic risk of mLOY by normalized PRS quartiles based on male PLCO participants: Quartile 1 N=6,044, Quartile 2 N=6,018, Quartile 3 N=5,964, Quartile 4 N=5,948. **B.** Measured mLOX and polygenic risk of mLOX by normalized PRS quartiles based on female PLCO participants: Quartile 1 N=5,974, Quartile 2 N=6,317, Quartile 3 N=6,145, Quartile 4 N=6,016. **C.** Measured autosomal mCAs and normalized PRS quartile of telomere length PRS: Quartile 1 N=12,219, Quartile 2 N=12,153, Quartile 3 N=12,104, Quartile 4 N=11,950. Models were adjusted for age, age-squared, smoking status, BMI, GRAFpop ancestry percentages, and genotyping array. Source data are provided as a source data file.

Supplementary Figure 8. Genetic risk and clonal expansion of mCAs across age

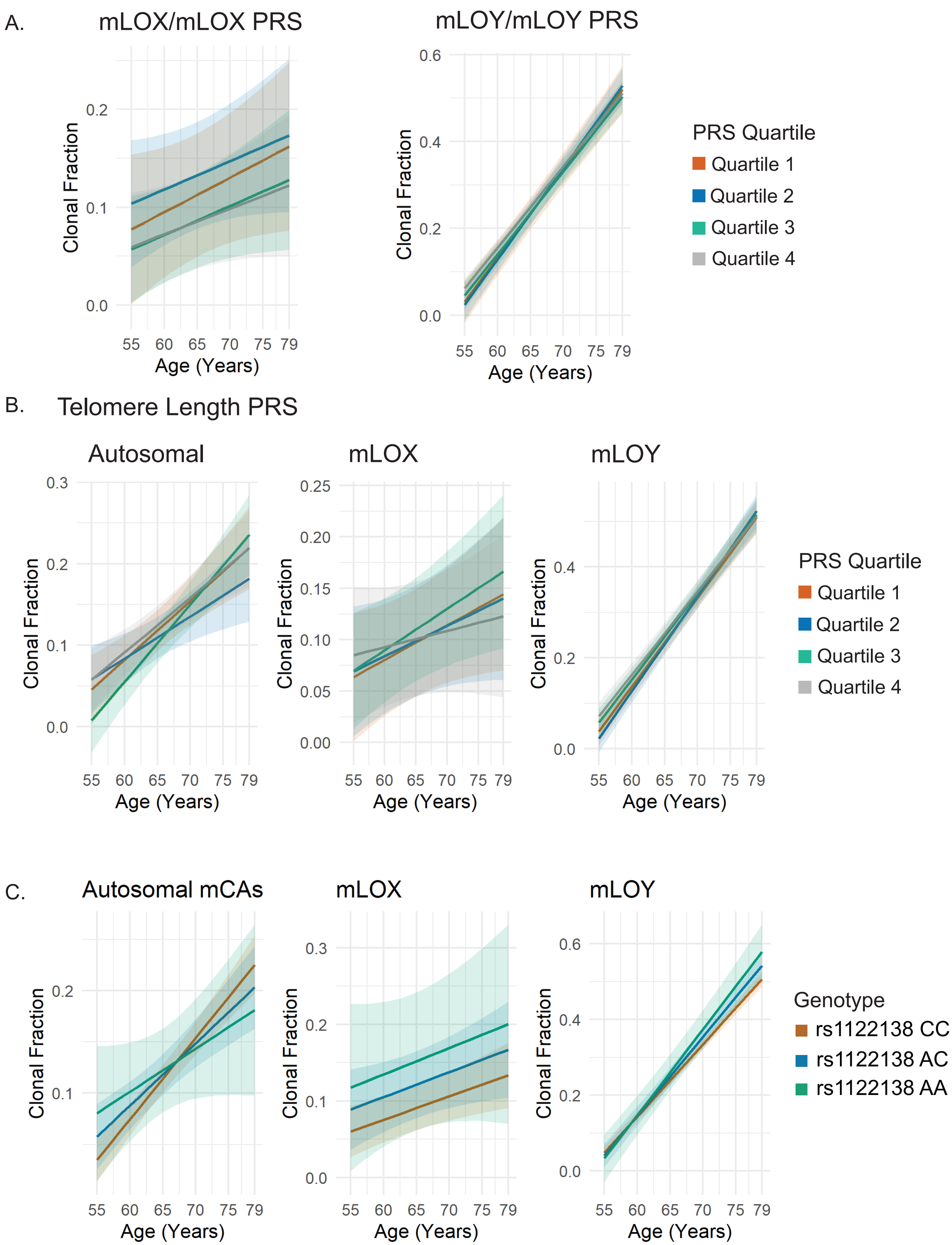

A. Profile plot for measured mLOY and polygenic risk of mLOY by normalized PRS quartiles (left panel): Quartile 1 N=75, Quartile 2 N=150, Quartile 3 N=176, Quartile 4 N=326. The shaded bands around the average slopes indicate the 95% CI of the mean trajectory. Profile plot for measured mLOX and polygenic risk of mLOX by normalized PRS quartiles (right panel): Quartile 1 N=32, Quartile 2 N=41, Quartile 3 N=52, Quartile 4 N=51. **B**. Profile plot of population average effect of normalized PRS quartile of telomere length PRS on clonal expansion. Autosomal mCAs: Quartile 1 N=166, Quartile 2 N=148, Quartile 3 N=169, Quartile 4 N=182; mLOX: Quartile 1 N=46, Quartile 2 N=42, Quartile 3 N=48, Quartile 4N=40); mLOY: Quartile 1 N=198, Quartile 2 N=182, Quartile 3 N=179, Quartile 4 N=168. Quartile 1=orange, Quartile 2=blue, Quartile 3=green, Quartile 4=gray. The shaded bands around the average slopes indicate the 95% CI of the mean trajectories. **C.** Profile plot of the population average effect of rs1122138 genotype modeled as an additive effect on expansion of mCAs. Autosomal mCAs: CC=520, CA=181, AA=16; mLOX: CC=135, CA=50, AA=3; mLOY: CC=623, CA=159, AA=6). The shaded bands around the average slopes indicate the 95% CI of the mean trajectories. Source data are provided as a source data file.

Supplementary Table 1. Summary of clonal fraction changes of the three most abundant mCA types

mCA Type	Median Change in CF	IQR	Positive change in CF
Autosomal	0.0134	-0.0015, 0.0454	527 (72.2 %)
mLOX	0.0070	-0.0040, 0.0296	124 (65.6 %)
mLOY	0.0939	0.0460, 0.1394	740 (93.3 %)

CF = clonal fraction

Supplementary Table 2. Beta coefficient for the adjusted effects of individual factors on clonal expansion and interaction with time from linear mixed models

Model	Beta Coefficient	Standard Error	Lower 95% CI	Upper 95% CI	P-Interaction
Autosomal less than 65	0.0059	0.0021	0.0018	0.0099	(ref)
Autosomal 65 or older	0.0096	0.0021	0.0055	0.0137	**0.0381**
Autosomal Female	0.0059	0.0021	0.0018	0.0099	(ref)
Autosomal Male	0.0072	0.0024	0.0026	0.0119	0.4795
Autosomal Never Smoker	0.0059	0.0021	0.0018	0.0099	(ref)
Autosomal Current Smoker	0.0058	0.0031	-0.0003	0.0120	0.9875
Autosomal Former Smoker	0.0074	0.0023	0.0029	0.0120	0.4252
Autosomal BMI<25	0.0059	0.0021	0.0018	0.0099	(ref)
Autosomal BMI 25-30	0.0053	0.0021	0.0012	0.0094	0.7826
Autosomal BMI 30-35	0.0060	0.0026	0.0008	0.0112	0.9717
Autosomal BMI >35	0.0100	0.0038	0.0026	0.0174	0.2925
mLOX less than 65	0.0038	0.0022	-0.0005	0.0080	(ref)
mLOX 65 or older	0.0016	0.0020	-0.0023	0.0055	0.2885
mLOX Never Smoker	0.0038	0.0022	-0.0005	0.0080	(ref)
mLOX Current Smoker	0.0014	0.0032	-0.0049	0.0076	0.4604
mLOX Former Smoker	0.0072	0.0024	0.0024	0.0119	0.1293
mLOX BMI <25	0.0038	0.0022	-0.0005	0.0080	(ref)
mLOX BMI 25-30	0.0047	0.0024	0.0001	0.0093	0.6900
mLOX BMI 30-35	0.0060	0.0030	0.0001	0.0118	0.4526
mLOX BMI >35	0.0039	0.0034	-0.0028	0.0107	0.9557
mLOY less than 65	0.0253	0.0011	0.0232	0.0275	(ref)
mLOY 65 or older	0.0220	0.0010	0.0201	0.0240	**0.0024**
mLOY Never Smoker	0.0253	0.0011	0.0232	0.0275	(ref)
mLOY Current Smoker	0.0185	0.0014	0.0157	0.0213	**3.44E-05**
mLOY Former Smoker	0.0222	0.0009	0.0204	0.0240	**0.0063**
mLOY BMI <25	0.0259	0.0014	0.0232	0.0286	(ref)
mLOY BMI 25-30	0.0246	0.0012	0.0223	0.0269	0.2871
mLOY BMI 30-35	0.0271	0.0018	0.0236	0.0305	0.4995
mLOY BMI >35	0.0277	0.0030	0.0218	0.0335	0.5398
mLOY less than 65	0.0253	0.0015	0.0223	0.0283	(ref)
mLOY 65 or older	0.0223	0.0016	0.0192	0.0254	**0.00556**
mLOY Never Smoker	0.0253	0.0015	0.0223	0.0283	(ref)
mLOY Current Smoker	0.0186	0.0018	0.0151	0.0222	**6.75E-05**
mLOY Former Smoker	0.0222	0.0014	0.0194	0.0251	**0.00821**
mLOY CF <0.10	0.0253	0.0015	0.0223	0.0283	(ref)
mLOY CF 0.10-0.19	0.0252	0.0013	0.0227	0.0278	0.96414
mLOY CF 0.20-0.29	0.0266	0.0015	0.0238	0.0295	0.42604
mLOY CF 0.30-0.39	0.0258	0.0018	0.0222	0.0294	0.80732
mLOY CF 0.40-0.50	0.0225	0.0021	0.0183	0.0267	0.20637
mLOY CF >0.50	0.0220	0.0022	0.0177	0.0263	0.14186

Bold font indicates nominal *P-Value* ($P \leq 0.05$) and outlined box indicates *P-Value* ≤ FDR threshold ($P \leq 0.0055$)

CF = Clonal Fraction

Supplementary Table 3. Enriched autosomal mCAs of the longitudinal cohort and their classification of association with hematologic malignancies

Classification of mCAs based on associations with hemaologic malignancy diagnosis as described in Niroula et al.[25]

Autosomal mCA	Individuals (N)	Niroula mCA/LOH type	Final_Type
10 q-arm CN-LOH	6	U-mCA	non-specific
11 p-arm CN-LOH	18	U-mCA	non-specific
11 q-arm CN-LOH	21	A-mCA	non-specific
13 q-arm CN-LOH	20	L-mCA	lymphoid
14 q-arm CN-LOH	43	M-mCA	myeloid
15 q-arm CN-LOH	21	U-mCA	non-specific
17 p-arm CN-LOH	5	A-mCA	non-specific
17 q-arm CN-LOH	12	U-mCA	non-specific
1 p-arm CN-LOH	28	A-mCA	non-specific
1 q-arm CN-LOH	17	L-mCA	lymphoid
22 q-arm CN-LOH	30	M-mCA	myeloid
2 p-arm CN-LOH	11	U-mCA	non-specific
3 p-arm CN-LOH	5	U-mCA	non-specific
3 q-arm CN-LOH	5	N/A	non-specific
4 q-arm CN-LOH	17	U-mCA	non-specific
8 q-arm CN-LOH	11	N/A	non-specific
9 p-arm CN-LOH	19	M-mCA	myeloid
9 q-arm CN-LOH	14	L-mCA	lymphoid
12 Gain	25	L-mCA	lymphoid
14 q-arm Gain	10	U-mCA	non-specific
15 q-arm Gain	32	L-mCA	lymphoid
21 q-arm Gain	6	A-mCA	non-specific
8 Gain	12	M-mCA	myeloid
11 q-arm Loss	18	L-mCA	lymphoid
13 q-arm Loss	28	L-mCA	lymphoid
20 q-arm Loss	33	M-mCA	myeloid
2 p-arm Loss	9	U-mCA	non-specific
4 q-arm Loss	7	U-mCA	non-specific
5 q-arm Loss	16	M-mCA	myeloid
7 q-arm Loss	6	L-mCA	lymphoid

Supplementary Table 4. Hematologic malignancies subsequently diagnosed in the longitudinal cohort

Number of hematologic malignancies diagnosed in individuals with longitudinal mCAs by mCA type

mCA Type	No Malignancy (N)	Hematologic Malignancies (N)	Lymphoid (N)	Myeloid (N)	Total Individuals (N)
Autosomal	557	36	13	23	593
mLOX	188	1	1	0	189
mLOY	782	11	6	5	793

ICD-O-3 codes and corresponding histology codes for classification of malignancy lineage

ICD-O-3	Histology Code	Individuals (N)	Classification
9591	Malignant Lymphoma NonHodgkin	1	Lymphoid
9670	ML Small B Lymphocytic NOS	1	Lymphoid
9699	Marginal Zone B Cell Lymphoma NOS	1	Lymphoid
9710	Other Spec Nonhodgkin Lymphoma	1	Lymphoid
9732	Multiple Myeloma	4	Lymphoid
9823	Chronic Lymphocytic Leukemia Small Lympocytic Lymphoma	10	Lymphoid
9831	T-cell Large Granular Lymphocytic Leukemia	1	Lymphoid
9861	Acute Myeloid Leukemia	2	Myeloid
9863	Chronic Myeloid Leukemia NOS	1	Myeloid
9867	Acute Myelomonocytic Leukemia	1	Myeloid
9868	Other Myeloid Leukemia	2	Myeloid
9874	Acute Myeloid Leukemia with Maturation	1	Myeloid
9895	Acute Myeloid Leukemia with Multilineage Dysplasia	1	Myeloid
9945	Chronic Myelomonocytic Leukemia NOS	1	Myeloid
9950	Polycythemia Vera	3	Myeloid
9960	Chronic Myeloproliferative Disease NOS	1	Myeloid
9961	Myelosclerosis with Myeloid Metaplasia	2	Myeloid
9962	Essential Thrombocythemia	2	Myeloid
9980	Refractory Anemia	1	Myeloid
9982	Refractory Anemia with Sideroblasts	1	Myeloid
9983	Refractory Anemia with Excess Blasts	4	Myeloid
9989	Myelodysplastic Syndrome NOS	4	Myeloid
	**Total Individuals with Hematologic Malignancies**	**46**	
