# Supplementary material for: Longitudinal characterization of mosaic chromosomal alterations identifies factors influencing clonal dynamics of leukocytes
Source: Nat Commun. 2026 Jul 29;17:9192. doi: 10.1038/s41467-026-75996-5 (PMC13522525; doi:10.1038/s41467-026-75996-5)
Supplement: Supplementary file 2 [file 41467_2026_75996_MOESM2_ESM.pdf]

Corresponding author(s): Rebecca Kelly, Mitchell Machiela

Last updated by author(s): May 20, 2026

Reporting Summary

Nature Portfolio wishes to improve the reproducibility of the work that we publish. This form provides structure for consistency and transparency in reporting. For further information on Nature Portfolio policies, see our Editorial Policies and the Editorial Policy Checklist.

Statistics

For all statistical analyses, confirm that the following items are present in the figure legend, table legend, main text, or Methods section.

n/a	Confirmed	
☐	☒	The exact sample size (*n*) for each experimental group/condition, given as a discrete number and unit of measurement
☒	☐	A statement on whether measurements were taken from distinct samples or whether the same sample was measured repeatedly
☐	☒	The statistical test(s) used AND whether they are one- or two-sided<br>*Only common tests should be described solely by name; describe more complex techniques in the Methods section.*
☐	☒	A description of all covariates tested
☐	☒	A description of any assumptions or corrections, such as tests of normality and adjustment for multiple comparisons
☐	☒	A full description of the statistical parameters including central tendency (e.g. means) or other basic estimates (e.g. regression coefficient) AND variation (e.g. standard deviation) or associated estimates of uncertainty (e.g. confidence intervals)
☐	☒	For null hypothesis testing, the test statistic (e.g. *F*, *t*, *r*) with confidence intervals, effect sizes, degrees of freedom and *P* value noted<br>*Give P values as exact values whenever suitable.*
☒	☐	For Bayesian analysis, information on the choice of priors and Markov chain Monte Carlo settings
☒	☐	For hierarchical and complex designs, identification of the appropriate level for tests and full reporting of outcomes
☐	☒	Estimates of effect sizes (e.g. Cohen's *d*, Pearson's *r*), indicating how they were calculated

Our web collection on statistics for biologists contains articles on many of the points above.

Software and code

Policy information about availability of computer code

Data collection	MoChA pipeline (versions 2022-01-12; 2022-01-22; 2022-05-18; 2022-12-21) (https://github.com/freeseek/mocha); The analytic pipeline used for MoChA analysis is available at mocha.wdl.
Data analysis	R (4.2.1), PLINK (1.9)

For manuscripts utilizing custom algorithms or software that are central to the research but not yet described in published literature, software must be made available to editors and reviewers. We strongly encourage code deposition in a community repository (e.g. GitHub). See the Nature Portfolio guidelines for submitting code & software for further information.

Data

Policy information about availability of data

All manuscripts must include a data availability statement. This statement should provide the following information, where applicable:

- Accession codes, unique identifiers, or web links for publicly available datasets
- A description of any restrictions on data availability
- For clinical datasets or third party data, please ensure that the statement adheres to our policy

Genotyping array data are available on the public repository dbGaP under the study accession: phs001286.v4.p2. The informed consent document signed by the PLCO trial participants allows the use of these data by investigators for discovery and hypothesis generation in the investigation of genetic contributions to cancer,

adult diseases and novel GWAS methods. Companion phenotype data are available upon request and approval of proposal submitted to the PLCO Cancer Data Access System (CDAS). (https://cdas.cancer.gov/learn/plco/instructions/).

Research involving human participants, their data, or biological material

Policy information about studies with human participants or human data. See also policy information about sex, gender (identity/presentation), and sexual orientation and race, ethnicity and racism.

Reporting on sex and gender	All analyses of mLOX were restricted to participants of female sex as determined by genetic markers. All analyses of mLOY were restricted to participants of male sex as determined by genetic markers.
Reporting on race, ethnicity, or other socially relevant groupings	Self-reported race and ethnicity were not considered as covariates in the analyses. Cross-sectional regression analyses were adjusted for GRAF-pop ancestry percentages from ancestry-informative genotyping array markers. Polygenic risk score analyses were restricted to individuals whose genotyping array data was imputed with the European ancestry TopMed reference panel.
Population characteristics	The cross-sectional cohort is all PLCO cohort participants that gave consent for genotyping array analysis from an available blood sample. The longitudinal cohort is a subset of PLCO cohort participants with genotyping array data from a blood sample and a detectable mosaic chromosomal alteration.
Recruitment	154,807 individuals were recruited from ten sites between 1993 and 2001 (Birmingham, Alabama; Denver, Colorado; Detroit, Michigan; Honolulu, Hawaii; Marshfield, Wisconsin; Minneapolis, Minnesota; Pittsburgh, Pennsylvania; Salt Lake City, Utah; St. Louis, Missouri; Washington, DC).
Ethics oversight	All participants provided written, informed consent and the study was approved by the Institutional Review Boards at the 10 screening centers and the National Cancer Institute.

Note that full information on the approval of the study protocol must also be provided in the manuscript.

Field-specific reporting

Please select the one below that is the best fit for your research. If you are not sure, read the appropriate sections before making your selection.

☒ Life sciences ☐ Behavioural & social sciences ☐ Ecological, evolutionary & environmental sciences

For a reference copy of the document with all sections, see nature.com/documents/nr-reporting-summary-flat.pdf

Life sciences study design

All studies must disclose on these points even when the disclosure is negative.

Sample size	Cross-sectional PLCO blood-sample cohort scanned for mCAs: n= 56,324; Longitudinal cohort n = 1,490
Data exclusions	We excluded samples with poor genotyping completion rate and samples that failed MoChA quality control.
Replication	No formal replication was performed, as this is the only longitudinal collection of blood samples with genotyping array data available to our knowledge.
Randomization	This analysis did not have a randomization component. Covariates for known and suspected confounders were controlled for using statistical adjustment.
Blinding	No blinding was present in this study.

Reporting for specific materials, systems and methods

We require information from authors about some types of materials, experimental systems and methods used in many studies. Here, indicate whether each material, system or method listed is relevant to your study. If you are not sure if a list item applies to your research, read the appropriate section before selecting a response.

Materials & experimental systems

n/a	Involved in the study
☒	☐ Antibodies
☒	☐ Eukaryotic cell lines
☒	☐ Palaeontology and archaeology
☒	☐ Animals and other organisms
☒	☐ Clinical data
☒	☐ Dual use research of concern
☒	☐ Plants

Methods

n/a	Involved in the study
☒	☐ ChIP-seq
☒	☐ Flow cytometry
☒	☐ MRI-based neuroimaging

Plants

Seed stocks

Report on the source of all seed stocks or other plant material used. If applicable, state the seed stock centre and catalogue number. If plant specimens were collected from the field, describe the collection location, date and sampling procedures.

Novel plant genotypes

Describe the methods by which all novel plant genotypes were produced. This includes those generated by transgenic approaches, gene editing, chemical/radiation-based mutagenesis and hybridization. For transgenic lines, describe the transformation method, the number of independent lines analyzed and the generation upon which experiments were performed. For gene-edited lines, describe the editor used, the endogenous sequence targeted for editing, the targeting guide RNA sequence (if applicable) and how the editor was applied.

Authentication

Describe any authentication procedures for each seed stock used or novel genotype generated. Describe any experiments used to assess the effect of a mutation and, where applicable, how potential secondary effects (e.g. second site T-DNA insertions, mosiacism, off-target gene editing) were examined.
