# Supplementary material for: Longitudinal characterization of mosaic chromosomal alterations identifies factors influencing clonal dynamics of leukocytes
Source: Nat Commun. 2026 Jul 29;17:9192. doi: 10.1038/s41467-026-75996-5 (PMC13522525; doi:10.1038/s41467-026-75996-5)
Supplement: Supplementary file 3 [file 41467_2026_75996_MOESM3_ESM.pdf]

nature portfolio

Peer Review File

Longitudinal characterization of mosaic chromosomal alterations identifies factors influencing clonal dynamics of leukocytes

Corresponding Author: Dr Rebecca Kelly

This file contains all reviewer reports in order by version, followed by all author rebuttals in order by version.

Version 0:

Reviewer comments:

Reviewer #1

(Remarks to the Author)
Current research on mosaic chromosome alterations (mCAs) in leukocytes has firmly established that their presence is a significant biomarker for increased risk of a wide spectrum of conditions. In this article, Kelly et al. conducted a large longitudinal study by following 1,746 mCAs at 2-4 time points over a span of up to six years. The authors primarily focused on three types of mCAs: autosomal, loss of X (mLOX in females), and loss of Y (mLOY in males), and investigated the impact of a comprehensive set of factors on the change of the clonal expansion rates. The new findings include a few relatively surprising results, such as (1) mLOYs in <65 y.o. expand faster than older individuals, and (2) mLOYs in smokers expand slower than in non-smokers, highlighting the importance of a systematic longitudinal study on mCAs.

Major critiques
1. Statistical analyses played a major role in this manuscript. I appreciate that the authors presented the exact P values and confidence intervals in their analyses; however, two aspects remain unclear.
1) For the linear mixed model (row #516) used to test the significance of covariates (e.g., BMI) on their interactions with age, it appears that the author ran separate tests on different co-variables (row #228 and Table S4). For correlated factors, such as age, BMI, and smoking status, however, a joint analysis that includes all variables can help eliminate the biases derived from variable correlations. For instance, the proportion of never smokers typically increases with age. However, the never smokers had higher mLOY expansion than smokers, and older individuals had lower mLOY expansion rates than younger ones. How will the interactions between these factors affect the analysis?
2) Likely due to the overall sparsity of the dataset, several conclusions were drawn from relatively weak P values without multiple hypothesis corrections. For instance, 19 statistical tests were performed in Table S4, when the authors were evaluating the impact of individual factors on the expansion rates. Raw P values instead of adjusted P values were used to derive conclusions in the main text.
2. In addition, some of the interpretations of the results can be further clarified.
1) The cellular fraction of mLOX did not increase with age in the cross-sectional cohort of PLCO (row #130). In the authors' previous publication (Liu et al, Nature 2024 Extended Fig. 2), they showed that the population frequency of individuals with mLOX at >5% cellular frequency increases significantly with age in several databases, including PLCO. Can the authors discuss these seemingly contradictory findings?
2) The autosomal mCA with the highest expansion rate, chr9p copy-neutral loss of heterozygosity (CN-LOH), is suspected to be correlated with mutations in the JAK2 gene (row 321). Can't this hypothesis be tested directly using the current dataset?
3) The theoretical maximum of the mLOY cellular fraction, as used for explaining the slower expansion in older males or smokers, can also be further clarified. While linear models were used in the current analysis, did the authors observe a possible plateau when plotting expansion speed over cellular fractions?

The manuscript overall represents a significant advancement in our understanding of mCA expansion in leukocytes. Further studies with more comprehensive data will help to address a few remaining questions in this field.

Reviewer #2

(Remarks to the Author)

Summary

The study presents a comprehensive longitudinal analysis of mosaic chromosomal alterations (mCAs) in a large cohort, providing valuable insights into the factors that influence clonal dynamics over time.

Major Comments
1. Methods: The manuscript mentions that genotyping data were "harmonized and imputed as previously described." However, the authors should briefly include details on pre-imputation quality control, imputation reference panels, reference genome build, and imputation software used, as this will aid in data quality checks.

2. Methods: The array-specific mLOY detection is well explained; however, note how many samples were excluded.

3. Methods: There is insufficient explanation for why 5,699 blood samples were genotyped, but only 5,118 were used in the analytic set.

4. Methods: The mixed-model framework is appropriate, but defining tij (centred? scaled? years?) and specifying covariates in var more explicitly would improve clarity. Additionally, missing data handling (e.g., in BMI, smoking) is not discussed.

5. Methods: Samples were genotyped across multiple arrays and presumably in multiple batches. Clarify whether the array type was included as a covariate or if batch correction was performed. mCA detection can be sensitive to array-specific artifacts. If this was not performed, please confirm if the key results change if a batch covariate is included.

6. Results/Discussion: Re the cohort/results summary in Results line 117-137; in the discussion, address if this cohort is representative, unusual, etc. If no other large enough cohort exists to be able to compare (i.e. if data doesn't exist to know if it is ‘normal’ for 19.1% of the population to have mCas), then this further highlights the uniqueness of your cohort. As written, it isn’t clear if the overall results from this cohort are novel, expected, representative, unusual etc.

7. Results line 158-160. As written, this implies that 85 individuals in the longitudinal analysis had their first detected mCa in a subsequent, non-baseline, measurement. However, samples were selected for the longitudinal analysis based on detected mCas (line 139-140). This sentence either needs to be rephrased for clarity or the discrepancy expanded on.

8. Methods: More information is needed on the PRS calculation – what method was used (Ld pruning and threshold, Bayesian methods?). The data availability statement should note where the resultant PRS can be accessed, i.e. submitted to the PRS catalogue?

Minor Comments
1. The multiple sample counts across analysis stages make it difficult to track the flow. A brief flow diagram or summary table would improve clarity.

2. Clarify how CF was calculated.

3. Introduction line 67-70; a summary of the expected direction/magnitude of previously reported association between clone size and disease risk would help understand the context of this study

4. Line 72-75 – I think the authors are referring to germline variants here as occurring in driver genes (rather than somatic mutations), but being explicit would be good. Related, expanding very briefly on why such variants/genes might cause CHIP would also help non-experts understand the context

5. Line 266-268 – would be better in the intro and then briefly summarised here

Reviewer #3

(Remarks to the Author)
I co-reviewed this manuscript with one of the reviewers who provided the listed reports. This is part of the Nature Communications initiative to facilitate training in peer review and to provide appropriate recognition for Early Career Researchers who co-review manuscripts.

Reviewer #4

(Remarks to the Author)
Summary

The authors conduct a longitudinal study of 56,324 participants from the Prostate, Lung, Colorectal, Ovarian (PLCO) Cancer Screening Trial to assess the clonal dynamics of mCAs identified in these individuals. They characterized 1,746 mCAs that were measured across multiple years and identify associations between clonal growth rates and multiple parameters including: genomic location of the mCA, participate age, and smoking status. There are no ground-breaking or surprising

findings. Overall, the study was well-conducted and the paper well-written.

Comments

(1) I'm not sure why the ordinary least squares regression results are presented. The linear mixed model (LMM) appears to be the appropriate model. I recommend just presenting the results from the LMM.

(2) What is the genetic ancestral make-up of the PLCO study?

(3) Regarding comment (2), the minor allele frequency of the TCL1A variant (rs1122138) is 0.15 in Europeans and even higher in other populations (source gnomAD.) But in this study, it appears the MAF was ~ 0.11. Can the authors explain this discrepancy? I'm surprised that rs1122138 did not associate with clonal expansion rate.

(4) What reference data were used to phase the array data prior to running mocha?

(5) How were mCAs detected in the longitudinal samples? What arrays were used? Once mCAs were called longitudinally, how did you determine whether you were looking at the same mCA over time? Were the mCA end-points the same across time-points?

Reviewer #5

(Remarks to the Author)
In this manuscript by Kelly et al., the authors investigated the clonal expansion rate of mosaic chromosomal alterations (mCA) using longitudinal genotyping samples from the same individuals. The novelty of this manuscript is the investigation of mCA growth rates using longitudinal samples. Growth rates of clonal hematopoiesis driven by CHIP mutations have been investigated in the past, but such a study for mCAs was lacking. Further, the manuscript explored the relationship between mCA growth rates and potential risk factors associated with prevalence of mCA. The analyses discovered interesting findings about the landscape of clonal growth rates of autosomal and sex chromosome mCAs. The data and findings presented in the manuscript are highly valuable for the field of clonal hematopoiesis and hematologic malignancies.

I have some comments and suggestions to the authors which may enhance the presentation of the data.

1. How was the longitudinal cohort selected? Did all selected individuals have an mCA in the first sample? The following two statements are confusing. Please clarify.
"From the cross-sectional characterization of PLCO, we selected individuals with detectable mCAs having DNA available from 2 to 4 serial samples for longitudinal characterization (Extended Data Figure 3A)."
"Eighty-five individuals acquired their first mCAs during the sampling window"

2. How were positive and negative trajectories defined? Is it based on the CF difference between first and the last sample or based on the slope of clonal expansion? Please describe how expansion rates were estimated and provide the equation.

3. Fig. 2D shows a positive correlation of the median CF with the expansion rates. In contrast, Extended data figure 6 shows similar slope/growth rates for LoY with CF <0.1 and graeter than 0.1. How do the authors interprete these observations? Can you please divide the mCAs into more groups with higher initial CF? Such an analysis could help address the questions about the saturation of expansion rates.
Similar analysis (as in Extended figure 6 with more groups) for autosomal mCA would be valuable.
Expansion rate of LoY was highest among never smokers. Previous smokers had medium expansion rate and the current smokers had the lowest expansion rates. I find it interesting that never smoker and former smoker have different expansion rates. The authors discussed the differences in the initial cell fractions (0.2, 0.25, and 0.28). Analysis of additional sub-grouping of initial CF would be valuable to interpret this observation as well.

4. The authors evaluated the growth rate for LoX and LoY with and without autosomal mCAs. The presence of autosomal mCAs seem to lower the growth rate of LoX and LoY.
a. Is it due to competitive advantage of autosomal mCAs or specific patterns of autosomal mCAs cooccrurring with the LoX and LoY? Did you explore if the cooccurring autosomal mCAs were predominantly those with higher expansion rates (for example, 9p LOH)? Or did all autosomal mCAs have similar effects?
b. How does the initial CF of LoY/LoX and cooccurring autosomal mCA influence the expansion rates? Were there similar patterns of expansion if CF LoY > CF auto. mCA && CF LoY < CF auto mCA?

5. Related to the above comment, did the authors evaluate the growth rate of autosomal mCAs with and without LoX and LoY separately? Since the growth rates for LoX and LoY are different, combining these together may not be appropriate. Also, the authors evaluated growth rates for single mCA and mCA with co-occurring autosomal mCAs. Similar analysis for specific mCAs such as LOH 9p with and without other mCAs could provide valuable insights into how competing/co-occurring mCAs influence the expansion rates.

6. Considering the differences in expansion rates of mCAs with and without co-occurring mCAs, I wonder how co-ocurring CHIP could contribute to the expansion rates. As CHIP analysis requires DNA sequencing, which may not be readily

available. However, the authors could investigate whether the growth rates vary by CHIP PRS? There have been large-scale GWAS studies of CHIP, for example, pmid: 36450978 & pmid: 35835912.

7. The analysis of hematologic malignancy showed higher expansion rates for myeloid and lymphoid mCAs and higher expansion rates for individuals who developed malignancies, as expected. Did the authros perform lineage-specific analyses for myeloid mCA expansion rate and myeloid malignancies & lymphoid mCA expansion and lymphoid malignancies? If yes, please clarify. If not, what is the motivation for including lymphoid mCA in the analysis of myeloid malignancies and vice versa?

8. Please clarify the following statement. Does it mean that LoY have higher prevalence and CF due to higher expansion rate? The reference to the extended figure does not seem to be correct. Please correct it or explain it.
"The high rate of clonal expansion for mLOY mirrors the high population frequencies and cell fractions of mLOY observed in the cross-sectional characterization (Table 1, Extended Data Figure 3)."

9. Figure 2B contains bars with darker coloer with lighter background. It is difficult to view the data. Removing the background color may enhance the visibility of the data.

10. In Figure 4A, the chromosome arm legend is not useful. It is does not add any value to the results and conclusion as the chromosome number is not shown. It may not be necessary to show the chromosome arms.

Version 1:

Reviewer comments:

Reviewer #1

(Remarks to the Author)
I appreciate the thorough revisions the authors made to the manuscript, especially the additional statistical analyses and the discussion addressing the limitations of this study.

Reviewer #2

(Remarks to the Author)
The author has addressed all of my comments and I have no further concerns.

Reviewer #3

(Remarks to the Author)
I co-reviewed this manuscript with one of the reviewers who provided the listed reports. This is part of the Nature Communications initiative to facilitate training in peer review and to provide appropriate recognition for Early Career Researchers who co-review manuscripts.

Reviewer #4

(Remarks to the Author)
The authors have comprehensively responded to my previous comments. I have no further comments.

Reviewer #5

(Remarks to the Author)
I thank the authors for the revision and thoroughly addressing my comments. The manuscript presents interesting findings regarding clonal hematopoiesis driven by mosaic chromosomal alterations.

I wonder if the authors have published their codes. I could not find the information in the Data availability statement. It is recommended to publish the codes with a permanent doi.

Open Access This Peer Review File is licensed under a Creative Commons Attribution 4.0 International License, which permits use, sharing, adaptation, distribution and reproduction in any medium or format, as long as you give appropriate credit to the original author(s) and the source, provide a link to the Creative Commons license, and indicate if changes were made.
In cases where reviewers are anonymous, credit should be given to 'Anonymous Referee' and the source.
The images or other third party material in this Peer Review File are included in the article's Creative Commons license, unless indicated otherwise in a credit line to the material. If material is not included in the article's Creative Commons license and your intended use is not permitted by statutory regulation or exceeds the permitted use, you will need to obtain permission directly from the copyright holder.
To view a copy of this license, visit https://creativecommons.org/licenses/by/4.0/

Reviewer #1 (Remarks to the Author):

Current research on mosaic chromosome alterations (mCAs) in leukocytes has firmly established that their presence is a significant biomarker for increased risk of a wide spectrum of conditions. In this article, Kelly et al. conducted a large longitudinal study by following 1,746 mCAs at 2-4 time points over a span of up to six years. The authors primarily focused on three types of mCAs: autosomal, loss of X (mLOX in females), and loss of Y (mLOY in males), and investigated the impact of a comprehensive set of factors on the change of the clonal expansion rates. The new findings include a few relatively surprising results, such as (1) mLOYs in <65 y.o. expand faster than older individuals, and (2) mLOYs in smokers expand slower than in non-smokers, highlighting the importance of a systematic longitudinal study on mCAs.

Major critiques

1. Statistical analyses played a major role in this manuscript. I appreciate that the authors presented the exact P values and confidence intervals in their analyses; however, two aspects remain unclear.

1) For the linear mixed model (row #516) used to test the significance of covariates (e.g., BMI) on their interactions with age, it appears that the author ran separate tests on different co-variables (row #228 and Table S4). For correlated factors, such as age, BMI, and smoking status, however, a joint analysis that includes all variables can help eliminate the biases derived from variable correlations. For instance, the proportion of never smokers typically increases with age. However, the never smokers had higher mLOY expansion than smokers, and older individuals had lower mLOY expansion rates than younger ones. How will the interactions between these factors affect the analysis?

We thank the referee for this important comment. It is correct that we only examined univariate associations between variables of interest and the longitudinal trajectories of clonal fractions in the initial submission. In the revised manuscript we include multivariable linear mixed models that adjust for multiple confounding factors by including these factors and their interaction with time. For example, when examining the effect of the variable *var* on the trajectory, we adjust for a second variable, by including a main effect for *var2* and the introduction of *var2* with *t* in the linear mixed model, and *var3*, etc.

$$Y_{ijc} = \beta_0 + \beta_1 t_{ij} + \beta_2(var) + \beta_3(t_{ij} * var) + \beta_4(var2) + \beta_5(t_{ij} * var2) + \beta_6(var3) + \beta_7(t_{ij} * var3) + \cdots + b_{i0} + b_{i1}t_{ij} + b_{ci} + \varepsilon_{ijc}$$

For interpretation reasons, the univariate analyses uses age rather than time on study for a majority of the analyses. However, it is not possible to adjust for age when using age as the time-scale. Therefore, we used time on study as the time-scale for the multivariable analyses.

Autosomal mCAs

Model	Beta Coefficient	Standard Error	Lower 95% CI	Upper 95% CI	P-Interaction
Autosomal less than 65	0.0059	0.0021	0.0018	0.0099	(ref)
Autosomal 65 or older	0.0096	0.0021	0.0055	0.0137	0.0381
Autosomal Female	0.0059	0.0021	0.0018	0.0099	(ref)
Autosomal Male	0.0072	0.0024	0.0026	0.0119	0.4795

Autosomal Never smoker	0.0059	0.0021	0.0018	0.0099	(ref)
Autosomal Current Smoker	0.0058	0.0031	-0.0003	0.0120	0.9875
Autosomal Former Smoker	0.0074	0.0023	0.0029	0.0120	0.4252
Autosomal BMI<25	0.0076	0.0019	0.0039	0.0113	(ref)
Autosomal BMI 25-30	0.0070	0.0019	0.0032	0.0108	0.7826
Autosomal BMI 30-35	0.0075	0.0025	0.0025	0.0125	0.9717
Autosomal BMI >35	0.0115	0.0037	0.0042	0.0187	0.2925

mLOX

Model	Beta Coefficient	Standard Error	Lower 95% CI	Upper 95% CI	P-Interaction
mLOX less than 65	0.0038	0.0022	-0.0005	0.0080	(ref)
mLOX 65 or older	0.0016	0.0020	-0.0023	0.0055	0.2885
mLOX Never smoker	0.0038	0.0022	-0.0005	0.0080	(ref)
mLOX Current Smoker	0.0014	0.0032	-0.0049	0.0076	0.4604
mLOX Former Smoker	0.0072	0.0024	0.0024	0.0119	0.1293
mLOX BMI <25	0.0038	0.0022	-0.0005	0.0080	(ref)
mLOX BMI 25-30	0.0047	0.0024	0.0001	0.0093	0.6900
mLOX BMI 30-35	0.0060	0.0030	0.0001	0.0118	0.4526
mLOX BMI >35	0.0039	0.0034	-0.0028	0.0107	0.9557

mLOY

Model	Beta Coefficient	Standard Error	Lower 95% CI	Upper 95% CI	P-Interaction
mLOY less than 65	0.0253	0.0011	0.0232	0.0275	(ref)
mLOY 65 or older	0.0220	0.0010	0.0201	0.0240	0.0024
mLOY Never smoker	0.0253	0.0011	0.0232	0.0275	(ref)
mLOY Current Smoker	0.0185	0.0014	0.0157	0.0213	3.44E-05
mLOY Former Smoker	0.0222	0.0009	0.0204	0.0240	0.0063
mLOY BMI <25	0.0259	0.0014	0.0232	0.0286	(ref)
mLOY BMI 25-30	0.0246	0.0012	0.0223	0.0269	0.2871
mLOY BMI 30-35	0.0271	0.0018	0.0236	0.0305	0.4995
mLOY BMI >35	0.0277	0.0030	0.0218	0.0335	0.5398

As the referee recognizes, this analysis provides an adjustment for confounding by *var2* when studying the relationship between *var* and the longitudinal trajectory. These tables have been added as a supplementary table (Table S5) and the agreement of the univariate and multivariable analysis has been noted in the Results section:

“These relationships between individual-level factors and clonal expansion were similar in multivariable models containing all of the individual factors and their interactions with time suggesting independent effects of these specific factors (Table S5).”

2) Likely due to the overall sparsity of the dataset, several conclusions were drawn from relatively weak P values without multiple hypothesis corrections. For instance, 19 statistical tests were performed in Table S4, when the authors were evaluating the impact of individual factors on the expansion rates. Raw P values instead of adjusted P values were used to derive conclusions in the main text.

The referee is correct that we did not account for multiple comparisons in statistical testing. In the text we report raw p-values to nominate candidate factors associated with clonal expansion. Due to the sparsity of the dataset, sub analyses that partition the data into smaller groups are not well powered to make strong analytic conclusions. Accordingly, we have added the following text to the Methods and Discussion indicating these are nominal associations that are uncorrected for multiple comparisons

Methods:

“All reported p-values are not adjusted for multiple comparisons with p-values < 0.0055 meeting the Benjamini-Hochberg False Discovery Rate (FDR) multiple comparison threshold (q = 0.05) for 115 study-wide tests of significance. Tests from multivariable models were not included in the study-wide number of tests.”

Discussion:

“We also note that associations reported with individual risk factors provided nominal evidence for associations and that additional larger studies are needed to amass statistically significant evidence for multiple comparison adjusted associations of potentially weaker effect sizes.”

2. In addition, some of the interpretations of the results can be further clarified.

1) The cellular fraction of mLOX did not increase with age in the cross-sectional cohort of PLCO (row #130). In the authors’ previous publication (Liu et al, Nature 2024 Extended Fig. 2), they showed that the population frequency of individuals with mLOX at >5% cellular frequency increases significantly with age in several databases, including PLCO. Can the authors discuss these seemingly contradictory findings?

For mLOX the prevalence of females with clonal fraction >5% increases sharply around age 80 across cohort studies as reported in Liu et al. PLCO is among these cohorts, but, noticeably, it only contributes information between 55 and 79 years of age prior to the sharp increase in >5% clonal fraction observed for the 80 to 85 year-bin. In the cross-sectional PLCO sample, a simple linear regression with mLOX clonal fraction as an outcome and age at blood sample collection as a predictor was performed for the 3,559 females with mLOX. The beta value was positive (2.976), but not statistically significant. Only 155 individuals of the 3,559 (4.4%) had a clonal fraction >5%. A longitudinal cohort of older females may be better designed to capture potential elevations in mLOX clonal expansion in females in older age groups.

We have added a note on this limitation to the end of the Discussion:

“This study was limited in the representation of mLOX which, despite being 33% of the cross-sectional mCAs observed in this PLCO cohort, was only 14% of the mCAs investigated in the longitudinal sample.”

2) The autosomal mCA with the highest expansion rate, chr9p copy-neutral loss of heterozygosity (CNLOH), is suspected to be correlated with mutations in the JAK2 gene (row 321). Can’t this hypothesis be tested directly using the current dataset?

Unfortunately, we do not have paired sequencing for CHIP on these samples. To investigate the possibility for JAK2 CHIP, we computed JAK2 CHIP PRS from Kessler et al (PMID: 36450978). The same PRS calculation approach was taken as for the other scores: PLCO participants in the cross-sectional cohort with European-ancestry imputed SNPs were mean-standardized and quartiles of PRS were assigned with Q4 being highest risk. Of the 19 individuals with Chr9 p-arm CNLOH, 15 individuals were of European ancestry and had genotype calls for all variants in the JAK2 PRS. Of these 15 individuals, 4 individuals had low JAK2 PRS (Q1 + Q2) and 11 had high JAK2 PRS (Q3 + Q4), indicating potential enrichment of JAK2 CHIP in these chr9 p-arm CNLOH carriers. We performed a LMM for low vs high JAK2 CHIP PRS and identified no nominal or statistically significant difference in rate of chr9 p-arm CNLOH clonal expansion, although sample size was limited.

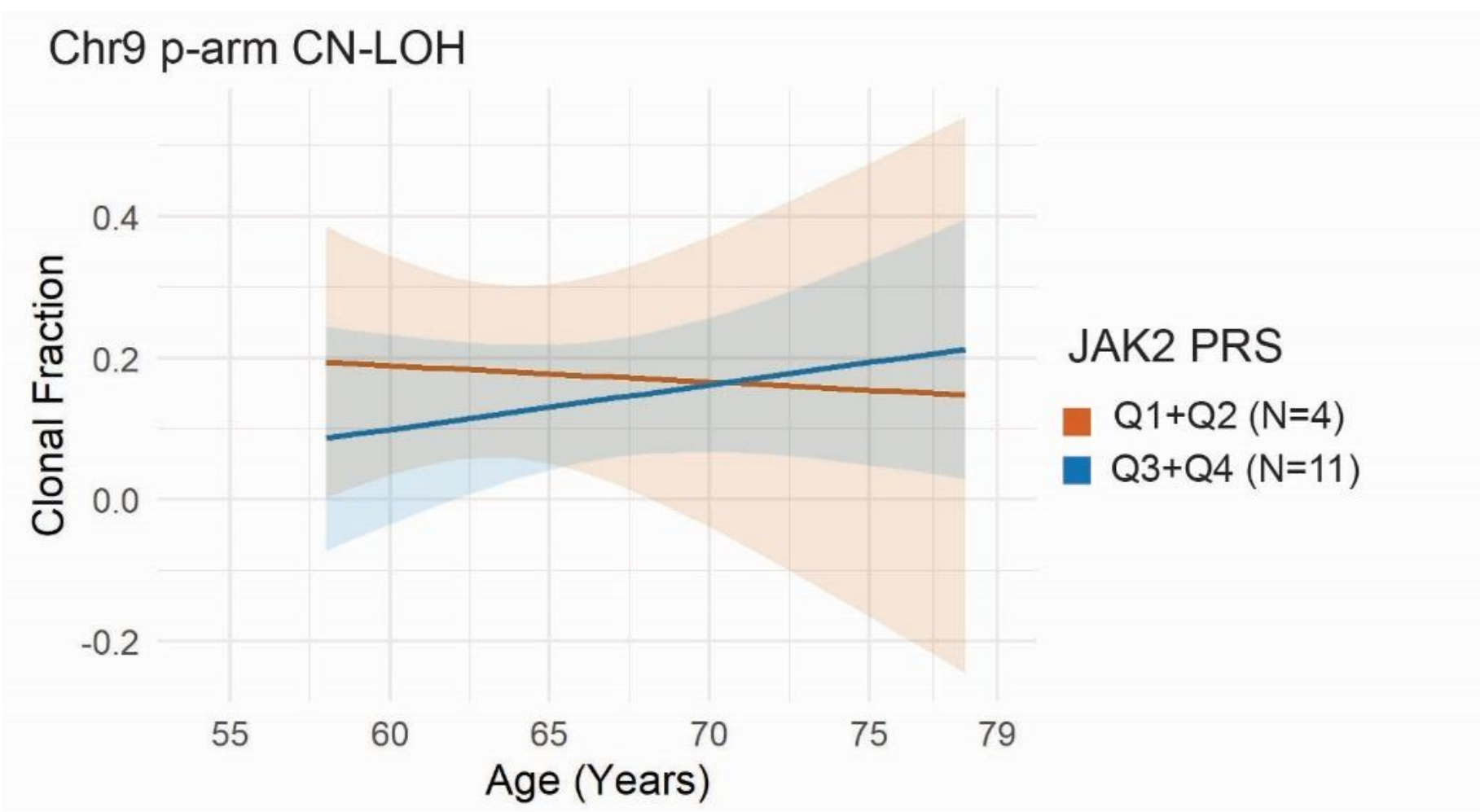

Model	Beta Coefficient	Standard Error	Lower 95% CI	Upper 95% CI	P-Interaction
JAK2_PRS_low	-0.0023	0.0128	-0.0273	0.0227	-
JAK2_PRS_high	0.0063	0.0073	-0.0080	0.0206	0.5691

3) The theoretical maximum of the mLOY cellular fraction, as used for explaining the slower expansion in older males or smokers, can also be further clarified. While linear models were used in the current analysis, did the authors observe a possible plateau when plotting expansion speed over cellular fractions?

We further expanded Figure S6 to investigate mLOY expansion across six starting clonal fraction bins and showed results from the diagnostic plots. We did not observe any significant evidence that would support a difference in mLOY expansion rate at different starting clonal fractions.

The updated Figure S6E showing these additional analyses is presented below.

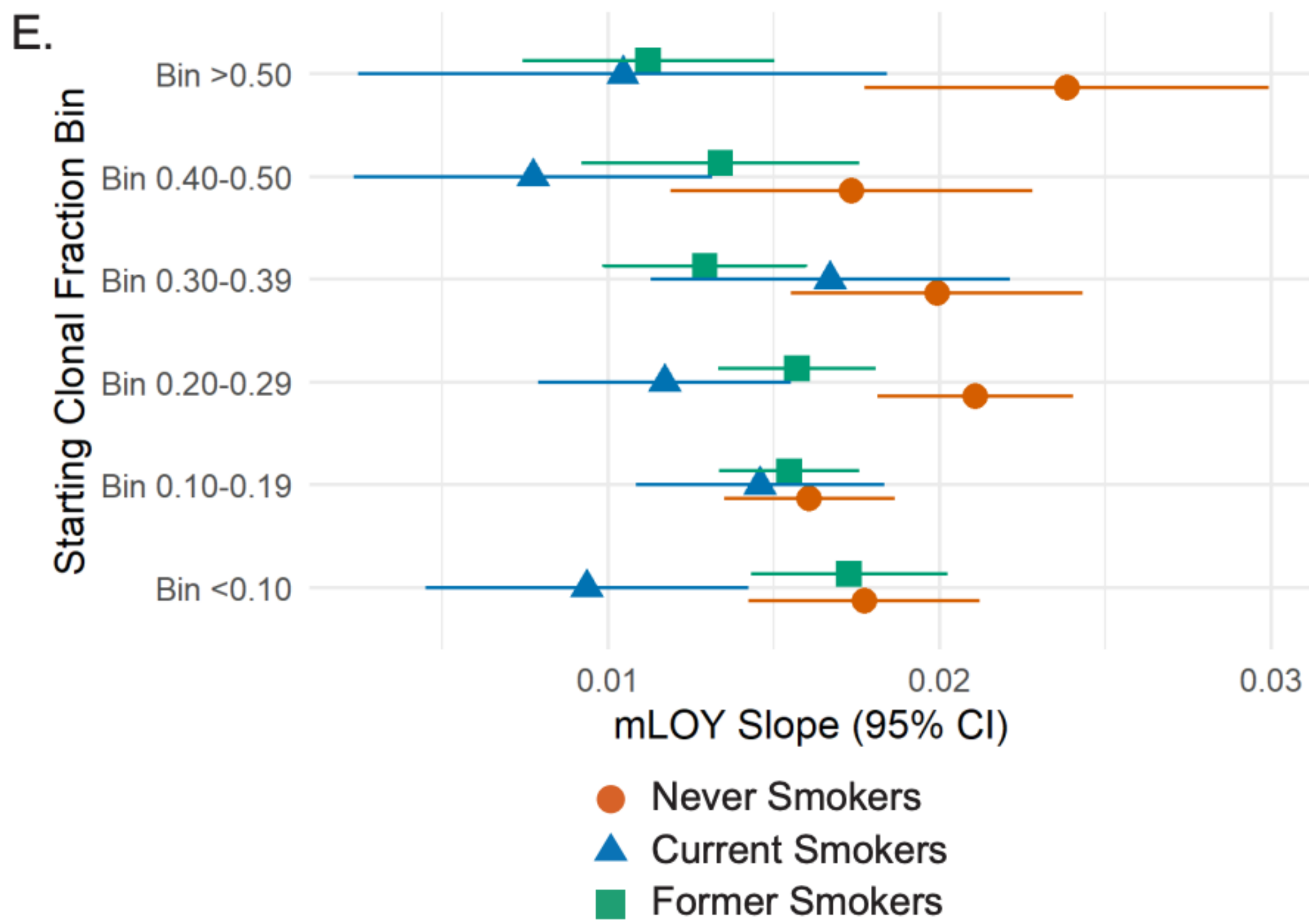

Reviewer #2 (Remarks to the Author):

Summary

The study presents a comprehensive longitudinal analysis of mosaic chromosomal alterations (mCAs) in a large cohort, providing valuable insights into the factors that influence clonal dynamics over time.

Major Comments

1. Methods: The manuscript mentions that genotyping data were "harmonized and imputed as previously described." However, the authors should briefly include details on pre-imputation quality control, imputation reference panels, reference genome build, and imputation software used, as this will aid in data quality checks.

Thank you for noticing this omission. We have updated the genotyping data processing descriptions for the PRS calculations and the MoChA pipeline to better describe the differences between the two analyses.

The Methods were updated as follows:

Cross-sectional sample genotyping and Calculation

Of the 117,551 PLCO participants who gave consent for genotyping array analysis and had an available DNA source, 110,562 were scanned with one of four genotyping arrays (Illumina Global Screening Array, OncoArray, Omni2.5 M, and OmniExpress) and had genotyping of sufficient quality to be harmonized and imputed as previously described 35. In brief, genotyping was performed at the NCI Division of Cancer Epidemiology and Genetics Cancer Genomics Research (CGR) Laboratory according to manufacturer protocols and by internal standard operating procedures. Quality control filtering removed subjects with failed output files, completion rate < 0.95, contamination > 0.10, and replicates with genotype concordance > 0.95 for a set of LD-pruned SNPs. Samples that failed a sex concordance check based on X chromosome method-of-moments F coefficient from PLINK were further screened for sex chromosome aneuploidies by STR profiling using AmpFLSTR Identifier assay and sex discordant individuals were excluded. Relatedness was screened using PLINK IBS/IBD test with pi_hat threshold of 0.1 for removal of related individuals.

Genotype imputation was performed using the TOPMed reference panel on the Michigan Imputation Server. Variants were filtered based on minor allele frequency ≤ 0.01, variant-level missingness ≥ 0.05, and with Hardy Weinberg equilibrium exact test P-Value ≤ 1.0 x 10-6 prior to imputation. The data from each genotyping platform were aligned to reference datasets (HRC-1000G-check-bim.pl) using a community recommended script modified to support TOPMed 5b as a reference panel using a pre-existing test imputation with 1000 Genomes Project subjects compared to the TOPMed 5b reference panel. Data were lifted over from GRCh37/hg19 to GRCh38/h38 using MIS. Pre-phasing used phase reference data from TOPMed release 5b on EAGLE 2.4 with imputation against the same reference panel using minimac4. Raw imputation data were partitioned into subsets based on genetic ancestry estimation using GRAFpop. Prior to imputation, “African” and “African American”, and “East Asian” and “Other Asian” were combined into “African American (combined)” and “East Asian (combined)”. Each platform and ancestry pair were cleaned according to the method from Kowalski et. al.[36] to remove variants with an Rsq < 0.3.”

Polygenic risk scores (PRS) were calculated based on independent signals associated with the outcome of interest and allelic dosage of the effect alleles were weighted by the association beta values reported for mLOY (Thompson et al.[13]), mLOX (Liu et al.[14]), and telomere length (Codd et al.[37]) for all PLCO European ancestry participants whose genotyping array data was imputed (n = 100,448)[35]. Scores were mean standardized across those participants and four quartiles of risk for each PRS were defined based on ranking individuals by score and separating into four even groups. Increasing quartiles of mLOX and mLOY PRS scores correspond to greater risk of prevalent mLOX or mLOY, respectively. Increasing quartiles of telomere length PRS score correspond to longer genetically estimated telomere lengths.

Longitudinal sample selection and genotyping

For the longitudinal mCA analyses, genotyping of additional samples was performed for a subset of individuals from the 10,747 individuals with mCAs from the cross-sectional analysis (Table 1). The study workflow for selection is described in detail in Supplemental Fig 2C.

The PLCO longitudinal samples were genotyped using two Illumina arrays: the GSAMD-24v1 array (GSAMD-24v1-0_20011747_A1.bpm; GRCh37) for the initial time points and the GSAv3Confluence array (GSAv3Confluence_20032937X371431_A1.bpm; GRCh38) for additional time points. Samples genotyped on GSAMD-24v1 array were converted to GRCh38 by realigning SNP flank sequences from the existing GRCh37 manifest files to the GRCh38 reference genome using the BWA aligner and recomputing SNP positions, following the procedure described in the gtc2vcf documentation (https://github.com/freeseek/gtc2vcf).

MoChA analysis for mCA detection

MoChA, which uses a Viterbi hidden Markov model with multiple alternate states to detect chromosomal aberrations as phase B-allele frequency deviations (https://github.com/freeseek/mocha), was applied to detect mCAs from the genotyping array data. Prior to running MoChA, the genotyping intensity data were phased using SHAPEIT4 with the 1000 Genomes Phase 3 reference panel. The phased BAF autocorrelation (baf_auto) was used to detect and estimate sample contamination, a sample call rate threshold of ≥ 0.97 and a phased BAF auto correlation (baf_auto) ≤ 0.03 were applied, based on the MoChA developers' recommendations. All analyses were based on the GRCh38 genome build. To calculate the proportion of cells with a specific type of mosaic event (clonal fraction), the B deviation (Bdev, deviation from the expected BAF value of 0.5 for heterozygous SNPs) was used: Loss: CF = 2*Bdev / (0.5+Bdev); Gain: CF = 2*Bdev / (0.5-Bdev); Neutral: CF = 2*Bdev. Detection of mLOY was called using changes in phased-BAF within the pseudo-autosomal region (PAR1) on the X chromosome for the GSA, Omni25M, and OmniExpress arrays. Detection of mLOY on Oncoarray was based on the median of log R ratio signal <= -0.15 across the male-specific region of Y chromosome, due to the low number of probes within the PAR1 region15,38. Because of this limitation, mLOY events detected from Oncoarray were removed from analyses of clonal fraction (CF) in the cross-sectional cohort. Of the 110,562 PLCO participants whose genotyping passed quality control for MoChA analysis, 56,324 had DNA collected from a blood sample and were considered for further analysis.

2. Methods: The array-specific mLOY detection is well explained; however, note how many samples were excluded.

Exclusions were made at the individual, sample, and mCA level including individuals with an mCA only detected at a cross-sectional time point (N=82); samples that either did not meet genotyping QC, MoChA QC or were collected at the same participant age (N=640); and mCAs that were undetermined type or clonal fraction (N=1,052). These details are included in the updated Methods section and updated Figure S3C included below.

C.

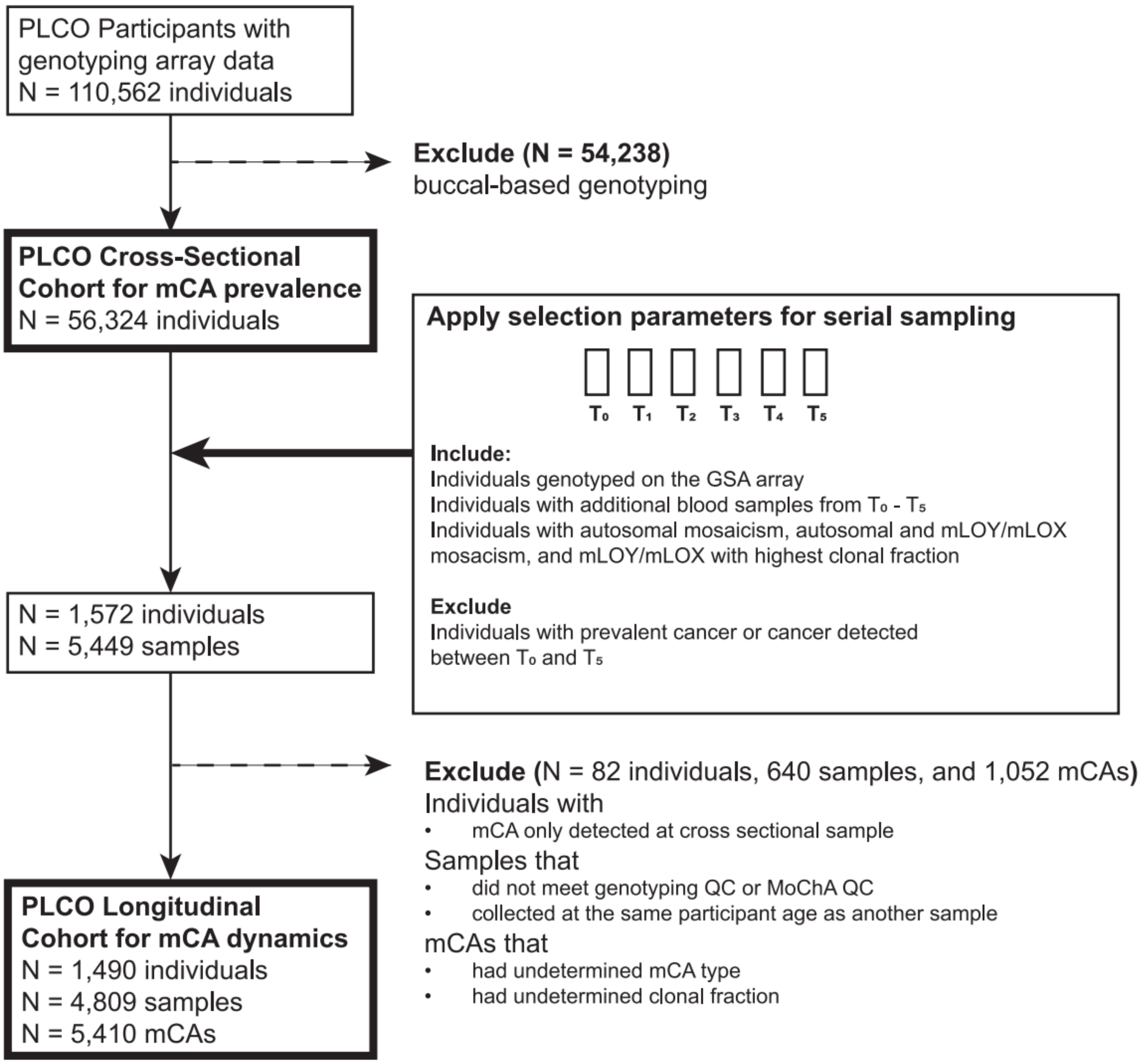

3. Methods: There is insufficient explanation for why 5,699 blood samples were genotyped, but only 5,118 were used in the analytic set.

We have added additional details to the Methods to better explain at which steps in the analysis samples were excluded from analysis. Please see response to comment #2 above.

4. Methods: The mixed-model framework is appropriate, but defining t_{ij} (centered? scaled? years?) and specifying covariates in var more explicitly would improve clarity. Additionally, missing data handling (e.g., in BMI, smoking) is not discussed.

Age at collection was scaled to 65 years by subtracting 65 from the age at sample collection so that the intercepts correspond to the clonal fraction at age 65. The following text was added to the Methods:

“Age at collection was scaled to 65 years of age for LMMs by subtracting 65 from the age at sample collection.”

The variable included in the model as *var* is the variable being tested by each LMM (e.g., sex, BMI, smoking).

$$Y_{ijc} = \beta_0 + \beta_1 t_{ij} + \beta_2 (var) + \beta_3 (t_{ij} * var) + b_{i0} + b_{i1} t_{ij} + b_{ci} + \varepsilon_{ijc}$$

We have clarified the text describing *var* in the Methods:

“□0, □1, □2, and □3 characterize population averaged changes and the effect of the tested covariate (*var*) (e.g., sex, BMI, smoking status) on these changes over time.”

Frequencies of missing covariates is included in Table 1 as “Unknown”. We also added the following text regarding the handling of missing data to the Methods:

“Individuals with missing covariate data were excluded from analyses for that covariate.”

5. Methods: Samples were genotyped across multiple arrays and presumably in multiple batches. Clarify whether the array type was included as a covariate or if batch correction was performed. mCA detection can be sensitive to array-specific artifacts. If this was not performed, please confirm if the key results change if a batch covariate is included.

We agree that genotyping array is an important covariate to consider as different arrays have different abilities to detect mCAs based on the distribution of markers across the genome. All samples genotyped in the longitudinal analysis were Illumina GSA arrays genotyped in the same batch, thus mitigating any issues from using different genotype platforms or batches. However, the initial genotyping timepoint in the PLCO Atlas effort used as the index time point (not always the baseline timepoint) for mCA detection and inclusion into the longitudinal effort was performed in a separate batch. All samples in this initial detection step included in the PLCO longitudinal effort were genotyped on GSA. Accordingly, as each traced mCA had at least one time point genotyped in a separate batch, a simple covariate for batch correction could not be easily implemented as each traced mCA had two batches: initial detection in PLCO Atlas project and subsequent timepoints genotyped in the longitudinal effort. Upon visual inspection of the clonal trajectories, we note that no notable deviations in mCA clonal fraction were observed for the Atlas time point relative to the longitudinal time points.

6. Results/Discussion: Re the cohort/results summary in Results line 117-137; in the discussion, address if this cohort is representative, unusual, etc. If no other large enough cohort exists to be able to compare (i.e. if data doesn't exist to know if it is ‘normal’ for 19.1% of the population to have mCas), then this further highlights the uniqueness of your cohort. As written, it isn’t clear if the overall results from this cohort are novel, expected, representative, unusual etc.

All individuals used for longitudinal analysis were detected in the PLCO Atlas Project, a large, prospective population of healthy US adults that were cancer-free and over the age of 55 at study entry. The frequency and distribution of individuals with detectable mCAs in PLCO was similar to that seen in other large population-based investigations such as

UK Biobank and TOPMed, suggesting that overall the frequency and distribution of detectable mCAs in PLCO is as expected in other large populations of healthy adults. To better communicate this point, we have added the following text to the Results:

"Of the 56,324 PLCO participants with genotyping data from blood-derived DNA who were screened for mCAs, 10,747 (19.1%) participants had detectable mCAs (Table 1) in line with expectations from prior population-based investigations of adult populations[9,19]."

We also added citations for the UKBB and TOPMed studies for reference:

9. Loh, P.-R. et al. Insights into clonal haematopoiesis from 8,342 mosaic chromosomal alterations. Nature 559, 350-355 (2018).

19. Jakubek, Y.A. et al. Mosaic chromosomal alterations in blood across ancestries using whole-genome sequencing. Nat Genet 55, 1912-1919 (2023).

7. Results line 158-160. As written, this implies that 85 individuals in the longitudinal analysis had their first detected mCa in a subsequent, non-baseline, measurement. However, samples were selected for the longitudinal analysis based on detected mCAs (line 139-140). This sentence either needs to be rephrased for clarity or the discrepancy expanded on.

Thanks for pointing out the need for further clarification. The PLCO study collected samples at 6 collection timepoints: T0-T5. The initial PLCO Atlas project used DNA derived from these sample collection timepoints, but not all individuals had DNA collected from the same timepoint. For example, a large fraction of individuals had DNA in the Atlas project collected from T3, while others had DNA collected at T1. We call the DNA evaluated for the Atlas timepoint the index time (e.g., T3), but other serial sample samples could be collected prior to that collection (e.g., T0, T1) or after the collection (e.g., T5). We have further clarified this in the text and Ext Data Fig 3 included below.

C.

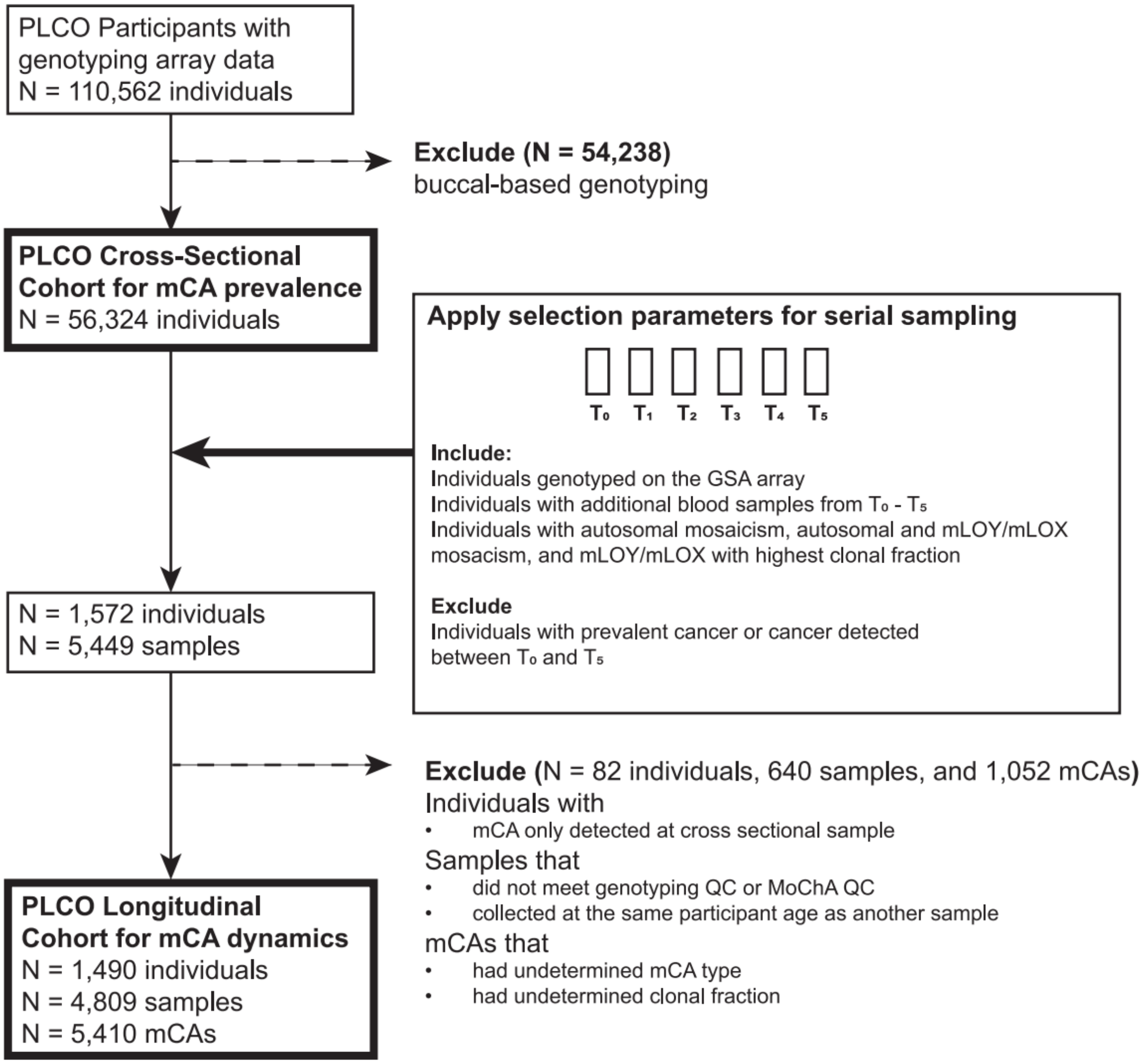

8. Methods: More information is needed on the PRS calculation – what method was used (Ld pruning and threshold, Bayesian methods?). The data availability statement should note where the resultant PRS can be accessed, i.e. submitted to the PRS catalogue?

PRS were calculated based on the independent GWAS signals reported in:

mLOY – Thompson et al. (PMID: 31748747, Table S1; 122 SNPs in PLCO)

mLOX – Liu et al. (PMID: 38867047, Table S9; 42 SNPs in PLCO)

Telomere Length – Codd et al. (PMID: 34611362, Table S1; 151 SNPs in PLCO)

CHIP Scores – Kessler et al. (PMID: 36450978, Table S20; 89 SNPs in PLCO)

SNPs from the reported GWAS studies that were available in the imputed genotyping data were used for calculation of PRS in the PLCO Atlas sample (N = 100,447). All variants included were independent signals associated with the outcome of interest and allelic dosage of the effect alleles were weighted by the association beta values.

Resulting scores were generated that summed across all variants in the scores and were subsequently mean standardized across the cross-sectional study sample (Figure S7) so reported effects were in units of a 1 standard deviation change in score.

We have added additional details the Methods:

> **"Polygenic risk scores (PRS) were calculated based on independent signals associated with the outcome of interest and allelic dosage of the effect alleles were weighted by the association beta values reported for mLOY (Thompson et al.[13]), mLOX (Liu et al.[14]), and telomere length (Codd et al.[37]) for all PLCO European ancestry participants whose genotyping array data was imputed (n = 100,448)[35]. Scores were mean standardized across those participants and four quartiles of risk for each PRS were defined based on ranking individuals by score and separating into four even groups. Increasing quartiles of mLOX and mLOY PRS scores correspond to greater risk of prevalent mLOX or mLOY, respectively. Increasing quartiles of telomere length PRS score correspond to longer genetically estimated telomere lengths.**

Minor Comments

1. The multiple sample counts across analysis stages make it difficult to track the flow. A brief flow diagram or summary table would improve clarity.

Please see Ext Data Fig 3 included in response to comment #7 above.

2. Clarify how CF was calculated.

To calculate clonal fraction, we used the B deviation (Bdev, deviation from the expected BAF value of 0.5 for heterozygous SNPs) to calculate the proportion of cells with specific type of mosaic event: Loss: CF = 2*Bdev / (0.5+Bdev); Gain: CF = 2*Bdev / (0.5-Bdev); Neutral: CF = 2*Bdev.

These details have been added to the Methods:

"To calculate the proportion of cells with a specific type of mosaic event (clonal fraction), the B deviation (Bdev, deviation from the expected BAF value of 0.5 for heterozygous SNPs) was used: Loss: CF = 2*Bdev / (0.5+Bdev); Gain: CF = 2*Bdev / (0.5-Bdev); Neutral: CF = 2*Bdev."

3. Introduction line 67-70; a summary of the expected direction/magnitude of previously reported association between clone size and disease risk would help understand the context of this study
We have added a statement about the magnitude and directionality of associations and references to two more studies that identify associations between CH clone size and malignancy and mortality risks (PMIDs: 34239136, 35857592):

> **"Given that larger clone sizes have been associated with increased risk of hematologic malignancy[2], greater disease severity and higher mortality risk[3,4], a more complete understanding of the clonal dynamics and heterogeneity of expansion for specific CH subtypes is needed to identify etiologic factors governing clonal expansion and inform the utility of specific mCAs as biomarkers of disease risk and progression."**

4. Line 72-75 – I think the authors are referring to germline variants here as occurring in driver genes (rather than somatic mutations), but being explicit would be good. Related, expanding very briefly on why such variants/genes might cause CHIP would also help non-experts understand the context

We have clarified that CHIP refers to somatic SNVs and emphasized the relationship between CHIP and malignancy risk:

> **"Somatic SNVs occurring in known leukemia-related driver genes (e.g., *DNMT3A, TET2, ASXL1*) with variant allele frequencies greater than 0.01 are referred to as clonal hematopoiesis of indeterminant potential (CHIP) and are strongly associated with hematopoietic malignancy risk."**

5. Line 266-268 – would be better in the intro and then briefly summarised here

The sentence has been moved to the end of the Introduction as suggested.

Reviewer #3 (Remarks to the Author):

I co-reviewed this manuscript with one of the reviewers who provided the listed reports. This is part of the Nature Communications initiative to facilitate training in peer review and to provide appropriate recognition for Early Career Researchers who co-review manuscripts.

Reviewer #4 (Remarks to the Author):

Summary

The authors conduct a longitudinal study of 56,324 participants from the Prostate, Lung, Colorectal, Ovarian (PLCO) Cancer Screening Trial to assess the clonal dynamics of mCAs identified in these individuals. They characterized 1,746 mCAs that were measured across multiple years and identify associations between clonal growth rates and multiple parameters including: genomic location of the mCA, participate age, and smoking status. There are no ground-breaking or surprising findings. Overall, the study was well-conducted and the paper well-written.

Comments

(1) I'm not sure why the ordinary least squares regression results are presented. The linear mixed model (LMM) appears to be the appropriate model. I recommend just presenting the results from the LMM.

Thank you for this suggestion. Instead, we have removed the ordinary least squares regression results and have replaced Table S1 to be additional descriptive statistics of the data to help orient the reader to the descriptive characteristics of the data before we report the LMM results.

Table S1. Summary of clonal fraction changes of the three most abundant mCA types

mCA Type	Median Change in Clonal Fraction	IQR	Positive change in CF
Autosomal	0.0134	-0.0015, 0.0454	527 (72.2 %)
mLOX	0.0070	-0.0040, 0.0296	124 (65.6 %)
mLOY	0.0939	0.0460, 0.1394	740 (93.3 %)

(2) What is the genetic ancestral make-up of the PLCO study?

We assessed genetic ancestry for PLCO using GRAFpop which utilizes a set of 10,000 fingerprinting variants to assign individuals into 9 ancestral groups: "African", "African American", "East Asian", "European", "Hispanic 1 (Dominican or Puerto Rican)", "Hispanic 2 (Mexican or Latin American ancestry)", "Other", "Other Asian", and "South Asia".

The cross-sectional blood-based genotyping cohort of 56,324 individuals separated by GRAF ancestry categories is as follows:

GRAF Ancestry Group	N (% total)
African	98 (0.2)
African American	2,757 (4.9)
East Asian	1,471 (2.6)
European	50,435 (89.5)
Hispanic1	127 (0.2)
Hispanic2	775 (1.4)
Other	132 (0.2)
Other Asian or Pacific Islander	434 (0.8)
South Asian	95 (0.2)

The longitudinal subset of 1,490 individuals separated by GRAF ancestry categories is as follows:

GRAF Ancestry Group	N (% total)
African	0 (0.0)
African American	37 (2.5)
East Asian	46 (3.1)
European	1,362 (91.4)
Hispanic1	1 (0.1)
Hispanic2	22 (1.5)
Other	8 (0.4)
Other Asian or Pacific Islander	10 (0.7)
South Asian	4 (0.3)

We have added a statement to the Discussion:

> **"The PLCO cross-sectional cohort was mostly European ancestry (89%) as inferred by GRAFpop, potentially limiting the generalizability of these findings across genetic ancestries."**

(3) Regarding comment (2), the minor allele frequency of the TCL1A variant (rs1122138) is 0.15 in Europeans and even higher in other populations (source gnomAD.) But in this study, it appears the MAF was ~ 0.11. Can the authors explain this discrepancy? **I'm surprised that rs1122138 did not associate with clonal expansion rate.**

We calculated the MAF of rs1122138 in the cross-sectional PLCO population to be 0.162 (55,751 individuals with rs1122138 genotype information). In the longitudinal sample, we calculated 0.129. (1,489 individuals with the rs1122138 genotype information in the longitudinal sample). This aligns well with the 11% estimated by the reviewer. The MAF is likely lower in the longitudinal cohort since the cohort is comprised of only mCA-positive individuals and rs1122138 is protective for mLOY prevalence in the cross sectional cohort:

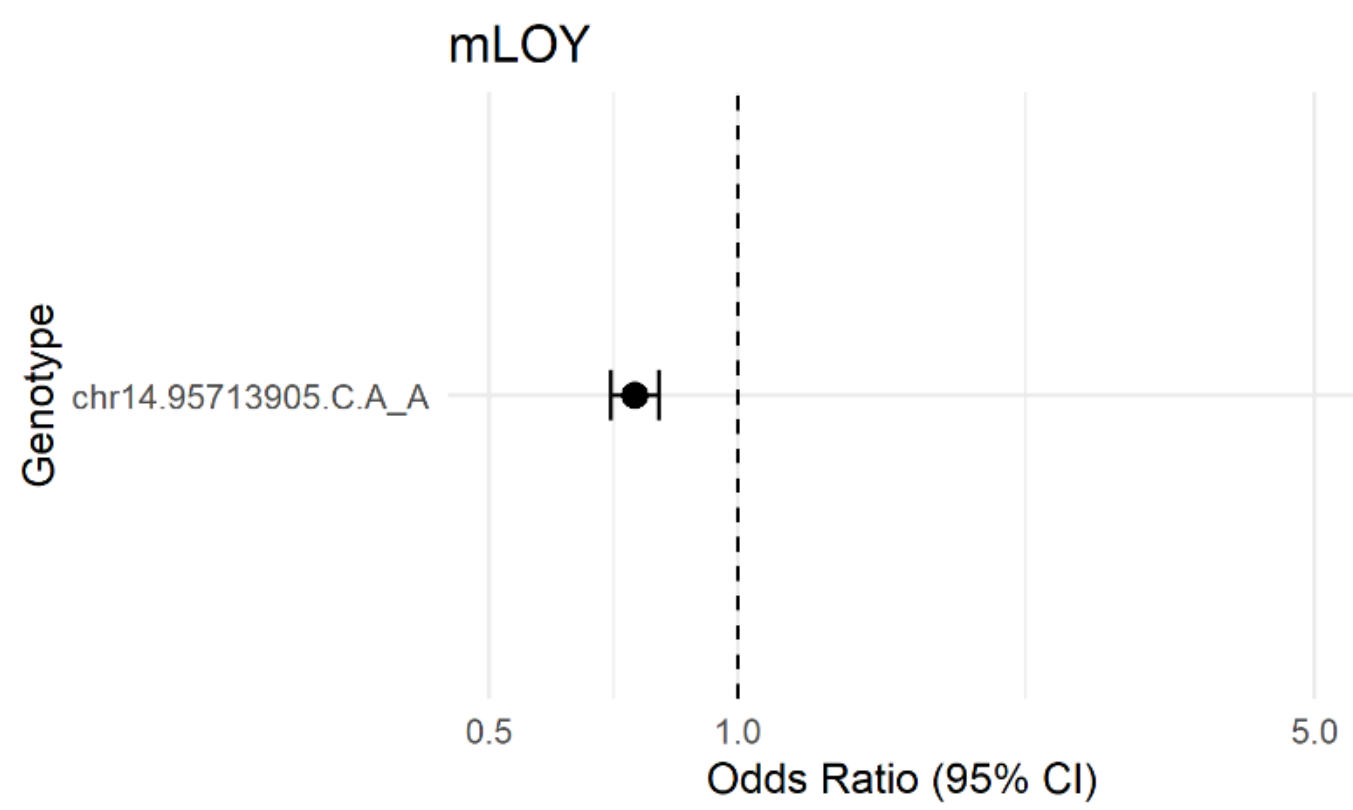

N = 26,548 males with rs1122138 SNP information.

We tested the SNP again using an additive model and reported these results in the revision. While there is no statistically significant or nominal relationship between the minor allele and clonal expansion, the point estimate for the minor allele beta (A) for autosomal mCAs is lower than that of the major allele (C) which aligns with the protective effect of the minor allele genotype observed in Pershad et al. As we also anticipated a relationship with rs1122138 and clonal expansion, we have included additional text discussing this in the Discussion.

> **"It is possible that specific components of the polygenic risk scores (e.g., variation in the *TCL1A* locus) are associated with rate of clonal expansion, as the effect of the minor allele of a clonal expansion-related SNP[17], rs1122138, had a lower expansion rate, but the difference was not statistically significant, suggesting the longitudinal sample had limited power to test individual variant associations."**

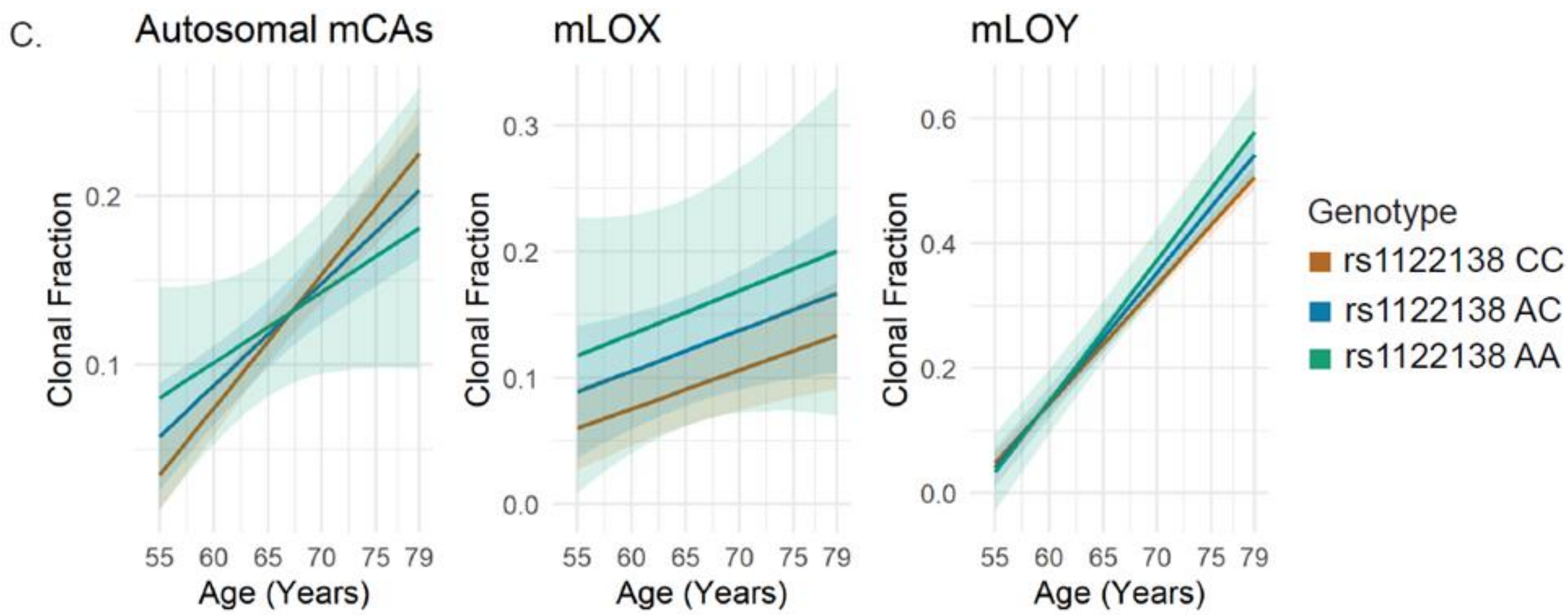

Model	Beta Coefficient	Standard Error	Lower 95% CI	Upper 95% CI	P-Interaction
Autosomal/rs1122138 CC	0.0079	0.0009	0.0062	0.0097	ref
Autosomal/rs1122138 CA	0.0061	0.0013	0.0036	0.0086	0.2100
Autosomal/rs1122138 AA	0.0042	0.0026	-0.0010	0.0094	
mLOX/rs1122138 CC	0.0031	0.0010	0.0011	0.0051	ref
mLOX/rs1122138 CA	0.0033	0.0016	0.0001	0.0064	0.9137
mLOX/rs1122138 AA	0.0035	0.0033	-0.0030	0.0099	
mLOY/rs1122138 CC	0.0191	0.0064	0.0180	0.0201	ref
mLOY/rs1122138 CA	0.0209	0.0010	0.0190	0.0228	0.1010
mLOY/rs1122138 AA	0.0227	0.0020	0.0187	0.0267	

(4) What reference data were used to phase the array data prior to running mocha?

The genotyping intensity data were phased using SHAPEIT4 with the 1000 Genomes Phase 3 reference panel "1kGP_high_coverage_Illumina" prior to running the MoChA algorithm. This detail has been added to the Methods:

> **"MoChA, which uses a Viterbi hidden Markov model with multiple alternate states to detect chromosomal aberrations as phase B-allele frequency deviations (https://github.com/freeseek/mocha), was applied to detect mCAs from the genotyping array data. Prior to running MoChA, the genotyping intensity data were phased using SHAPEIT4 with the 1000 Genomes Phase 3 reference panel (1kGP_high_coverage_Illumina). The phased BAF autocorrelation (baf_auto) was used to detect and estimate sample contamination. A sample call rate threshold of**

≥ 0.97 and a phased BAF auto correlation (baf_auto) ≤ 0.03 were applied, based on the MoChA developers' recommendations."

(5) How were mCAs detected in the longitudinal samples? What arrays were used? Once mCAs were called longitudinally, how did you determine whether you were looking at the same mCA over time? Were the mCA end-points the same across time-points?

MoChA was used as the detection algorithm to scan genotype array intensity data from Illumina Global Screening Arrays to detect mCAs in the longitudinal set. To determine whether we were looking at the same mCA over time, we used a unique mCA identifier defined based on individual ID, mCA type, and chromosomal location. For individuals with more than one mCA on a given chromosome (autosomal), an additional placeholder number was used (Figure S1). These unique event identifiers were then aggregated across longitudinal time points and visual inspection of individual events was carried out to ensure the detected events had the same genomic footprint, approximate boundaries and copy number state. Event boundaries aligned well between timepoints, with minor fluctuations observed due to uncertainty of the MoChA calling algorithm in estimating event boundaries.

Reviewer #5 (Remarks to the Author):

In this manuscript by Kelly et al., the authors investigated the clonal expansion rate of mosaic chromosomal alterations (mCA) using longitudinal genotyping samples from the same individuals. The novelty of this manuscript is the investigation of mCA growth rates using longitudinal samples. Growth rates of clonal hematopoiesis driven by CHIP mutations have been investigated in the past, but such a study for mCAs was lacking. Further, the manuscript explored the relationship between mCA growth rates and potential risk factors associated with prevalence of mCA. The analyses discovered interesting findings about the landscape of clonal growth rates of autosomal and sex chromosome mCAs. The data and findings presented in the manuscript are highly valuable for the field of clonal hematopoiesis and hematologic malignancies.

I have some comments and suggestions to the authors which may enhance the presentation of the data.

1. How was the longitudinal cohort selected? Did all selected individuals have an mCA in the first sample? The following two statements are confusing. Please clarify.

"From the cross-sectional characterization of PLCO, we selected individuals with detectable mCAs having DNA available from 2 to 4 serial samples for longitudinal characterization (Extended Data Figure 3A)." "Eighty-five individuals acquired their first mCAs during the sampling window"

The PLCO trial screening arm collected blood samples from participants at six timepoints (T0, T1, T2, T3, T4, T5). The PLCO cross-sectional timepoint genotyped in the PLCO Atlas Project was not necessarily from T0 or the same for each person, so the

sequencing of additional longitudinal samples could be from before or after the cross-sectional PLCO Atlas sample that was initially genotyped and used as the index timepoint for detecting mCAs. Because the index time point evaluated for a given individual was not always T0, it is possible that we were able to observe incidence of the detected mCA (i.e., earlier timepoints the mCA was not detectable). In the above text mentioned by the reviewer, we are making a distinction of the mCA being observed in an individual's first chronological sample (prevalent) or if it was first detected in a sample after their first chronological sample (incident) (Extended Data Table 1). We have clarified this in the text and in the new and Ext Data Fig 3 included below.

C.

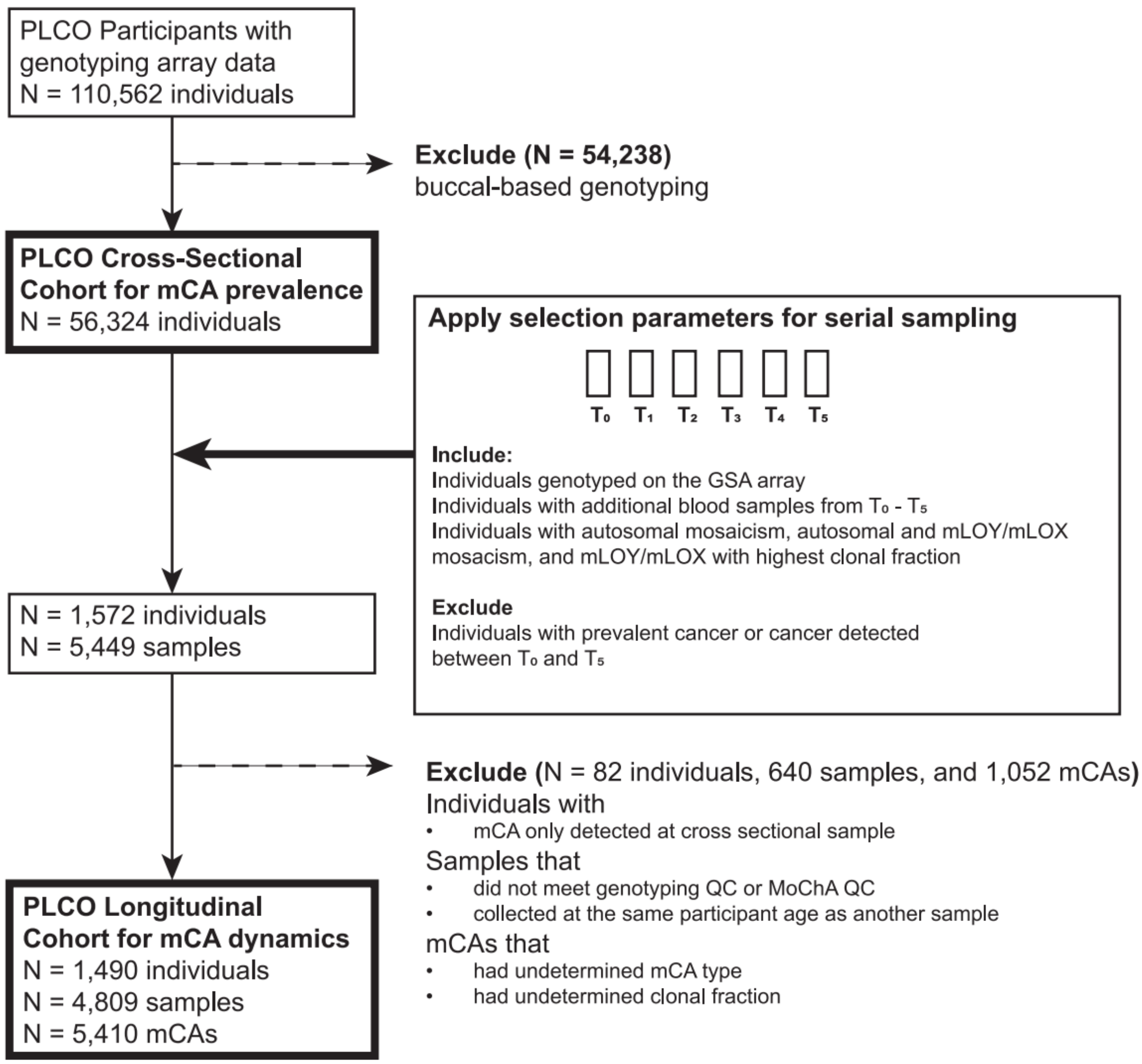

2. How were positive and negative trajectories defined? Is it based on the CF difference between first and the last sample or based on the slope of clonal expansion? Please describe how expansion rates were estimated and provide the equation.

Your intuition is correct: positive and negative trajectory for the initial data inspection was based on clonal fraction difference between an individual's last and first sample. We have clarified the calculation of trajectory in the results. We have also modified this section of the results to be a description of the longitudinal clonal fraction trajectories instead of regression models.

The following has been added to the results:

"We evaluated initial trajectories by calculating the difference in clonal fraction between the last and the first observations of an mCA. Of these 1,712 mCAs, 1,391 (81.3%) had positive trajectories, with mLOY having the highest percentage of positive trajectories (93.3%) and mLOX having the lowest percentage of positive trajectories (65.6%) (Table S1)."

3. Fig. 2D shows a positive correlation of the median CF with the expansion rates. In contrast, Extended data figure 6 shows similar slope/growth rates for LoY with CF <0.1 and graeter than 0.1. How do the authors interprete these observations? Can you please divide the mCAs into more groups with higher initial CF? Such an analysis could help address the questions about the saturation of expansion rates. Similar analysis (as in Extended figure 6 with more groups) for autosomal mCA would be valuable. Expansion rate of LoY was highest among never smokers. Previous smokers had medium expansion rate and the current smokers had the lowest expansion rates. I find it interesting that never smoker and former smoker have different expansion rates. The authors discussed the differences in the initial cell fractions (0.2, 0.25, and 0.28). Analysis of additional sub-grouping of initial CF would be valuable to interpret this observation as well.

We thank the reviewer for highlighting these interesting observations. The positive relationship between cross sectional cell fraction and clonal expansion rate suggests that clones with higher clonal expansion rates in general also tend to have higher cell fractions observed cross-sectionally. As the reviewer highlights, from our observations the opposite does not appear to be true: a higher initial cell fraction does not necessarily imply a higher clonal expansion rate. This was originally presented for mLOY in our manuscript: we observe mLOY to have a higher clonal expansion rate than autosomal or mLOX events, but when we divide mLOY into groups by initial cell fraction, we do not observe differences in clonal expansion rate. To investigate this relationship further, we expanded Figure S6 to investigate mLOY expansion across six starting clonal fraction bins and showed results from the diagnostic plots. We did not observe any evidence that would support a difference in mLOY expansion rate at different starting clonal fractions. Our results are included in updated Figure S6 included below.

Extended Data Figure 6. Starting clonal fraction and clonal expansion of mLOY

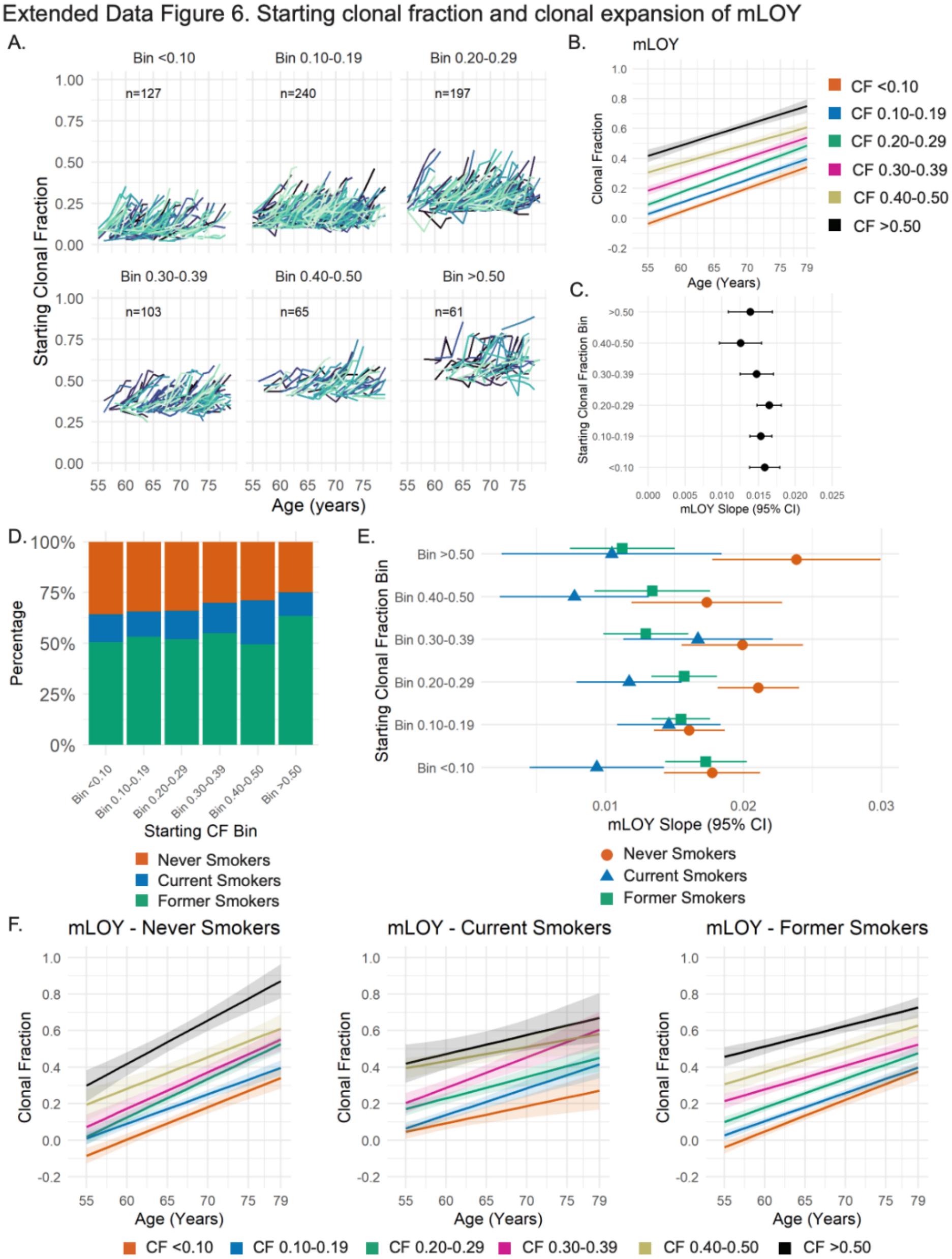

And added to the results section:

"To investigate the impact of starting clonal fraction on mLOY expansion, we categorized mLOY trajectories by starting clonal fraction and compared the effects of mLOY starting clonal fraction (Extended Data Figure 6A,B,C; Table S4). We observed no difference in slope across starting clonal fraction. Though current smokers were enriched in higher starting clonal fraction bins, their rate of expansion was consistently lower than never smokers at every starting clonal fraction, further confirming that the impact of smoking is not due to a difference in starting mLOY clonal fraction (Extended Data Figure 6D,E,F; Table S4). The rate of expansion in former smokers showed some nominal differences across starting bins, suggesting there could be some changes in rates of expansion in former smokers depending on their starting clonal fraction bin."

4. The authors evaluated the growth rate for LoX and LoY with and without autosomal mCAs. The presence of autosomal mCAs seem to lower the growth rate of LoX and LoY.

a. Is it due to competitive advantage of autosomal mCAs or specific patterns of autosomal mCAs cooccrurring with the LoX and LoY? Did you explore if the cooccurring autosomal mCAs were predominantly those with higher expansion rates (for example, 9p LOH)? Or did all autosomal mCAs have similar effects?

To further investigate the relationships between multiple co-occurring mCAs and relative timing of the two alterations, we performed additional analysis for Figure 2E, F, G, and H. The results have been added to Figure 2.

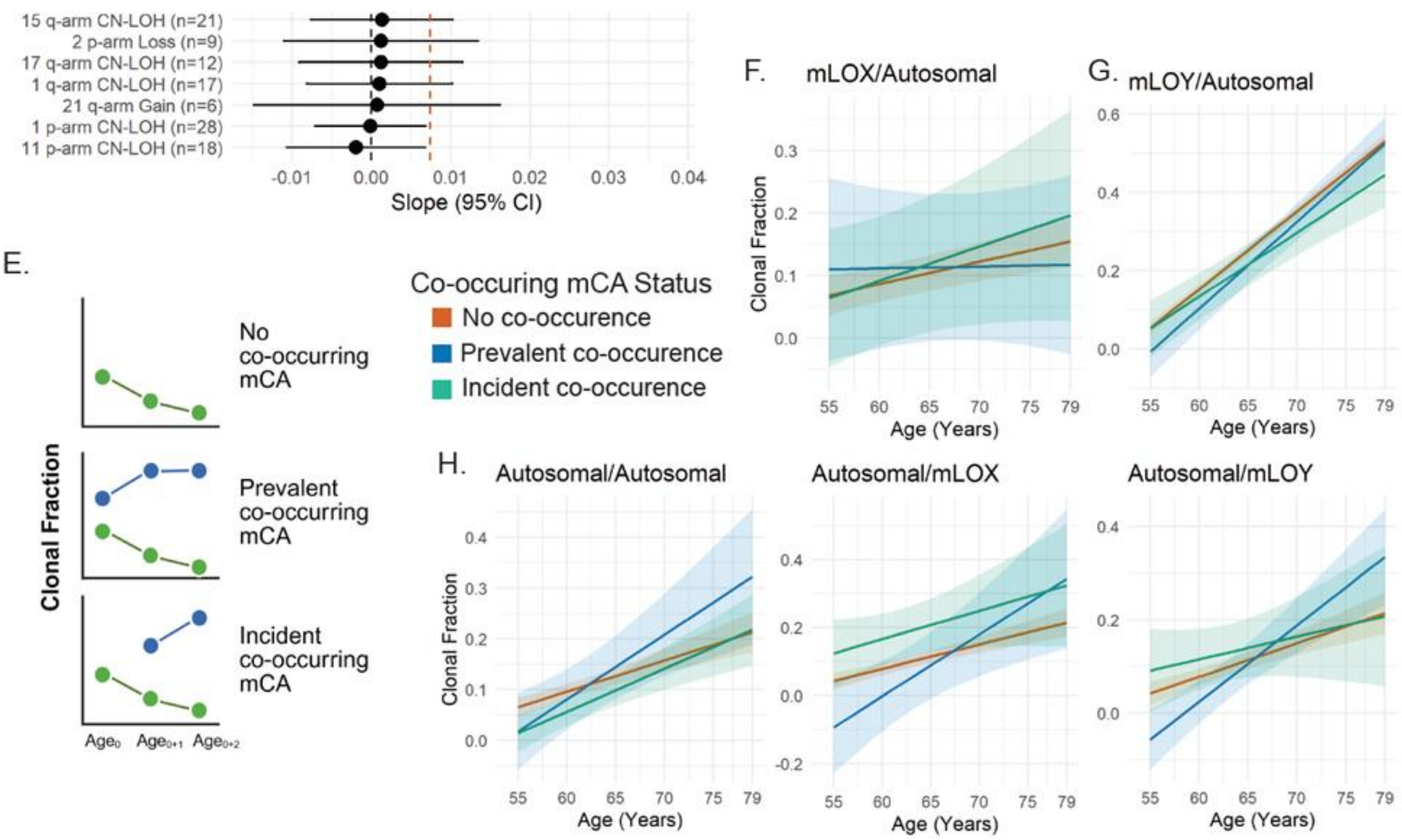

By classifying the co-occurring mCAs as prevalent and incident, we are now able to detect a higher expansion rate of autosomal mCAs that have prevalent mLOY.

We have revised the interpretation of the multiple mCA associations in the Results:

> **"We restricted the analysis of co-occurring mCAs to individuals with one or two mCAs observed during the study period and compared the rate of the mCA expansion in individuals with co-occurrence to those with only one mCA of that type (no co-occurrence). The temporal relationship between co-occurring mCAs was considered by identifying if co-occurring mCAs were observed together at an individual's first mCA-positive sample (prevalent co-occurring) or if one of the mCAs was observed after the initial mCA (incident co-occurring) (Figure 2E).**

> Among females with mLOX, expansion of mLOX with prevalent co-occurring autosomal mCAs was lower than individuals with no co-occurrence (rate$_{mLOX_prev_auto}$ = 0.03% per year age, 95% CI=[-0.68,0.74]; versus rate$_{mLOX_no_co\text{-}occur}$= 0.36% per year age, 95% CI=[0.17,0.54], $P_{interaction}$=0.3825) and higher in individuals with an incident autosomal mCA (rate$_{mLOX_inc_auto}$ = 0.55% per year age, 95% CI=[-0.16,0.1.26]; $P_{interaction}$=0.6066), but neither relationship was statistically significant (Figure 2F, Table S3). The trend was the opposite for males with mLOY. Males with mLOY and prevalent autosomal mCAs had higher rates of expansion (rate$_{mLOY_prev_auto}$ = 2.22% per year age, 95% CI=[1.81,2.62]; versus rate$_{mLOY_no_co\text{-}occur}$= 1.99% per year age, 95% CI=[1.88,2.10], $P_{interaction}$=0.2820), and lower rates of expansion with incident autosomal mCAs (rate$_{mLOY_inc_auto}$ = 1.64% per year age, 95% CI=[1.18,2.09]; $P_{interaction}$=0.1420), but neither relationship was statistically significant (Figure 2G).
>
> For autosomal mCAs, autosomal mCAs with other prevalent co-occurring autosomal mCAs (rate$_{auto_prev_auto}$=1.24% per year age, 95% CI=[0.54,1.95]; $P_{interaction}$=0.0883) and incident co-occurring autosomal mCAs (rate$_{auto_inc_auto}$ = 0.83% per year age, 95% CI=[0.47,1.19]; $P_{interaction}$=0.2958) had higher rates of expansion compared to those with only one autosomal (rate$_{auto_no_co\text{-}occur}$= 0.61% per year age, 95% CI=[0.41,0.81] (Figure 2H). For autosomal mCAs and prevalent and incident mLOX, the rate of autosomal mCA clonal expansion was higher in individuals with co-occurrence (rate$_{auto_prev_mLOX}$ =1.82% per year age, 95% CI=[0.67,2.96]; versus rate$_{auto_no_co\text{-}occur}$=0.61% per year age, 95% CI=[0.41,0.81], $P_{interaction}$=0.0658) and (rate$_{auto_inc_mLOX}$ =0.83% per year age, 95% CI=[-0.13,1.79]; $P_{interaction}$=0.8175, but not significantly. For autosomal mCAs and prevalent mLOY, autosomal mCAs with prevalent mLOY co-occurrence had a higher expansion rate which was statistically significant at the FDR threshold (rate$_{auto_prev_mLOY}$ =1.64% per year age, 95% CI=[1.04, 2.23]; versus rate$_{auto_no_co\text{-}occur}$= 0.72% per year age, 95% CI=[0.47, 0.97], $P_{interaction}$= 0.0055) while the effect of incident mLOY did not significantly alter the rate of expansion (rate$_{auto_inc_mLOY}$ = 0.49 % per year age, 95% CI=[-0.36, 1.34]; $P_{interaction}$=0.6069). This difference in the effect of prevalent and incident mLOY on autosomal mCA expansion suggests that mLOY and autosomal mCAs could have cooperative expansion but that newly acquired mLOY could have a disruptive impact on autosomal mCA clonal expansion.”

We have revised the interpretation of the multiple mCA associations in the Discussion:

> “Our longitudinal investigation likewise observed individuals with autosomal mCAs and prevalent co-occurring mLOY to have higher rates of clonal expansion suggesting potential cooperation between autosomal mCAs and mLOY; however, this increased rate was not observed for autosomal mCAs with incident co-occurring mLOY which could indicate different dynamics with newly acquired mLOY.”

To consider prevalent and incident co-occurring mCAs, we dichotomized the enriched autosomal mCAs based on the average autosomal expansion rate from Figure 2C (</≥0.70% per year age) to see whether one group was more represented in the co-occurring autosomal mCAs. (Corresponding with Figure 2H middle and right panels) We observed no evidence to suggest the lower expansion rates of mLOX and mLOY with co-occurring autosomal mCAs was due to higher frequency of autosomal mCAs with higher expansion rate co-occurring with mLOY.

Co-occurrence and enriched mCA expansion rate:

Enriched Autosomal mCA Expansion Category	mLOX prevalent	mLOX incident	mLOY prevalent	mLOY incident
low (<0.70%)	3	4	21	6
high (≥0.70%)	4	3	10	6
not enriched auto mCA	1	5	8	6

The autosomal mLOY co-occurrences were more likely to be prevalent with an mCA with an average low rate of clonal expansion. Interestingly, the rate of autosomal mCAs co-occurring with prevalent mLOY had higher rates of expansion than autosomal mCAs alone, suggesting that even though these mCAs are more likely to be low on average, their co-occurrence with mLOY may influence their expansion rate.

b. How does the initial CF of LoY/LoX and cooccurring autosomal mCA influence the expansion rates? Were there similar patterns of expansion if CF LoY > CF auto. mCA && CF LoY < CF auto mCA?

Restricting to individuals with only 2 co-occurring mCAs:

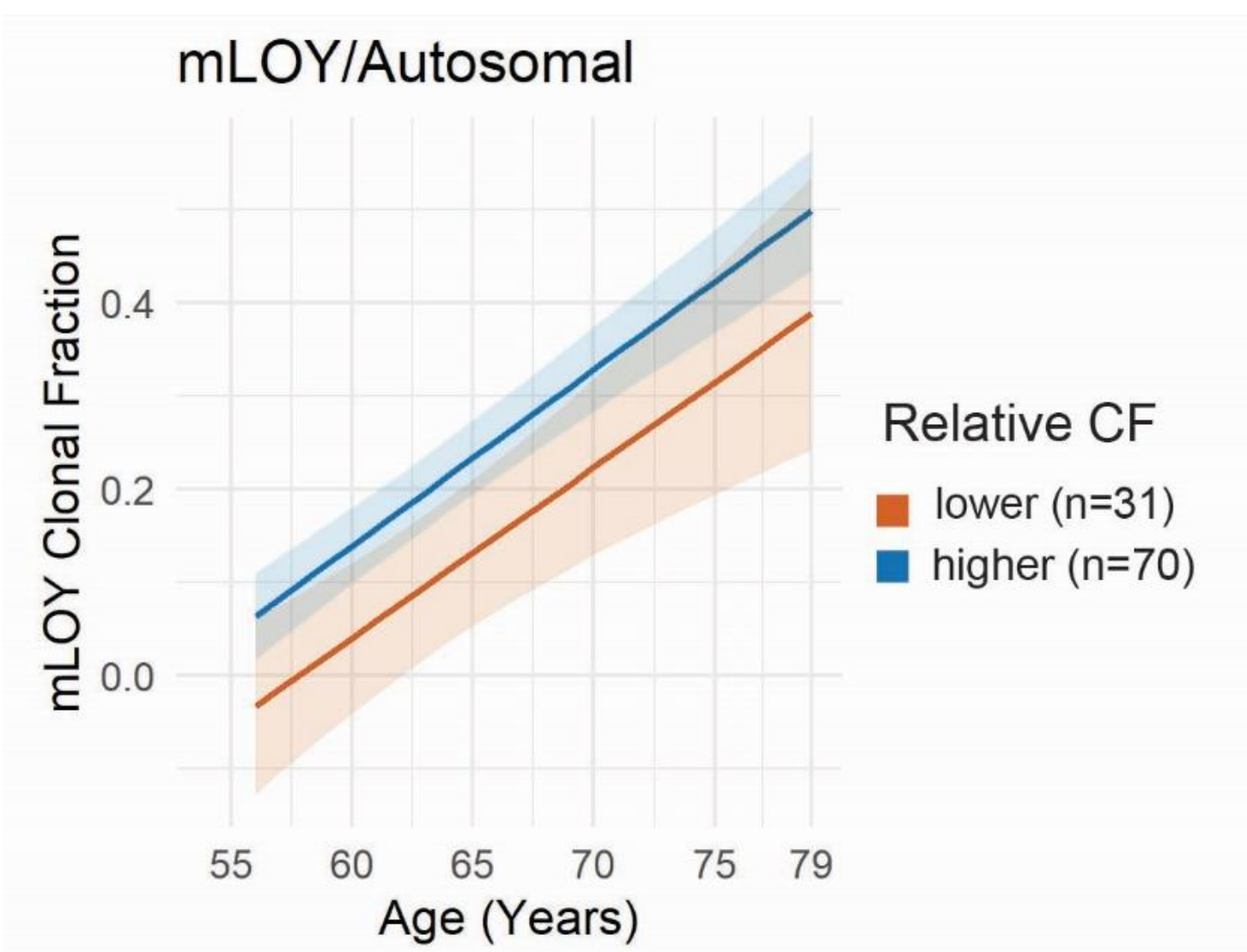

Relative CF mLOY	mLOY
equal to autosomal mCA	3
higher than autosomal mCA	70
lower than autosomal mCA	31

mLOY clonal expansion was not impacted by the relative clonal fraction of the co-occurring mCAs.

5. Related to the above comment, did the authors evaluate the growth rate of autosomal mCAs with and without LoX and LoY separately? Since the growth rates for LoX and LoY are different, combining these together may not be appropriate. Also, the authors evaluated growth rates for

single mCA and mCA with co-occurring autosomal mCAs. Similar analysis for specific mCAs such as LOH 9p with and without other mCAs could provide valuable insights into how competing/co-occurring mCAs influence the expansion rates.

We also evaluated the growth rate of autosomal mCAs with and without sex chromosome mosaicism separately for mLOX and mLOY. These comparisons are available in Figure 2 included below.

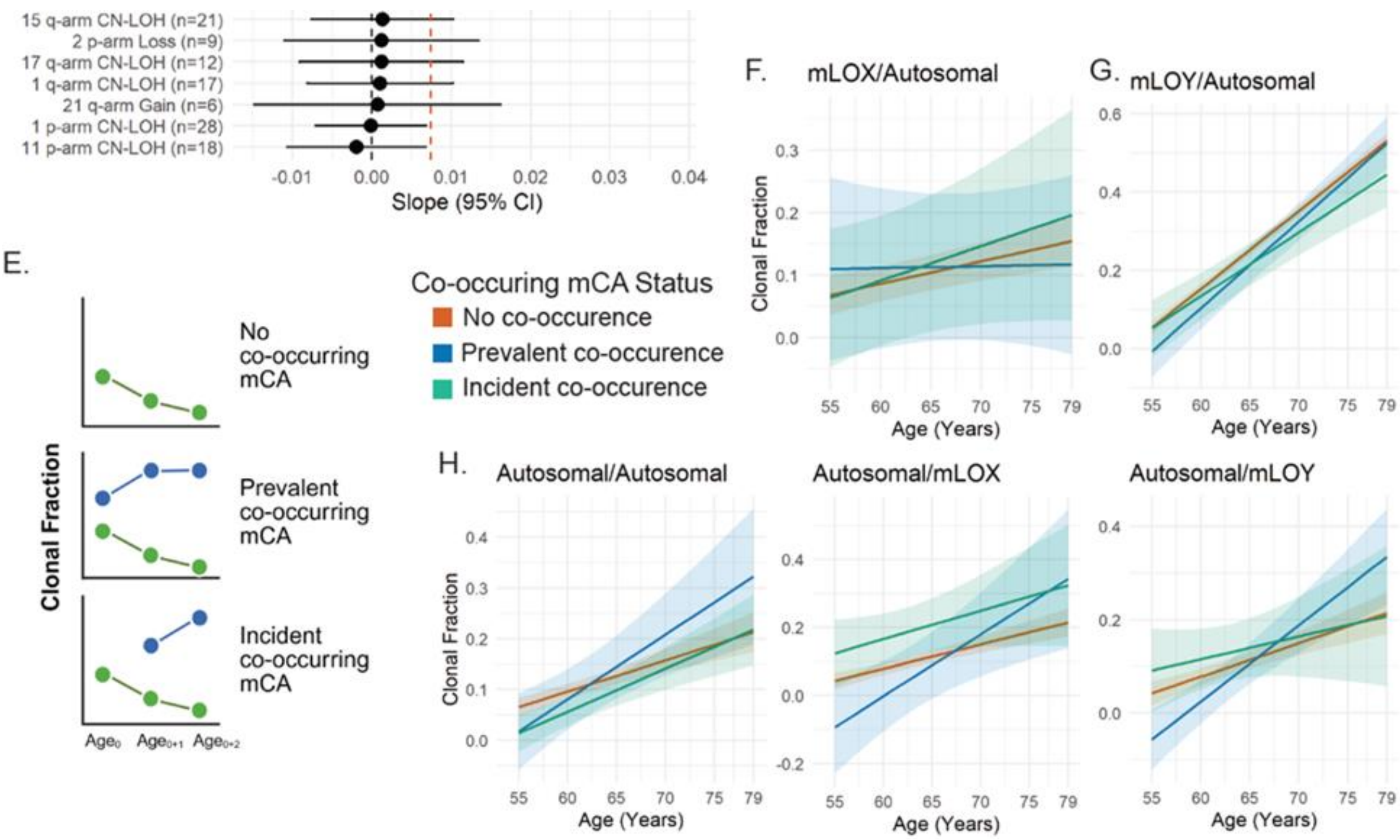

Unfortunately, in our sample set, comprehensively evaluating specific mCA combinations is difficult due to the limited sample size of individuals harboring recurrent co-occurrences.

6. Considering the differences in expansion rates of mCAs with and without co-occurring mCAs, I wonder how co-ocurring CHIP could contribute to the expansion rates. As CHIP analysis requires DNA sequencing, which may not be readily available. However, the authors could investigate whether the growth rates vary by CHIP PRS? There have been large-scale GWAS studies of CHIP, for example, pmid: 36450978 & pmid: 35835912.

We share the same enthusiasm for investigating mCA co-occurrences with CHIP but lack the exome sequencing data in PLCO to perform these analyses. To evaluate the potential of using CHIP PRS, we did an exploratory analysis of chr 9 CNLOH and JAK2 PRS in our response to Reviewer #1 Comment #2-2. Please see the results of the model below.

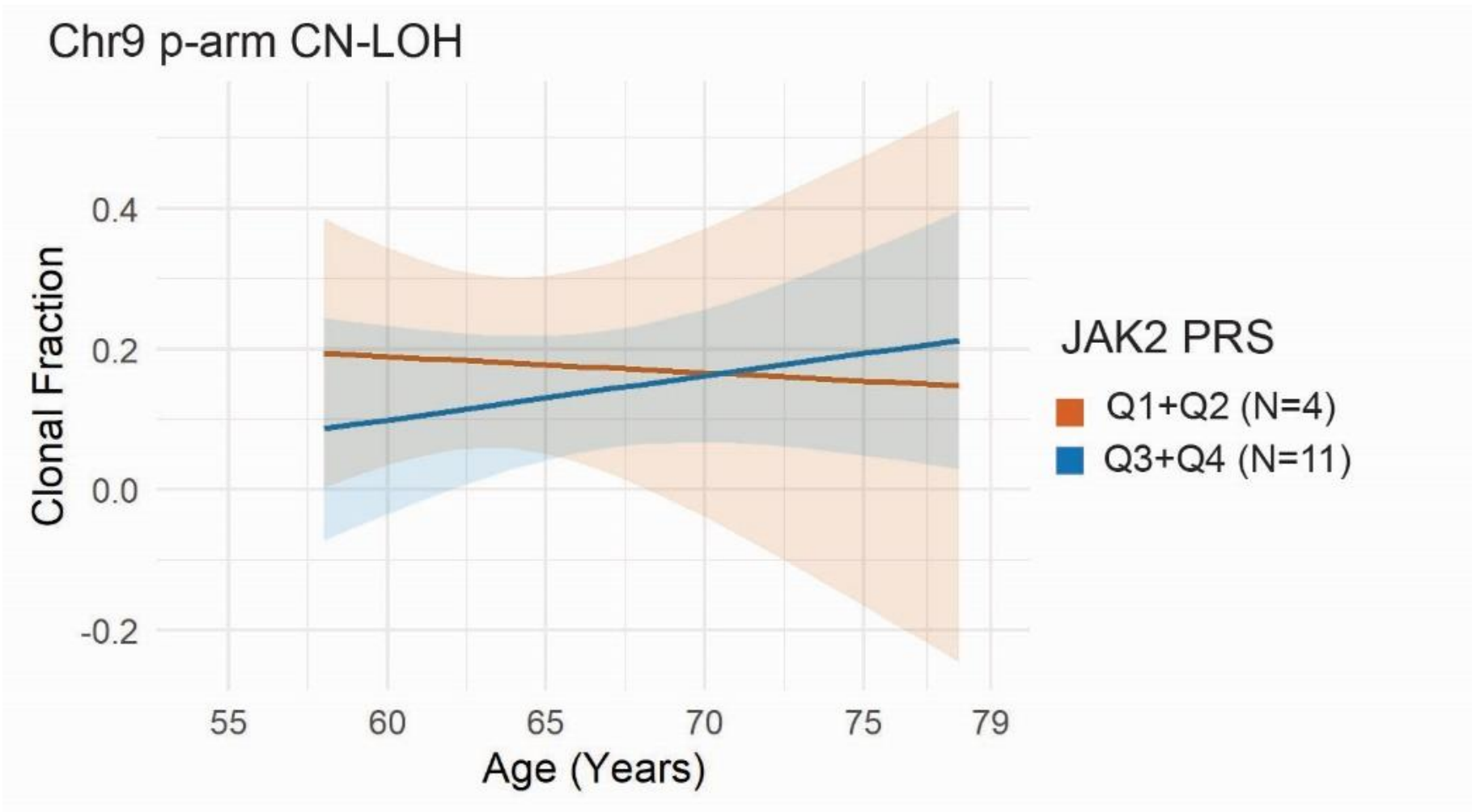

Model	Beta Coefficient	Standard Error	Lower 95% CI	Upper 95% CI	P-Interaction
JAK2_PRS_low	-0.0023	0.0128	-0.0273	0.0227	-
JAK2_PRS_high	0.0063	0.0073	-0.0080	0.0206	0.5691

7. The analysis of hematologic malignancy showed higher expansion rates for myeloid and lymphoid mCAs and higher expansion rates for individuals who developed malignancies, as expected. Did the authors perform lineage-specific analyses for myeloid mCA expansion rate and myeloid malignancies & lymphoid mCA expansion and lymphoid malignancies? If yes, please clarify. If not, what is the motivation for including lymphoid mCA in the analysis of myeloid malignancies and vice versa?

For the analysis of mCAs in individuals diagnosed with a subsequent malignancy (Table S8, Figure 4C,D), we wanted to remain agnostic to prior designations of lymphoid/myeloid-mCA and simply test the expansion rate of any autosomal mCA, mLOX, or mLOY observed in individuals actually diagnosed with a cancer which could be related to additional factors besides the characteristics of their mCA (4C). Figure 4D is stratified by the malignancy outcome regardless of the associated lineage of the mCA from Figure 4B.

We performed the analyses this way to differentiate the rates of "mCAs-associated with malignancy risk from other studies" and "mCAs from individuals diagnosed with subsequent malignancy in PLCO" since 1.) not all individuals with malignancy-associated mCAs progress to a malignancy and 2.) not all mCAs occurring in diagnosed individuals are independently associated with disease risk.

		ICD-O-3 Code	
		Lymphoid	Myeloid
Niroula classification	Lymphoid	7	2
	Myeloid	1	10
	Non-Specific	1	6
	Not Enriched	4	5
	**Total**	**13**	**23**

Of the 139 individuals with lymphoid-associated mCAs, only 7 went on to develop a lymphoid malignancy (5.0%). Of the 137 individuals with myeloid-associated mCAs, only 10 went on to develop a myeloid malignancy (7.3%).

We have clarified the differences between the malignancy-related analyses in the Results:

> **"To investigate the relationship between hematologic malignancy risk and clonal expansion rate, we measured both the clonal expansion rate of enriched autosomal mCA types associated with malignancy risk and the clonal expansion rate of mCAs in individuals who were later diagnosed with hematologic malignancies. A study by Niroula and colleagues found relationships between specific mCA types and risk of hematologic malignancies occurring in the lymphoid and myeloid lineages[25]. We classified enriched mCAs in the longitudinal cohort based on the Niroula malignancy-specific lineage groups and measured the rate of clonal expansion for these groups (Figure 4A, Tables S7, S8). Compared to mCAs with no associated malignancy lineage type, malignancy-associated mCAs had higher expansion rates, especially for myeloid malignancy associated mCAs ($rate_{non\text{-}specific}$=0.39% per year age, 95% CI=[0.12, 0.66], $rate_{lymphoid}$=0.84% per year age, 95% CI=[0.54, 1.15] $P_{interaction}$=0.0276; and $rate_{myeloid}$=1.32% per year age, 95% CI=[1.00, 1.65], $P_{interaction}=1.48 \times 10^{-5}$) (Figure 4B, Table S7). Of individuals with enriched autosomal mCAs, 5.0% of individuals with lymphoid associated mCAs went on to develop a subsequent lymphoid hematologic malignancy, while 7.3% of individuals with myeloid-associated mCAs went on to develop a myeloid malignancy."**

8. Please clarify the following statement. Does it mean that LoY have higher prevalence and CF due to higher expansion rate? The reference to the extended figure does not seem to be correct. Please correct it or explain it.

"The high rate of clonal expansion for mLOY mirrors the high population frequencies and cell fractions of mLOY observed in the cross-sectional characterization (Table 1, Extended Data Figure 3)."

Thank you for noting that the wrong figure was referenced. It has been corrected to refer to Extended Data Figure S1 in the revised version. The statement has also been reworded to remove ambiguity.

> **"The high rate of clonal expansion for mLOY estimated in the longitudinal data corresponds with the high population frequencies and cell fractions of mLOY observed in the cross-sectional characterization (Table 1, Extended Data Figure S1)."**

9. Figure 2B contains bars with darker coloer with lighter background. It is difficult to view the data. Removing the background color may enhance the visibility of the data.

The background color has been made lighter to increase contrast between the background and the bars. The revised figure is below:

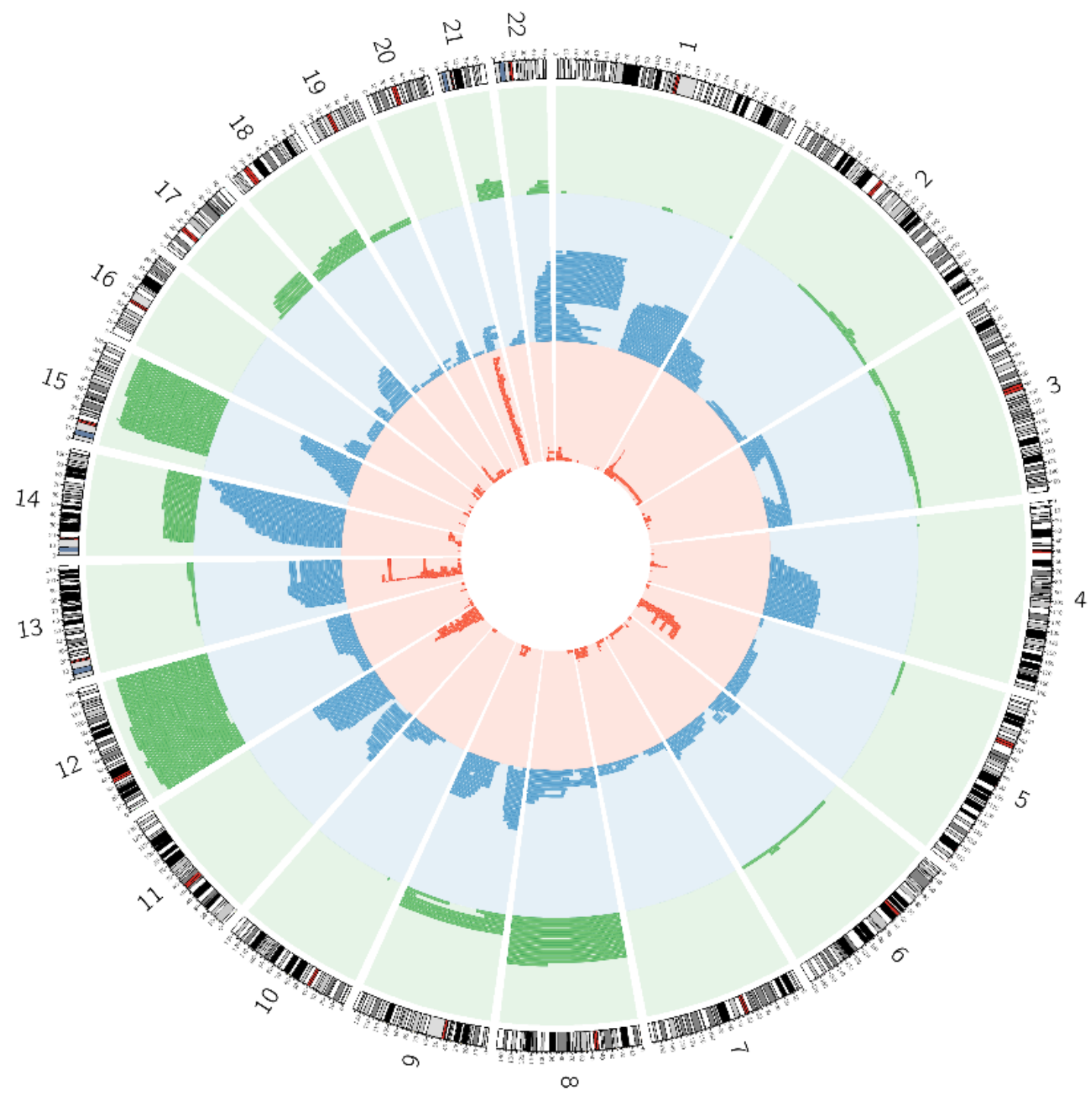

10. In Figure 4A, the chromosome arm legend is not useful. It is does not add any value to the results and conclusion as the chromosome number is not shown. It may not be necessary to show the chromosome arms.

The chromosome arm label has been removed, and the shapes of the points have been changed. The revised figure is below:

A.

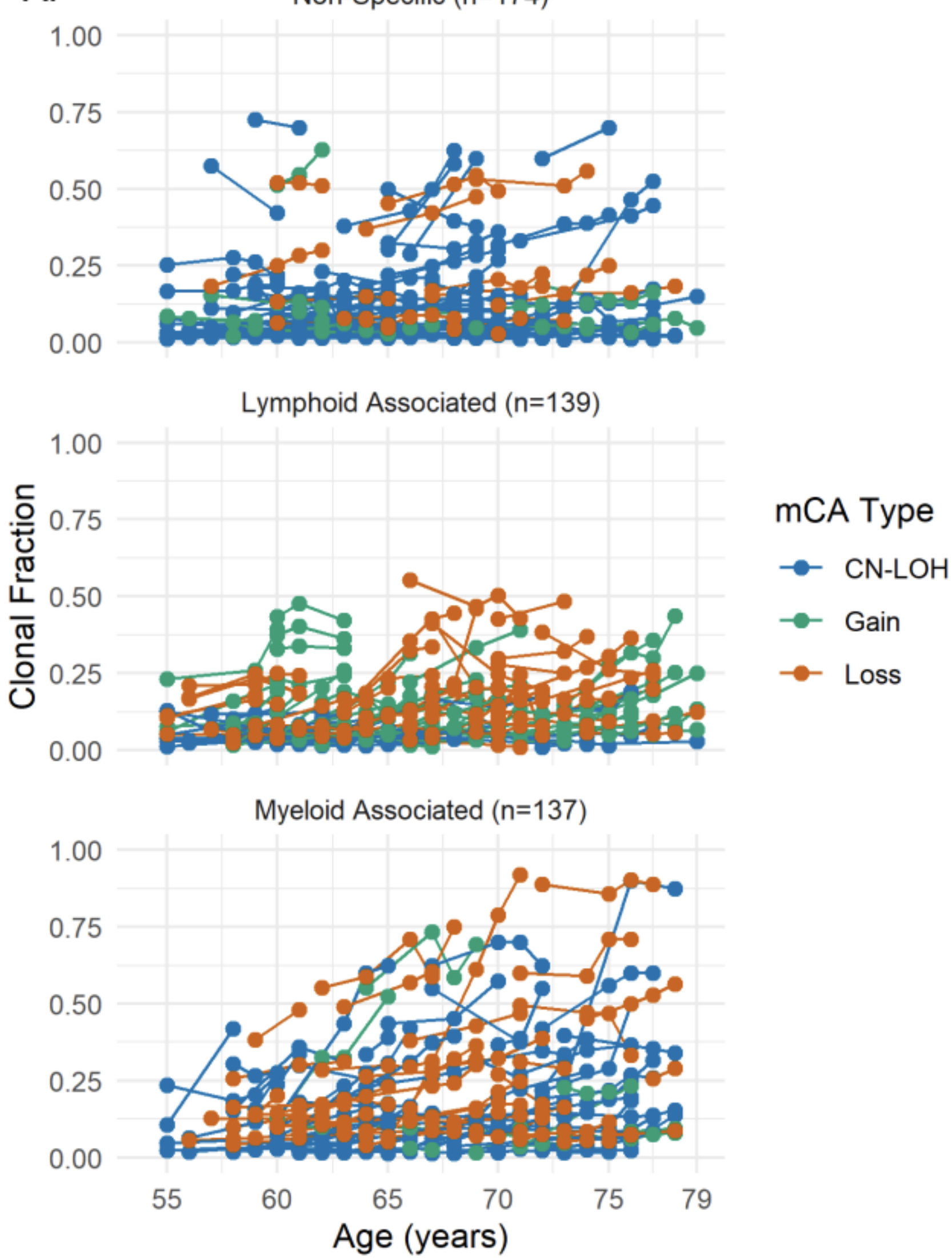
Non-Specific (n=174)
Lymphoid Associated (n=139)
Myeloid Associated (n=137)
Clonal Fraction
1.00
0.75
0.50
0.25
0.00
55
60
65
70
75
79
Age (years)
mCA Type
CN-LOH
Gain
Loss

Response to reviewers 5/20/26

REVIEWERS' COMMENTS

Reviewer #1 (Remarks to the Author):

I appreciate the thorough revisions the authors made to the manuscript, especially the additional statistical analyses and the discussion addressing the limitations of this study.

Reviewer #2 (Remarks to the Author):

The author has addressed all of my comments and I have no further concerns.

Reviewer #3 (Remarks to the Author):

I co-reviewed this manuscript with one of the reviewers who provided the listed reports. This is part of the Nature Communications initiative to facilitate training in peer review and to provide appropriate recognition for Early Career Researchers who co-review manuscripts.

Reviewer #4 (Remarks to the Author):

The authors have comprehensively responded to my previous comments. I have no further comments.

Reviewer #5 (Remarks to the Author):

I thank the authors for the revision and thoroughly addressing my comments. The manuscript presents interesting findings regarding clonal hematopoiesis driven by mosaic chromosomal alterations.

I wonder if the authors have published their codes. I could not find the information in the Data availability statement. It is recommended to publish the codes with a permanent doi.

Thank you for mentioning the Data availability statement. We have updated the statement to include how to access the genotyping array data on dbGAP and included the permanent doi for the analysis pipeline. Details on the packages for applying linear mixed models is included in the methods section.

“Genotyping array data are available on the public repository dbGaP under the study accession: phs001286.v4.p2. The informed consent document signed by the PLCO trial participants allows the use of these data by investigators for discovery and hypothesis generation in the investigation of genetic contributions to cancer, adult diseases and novel GWAS methods. Companion phenotype data are available upon request and approval of proposal submitted to the PLCO Cancer Data Access System (CDAS). (https://cdas.cancer.gov/learn/plco/instructions/). The analytic pipeline used for MoChA analysis is available at mocha.wdl.”
